# Prevalence of pre-clinical and clinical obesity in adults: Pooled analysis of 56 population-based national health surveys

**DOI:** 10.1371/journal.pgph.0004838

**Published:** 2025-07-24

**Authors:** Rodrigo M. Carrillo-Larco, Wilmer Cristobal Guzman-Vilca, Carla Tarazona-Meza, Xiaolin Xu, Antonio Bernabe-Ortiz

**Affiliations:** 1 Hubert Department of Global Health, Rollins School of Public Health, Emory University, Atlanta, Georgia, United States of America; 2 CRONICAS Centre of Excellence in Chronic Diseases, Universidad Peruana Cayetano Heredia, Lima, Peru; 3 International Health, Johns Hopkins Bloomberg School of Public Health, Baltimore, Maryland, United States of America; 4 Universidad Cientifica del Sur, Lima, Peru; 5 School of Public Health, The Second Affiliated Hospital, Zhejiang University School of Medicine, Hangzhou, Zhejiang, China; 6 School of Public Health, Faculty of Medicine, The University of Queensland, Brisbane, Queensland, Australia; Qatar University College of Medicine, QATAR

## Abstract

Obesity is commonly defined using body mass index (BMI), but BMI alone does not capture the metabolic and functional consequences of excess weight. We examined the prevalence of clinical obesity, a new definition that incorporates BMI alongside metabolic and functional impairments. We analyzed nationally representative surveys. Clinical obesity was defined as BMI ≥ 30 kg/m^2^ and waist-to-height ratio ≥0.5 or BMI ≥ 40 kg/m^2^ with at least: self-reported diabetes, fasting plasma glucose ≥126 mg/dl, self-reported hypertension, blood pressure ≥140/90 mmHg, or total cholesterol ≥200 mg/dl. We estimated the survey-weighted and age-standardized prevalence of clinical obesity and BMI-only obesity by country and sex. Data from 56 countries were included (n = 142,250). The prevalence of clinical obesity ranged between 0% and 29%. The prevalence of clinical obesity was < 10% in 41 countries for men and 30 for women. In men, the largest shift in prevalence of BMI-only obesity and clinical obesity was observed in Malawi (0.7% vs 0.2%, relative change: -68%); in women, the largest shifts in prevalence were seen in Malawi (5.6% vs. 2.6%, relative change: -53%) and Rwanda (2.7% vs. 1.3%, relative change: -52%). The adoption of clinical obesity criteria revises obesity prevalence estimates and highlights metabolic and functional impairments beyond BMI. Our results emphasize the need to carefully consider how obesity is defined in population surveillance to ensure its relevance to health outcomes.

## Introduction

Obesity is a well-established risk factor for many non-communicable diseases, including diabetes, cardiovascular and kidney diseases, and certain cancers [1–6]. The study of obesity has garnered significant attention from researchers and public health officials responsible for developing global monitoring frameworks. To date, obesity global surveillance efforts have solely relied on body mass index (BMI) as a measure of adiposity [7]. These surveillance initiatives typically report mean BMI estimates by country and sex, along with prevalence estimates of overweight (BMI ≥ 25 kg/m^2^) and obesity (BMI ≥ 30 kg/m^2^) [7]. However, the recent revision of the clinical obesity definition has disrupted the established paradigm and poses challenges to the global monitoring of obesity [8].

While there is substantial data on the prevalence of obesity defined solely by BMI [7], given the novelty of the clinical obesity definition [8], there is a lack of data on the prevalence of clinical obesity. This gap prevents understanding to what extent the prevalence of obesity may shift if the clinical obesity definition is adopted. This is crucial because the revised definition is stricter [8], potentially reducing the number of individuals previously classified as obese using the BMI-only criterion. This has implications for disease burden estimation, public health planning, and interventions for weight management. Moreover, quantifying the prevalence of clinical obesity in multiple countries might also prompt discussions on how to define clinical obesity in population-based health surveys or global monitoring frameworks, where comprehensive data on multiple cardiometabolic conditions, necessary for ascertaining clinical obesity, are not readily available. To fill these gaps, we aggregated several nationally representative health surveys, proposed a pragmatic definition of clinical obesity, and computed its country- and sex-specific prevalence.

## Methods

### Ethics statement

We analyzed deidentified open-access data [9]. No human subjects were directly involved in this research. We classified this work as of minimal risk and did not seek approval from an Ethics Committee.

### Data sources

We conducted an empirical analysis of nationally representative health surveys accessible through the WHO STEPS Survey repository (Table A in S1 Text) [9]. The WHO STEPS surveys adhere to a standardized sampling protocol and similar procedures across countries [9]. The WHO STEPS surveys comprise three phases [9]. The initial phase collects socio-demographic and health-related data. The second phase collects anthropometric (weight, height, waist circumference) and blood pressure measurements. The third phase collects blood biomarkers (fasting plasma glucose and total cholesterol).

We employed the most recent survey available for each country ranging from 2004 to 2020 (54% conducted between 2015–2020). We incorporated surveys that provided objective measurements of weight, height, waist circumference, blood pressure, fasting plasma glucose, and total cholesterol. These anthropometric, clinical and blood markers are crucial for defining clinical obesity [8]. While the clinical obesity definition encompasses a broader list of cardiometabolic conditions beyond high blood pressure, glucose and total cholesterol [8], we only included these three due to data availability. Arguably, at the population level across multiple countries, obtaining data on a more extensive list of cardiometabolic conditions would be challenging and not currently available for monitoring and surveillance of clinical obesity [7,10–12].

### Study population

We included non-pregnant adults aged 25 and 64 years, aligning with the age range originally targeted by the WHO STEPS surveys [8]. Consistent with global analyses and the need for high-quality data, we excluded observations whose systolic blood pressure was outside the range of 70–270 mmHg [11], diastolic blood pressure outside the range of 30–150 mmHg [11], BMI outside the range of 10–80 kg/m^2^ [7], waist-to-height ratio outside the range of 0.2 and 2.0, [7] total cholesterol outside the range of 67–773 mg/dl [10], and fasting plasma glucose outside the range of 36–540 mg/dl [12].

Regarding the key variables for the analysis, sampling design, sex, age, pregnancy status, height, weight, waist circumference, blood pressure, total cholesterol, fasting plasma glucose, self-reported history of hypertension and diabetes, we conducted a complete-case analysis. While there were significant differences between the included and excluded observations, the differences were not clinically meaningful and most likely expected based on variability (Table B in S1 Text). The largest differences were detected in fasting plasma glucose (91 mg/dl in the included observations and 94 mg/dl in the excluded observations) and total cholesterol (165 mg/dl in the included observations and 172 mg/dl in the excluded observations).

### Variables

#### Outcome.

The outcome of interest was clinical obesity [8]. Our definition encompassed three levels: clinical obesity, pre-clinical obesity, and people who did not meet the criteria for either. To meet the new definition for clinical obesity, it is required both 1) the confirmation of obesity status (e.g., with anthropometric measures) and 2) evidence of reduced organ function (e.g., with tests showing abnormalities) or daily activity limitations due to obesity [8]. In this work, among people with a BMI ≥ 30 kg/m^2^ and a waist-to-height ratio ≥0.5 or BMI ≥ 40 kg/m^2^, those who additionally self-reported diabetes or hypertension, had systolic blood pressure ≥140 mmHg, diastolic blood pressure ≥90 mmHg, fasting plasma glucose ≥126 mg/dl, or total cholesterol ≥200 mg/dl, were classified as clinical obese. People with a BMI ≥ 30 kg/m^2^ and a waist-to-height ratio ≥0.5 or BMI ≥ 40 mg/k^2^, but without self-reported diabetes or hypertension, and had blood pressure, glucose and total cholesterol below the given thresholds, were classified as pre-clinical obese. Readers should not interpret individuals who did not meet the above criteria as non-obese. For example, the pre-clinical obesity group required both a BMI ≥ 30 kg/m^2^ and a waist-to-height ratio ≥0.5. Therefore, individuals with a BMI ≥ 30 kg/m^2^ but a waist-to-height ratio below 0.5 would be considered obese under the traditional BMI-only definition but would not meet the composite criteria used in this analysis. Finally, we also classified people according to the BMI-only criteria as: [7] underweight (BMI < 18.5 kg/m^2^), normal weight (BMI between 18.5 and <25 kg/m^2^), overweight (BMI between 25 and <30 kg/m^2^), and obesity (BMI ≥ 30 kg/m^2^).

The definition of clinical obesity proposed by the consensus group also includes limitations in activities of daily living as one of its criteria. Specifically, the consensus paper reads, “*The diagnosis of Clinical Obesity requires: a. Clinical confirmation of obesity status by anthropometric criteria or by direct body fat measurement, plus one or both of the following criteria: b. Evidence of reduced organ/tissue function due to obesity (ie, signs, symptoms and/or diagnostic tests showing abnormalities in the function of one or more tissue/organ system), c. Significant, age-adjusted limitations of day-to-day activities reflecting the specific impact of obesity on mobility and/or other basic Activities of Daily Living (ADL=bathing, dressing, toileting, continence, eating)*” [8]. However, such data were not available in our dataset; therefore, our operationalization of clinical obesity was restricted to individuals with confirmed anthropometric obesity accompanied by evidence of cardiometabolic dysfunction.

For the BMI criterion in the clinical obesity definition, we used the global standards (e.g., BMI ≥ 30 kg/m^2^) that have been employed for decades in global monitoring frameworks and research [7]. While the new clinical obesity definition acknowledges ethnic-specific BMI thresholds [8], we applied a universal cut-off to assess how obesity prevalence, as traditionally measured in major global initiatives [7], compares to the new clinical obesity definition [8]. Our goal was to examine how the current understanding of obesity prevalence —as imperfect it may be— might shift with the adoption of clinical obesity criteria.

#### Other variables.

For descriptive purposes, we utilized country and world region as well as sex (men/women). Countries were grouped according to their respective WHO regions. Consistent with similar global analyses [7,10–16], we prioritized sex-specific results over age-specific results.

### Statistical analysis

In the pooled dataset involving all countries, we conducted a descriptive analysis to quantify the prevalence of clinical and pre-clinical obesity accounting for the complex survey design. A complex survey design refers to any sampling strategy that departs from simple random sampling. Such designs typically incorporate elements like stratification, clustering, unequal probability weights, and finite population corrections. Complex survey designs are commonly employed in large-scale national or multinational surveys, as they offer greater efficiency and cost-effectiveness compared to simple random sampling.

Prevalence estimates accounted for the complex survey design of each dataset, using Stata’s *svy* command to incorporate strata, primary sampling units, and sampling weights. Moreover, prevalence estimates were derived using the *subpop* specification by country and sex. Similarly, we computed the prevalence of BMI categories for each country and sex accounting for the complex survey design. As requested by the editor, the design and misspecification effects are presented (Table D in S1 Text). Finally, all results were age-standardized using the WHO standard population as the reference. Specifically, the sex- and country-specific estimates—computed using the *svy* command in Stata—were standardized using the WHO standard population as the reference.

At the country level, we computed the relative and absolute change in obesity prevalence between the BMI-only definition and the clinical obesity definition. For transparency and completeness, we calculated both the relative and absolute changes. The absolute change is expressed as the difference between the prevalence of BMI-only obesity and the prevalence of clinical obesity, expressed as percentage points. The relative change is calculated using the following formula. The relative change is expressed as a percentage.


$$\;\frac{\left(Prevalence\;of\;Clinical\;Obesity-Prevalence\;of\;BMI\;only\;Obesity\right)}{Prevalence\;of\;BMI\;only\;Obesity}$$

Please note that we did not use the absolute number of the numerator to ensure that the percentage is negative, which emphasizes the nature of a negative change or reduction from the prevalence of BMI-only obesity to the prevalence of clinical obesity. Data management and figure creation were conducted using R (4.4.1), while statistical analyses were performed in Stata (19.0 SE). Readers are kindly reminded that this is a cross-sectional analysis of multiple nationally representative surveys; thus, “change” refers to differences in obesity prevalence when applying the standard versus the new clinical definition, not to longitudinal changes within individuals over time. Finally, in line with our stated aims—which did not include reporting a single global prevalence—our results are presented separately for each country and are not aggregated across countries. This approach is relevant for country-specific readers who may wish to leverage these findings for local decision-making and policy development.

### Role of the funding source

There was no specific funding for this work.

## Results

### Study population

Data from 56 countries across six world regions (n = 142,250) were included (Flowchart A in S1 Text). These regions are Africa (n = 49,438 from 18 countries), the Americas (n = 3,083 from one country), the Eastern Mediterranean (n = 19,292 from nine countries), Europe (n = 17,536 from seven countries), Southeast Asia (n = 27,334 from six countries), and the Western Pacific (n = 25,567 from 15 countries). Across countries, the unweighted mean age was 42.4 years (standard deviation: 11.0), and the unweighted frequency of females was 60.5% (Table C in S1 Text).

### Clinical obesity (survey-weighted and age-standardized)

**Fig 1:**
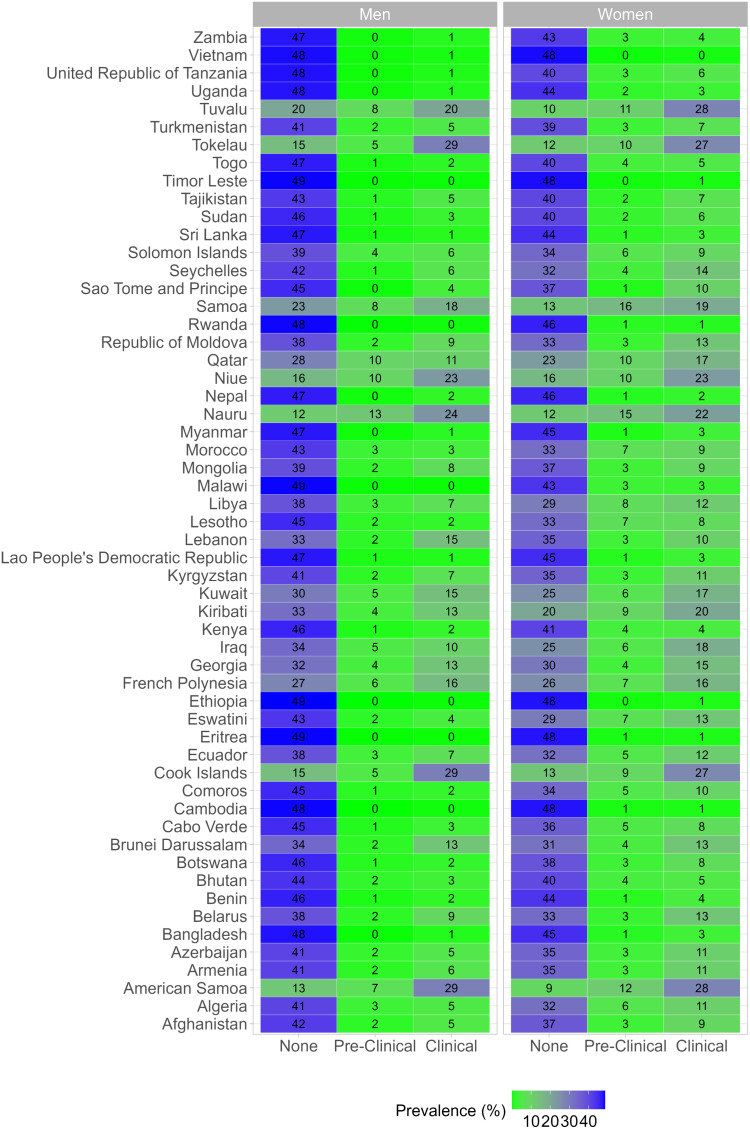
Survey-adjusted and age-standardized prevalence of clinical obesity by country and sex.

At the national level, the prevalence of clinical obesity in men ranged from <1% in Timor Leste, Rwanda, Malawi, Ethiopia, Eritrea, and Cambodia, to 29% in American Samoa, Cook Islands, and Tokelau (Fig 1). A similar pattern was observed in women, with a prevalence of clinical obesity as low as ≤1% in Vietnam, Timor Leste, Rwanda, Ethiopia, Eritrea, and Cambodia, and as high as 28% in American Samoa and Tuvalu.

The underlying results are available in Table D in S1 Text. These results account for the complex survey design and were age-standardized following the WHO reference population.

Among men, the age-standardized prevalence of clinical obesity was < 10% in 41 countries, most of which were in Africa (18/41) (Table 1). Among women, the age-standardized prevalence of clinical obesity was < 10% in 30 countries, most of them in Africa as well (14/30).

**Table 1:** Number of countries in discrete categories of the age-standardized prevalence of clinical obesity.

Survey-adjusted prevalence of clinical obesity	Sex	Number of countries	Regions
<10%	Men	41	Africa (18), Americas (1), Eastern Mediterranean (5), Europe (6), Southeast Asia (6), Westen Pacific (5)
10%-29%		15	Easten Mediterranean (4), Europe (1), Western Pacific (10)
30%-49%		0	
≥50%		0	
<10%	Women	30	Africa (14), Easten Mediterranean (3), Europe (2), Southeast Asia (6), Westen Pacific (5)
10%-29%		26	Africa (4), Americas (1), Eastern Mediterranean (6), Europe (5), Westen Pacific (10)
30%-49%		0	
≥50%		0	

### Clinical obesity versus BMI-only obesity (survey-weighted and age-standardized)

The prevalence of obesity based on BMI only was higher across all countries compared to the prevalence of clinical obesity, which adheres to a more stringent definition. [8] Nonetheless, there were regional and national variations (Figure A in S1 Text).

**Fig 2:**
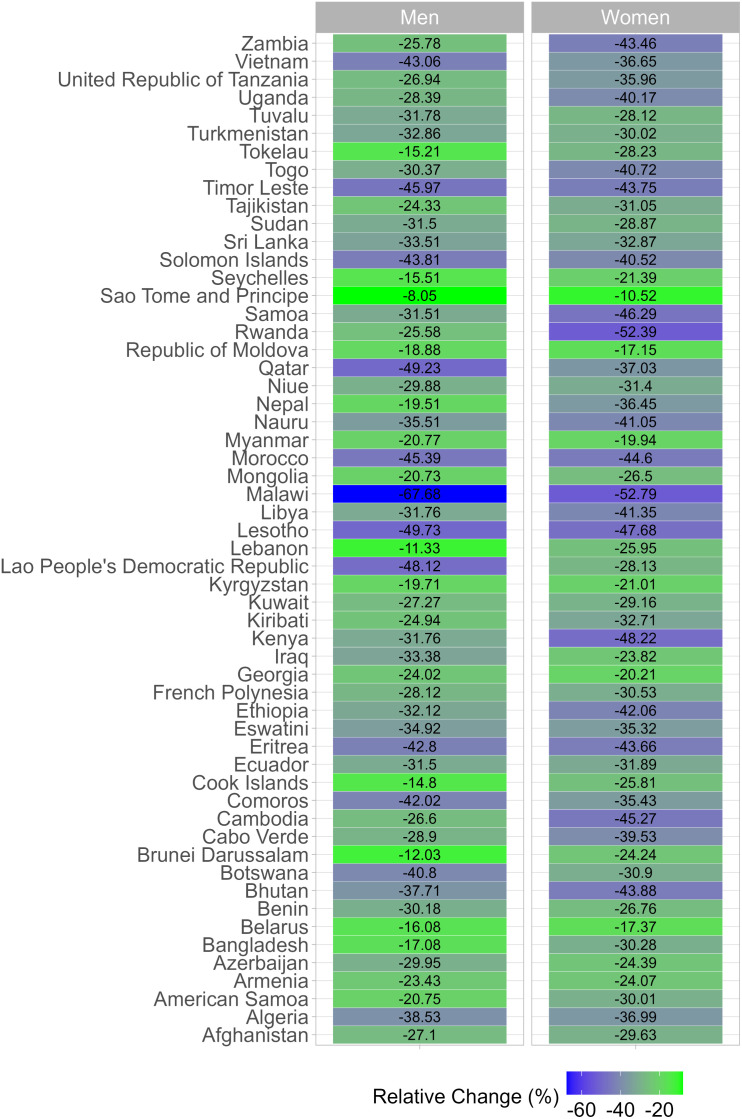
Relative change between the age-standardized prevalence of BMI-only obesity and the age-standardized prevalence of clinical obesity by country and sex.

**Fig 3:**
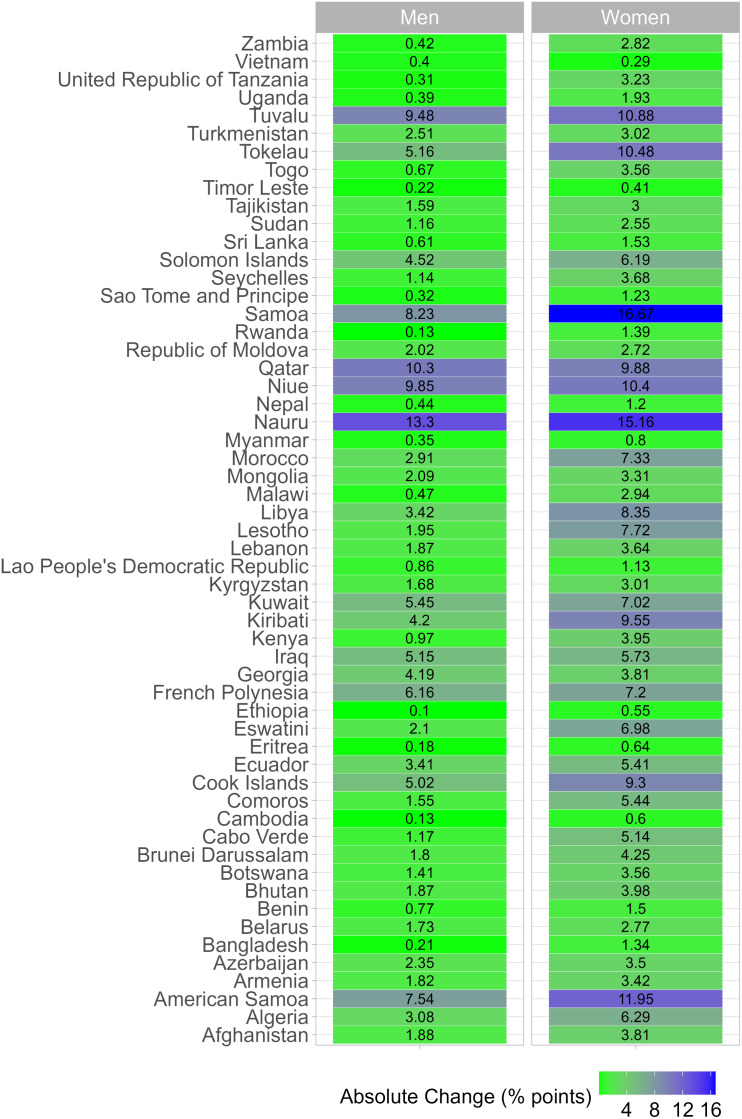
Absolute change between the age-standardized prevalence of BMI-only obesity and the age-standardized prevalence of clinical obesity by country and sex.

In men, the largest shift in prevalence was observed in Malawi, where the prevalence of BMI-only obesity was 0.7% and the prevalence of clinical obesity was 0.2% (-67.7% relative reduction) (Fig 2); note that although the relative change in prevalence was substantial, the absolute change was negligible (<1 percentage point; Fig 3). A relative change of ≥10% and an absolute change of ≥10 percentage points were documented in Nauru (-35.5% relative change and 13.3 percentage points in absolute change; prevalence of clinical obesity was 24.2% and that of BMI-only obesity was 37.5%), and Qatar (-49.2% and 10.3; prevalence of clinical obesity was 10.6% and that of BMI-only obesity was 20.9%).

These results are also available in Table E in S1 Text. Underlying prevalence estimates were computed accounting for the complex survey design and age-standardized using the WHO reference population.

In women, the relative change in prevalences exceeded 50% in Malawi (5.6% for BMI-only obesity and 2.6% for clinical obesity; -52.8% relative reduction) and Rwanda (2.7% for BMI-only obesity and 1.3% for clinical obesity; -52.4% relative reduction). For Malawi and Rwanda, the absolute change was 2.9 and 1.4 percentage points, respectively. Countries were both the relative change and the absolute change exceeded 10% and 10 percentage points were in the Western Pacific (American Samoa, Nauru, Niue, Samoa, Tokelau and Tuvalu).

## Discussion

### Main findings

This study provides nationally representative estimates of clinical obesity prevalence across 56 countries using a pragmatic definition based on available survey data. As expected, the findings indicate that clinical obesity is less prevalent than BMI-based obesity, and the adoption of clinical obesity as a classification criterion reduces the proportion of individuals considered obese, with variability across countries and regions. We also documented large relative and absolute changes in prevalence estimates between the BMI-only and clinical definitions of obesity. From a population health perspective, these findings underscore that adopting a new definition for a major health issue such as obesity warrants careful consideration and thorough analysis of its potential ramifications, as shifts in prevalence estimates can be substantial. Regardless of how obesity is defined, the importance of primordial prevention cannot be overstated and should remain a central priority across countries and populations.

### Implications

As with BMI-only obesity [7], we documented variations in clinical obesity prevalence, highlighting the complex interplay of environmental, behavioral, and biological determinants of adiposity [17]. While well-established drivers of high BMI—unhealthy diets, physical inactivity, early-life factors, and environmental risks—remain central [17], the inclusion of clinical markers in obesity classification introduces additional determinants that could help explain prevalence differences across populations, where the underlying distribution of other cardiometabolic conditions also differs [7,10–12]. For example, since diabetes and blood glucose levels might be considered components of the clinical obesity definition, populations where impaired glucose metabolism occurs at lower BMI levels may exhibit a different distribution of clinical obesity compared to BMI-based obesity [18,19]. This phenomenon has been observed in certain ethnic groups in whom metabolic dysfunction develops at lower BMI thresholds [18]. Similarly, the inclusion of hypertension and elevated blood pressure in the clinical obesity definition means that dietary factors beyond those influencing BMI, such as high salt consumption, could also contribute to obesity classification.

The observed differences between clinical obesity and BMI-based obesity suggest that BMI alone overestimates the burden of obesity-related organ dysfunction. This has implications for disease burden estimation, resource allocation, public health policy and clinical practice. For example, while many individuals classified as obese based on BMI alone may not require pharmacological treatment and could rely on non-pharmacological interventions—assuming they do not have other health conditions— [20,21], arguably everyone meeting the criteria for clinical obesity may benefit from pharmacological treatment to manage blood pressure or blood glucose, at least. This also implies that there is little to no opportunity for primary prevention of clinical obesity, as its definition already includes a cardiometabolic condition that most likely warrants secondary prevention or treatment. Moreover, public health systems that rely on BMI-based prevalence estimates may need to reassess obesity-related disease burden projections, particularly in settings where metabolic dysfunction is common at lower BMI levels [18,19].

The findings also highlight the challenges of transitioning from a BMI-based obesity surveillance system to one incorporating functional and metabolic markers. While the revised clinical obesity definition aligns obesity classification more closely with health risks [1–6,8], its feasibility in large-scale population surveys remains a concern due to data limitations [7]. It is imperative for experts and global health organizations to establish a pragmatic definition for measuring clinical obesity in settings where data on cardiometabolic conditions may be limited. Without a standardized approach, the growing body of evidence may become fragmented, with studies adopting different definitions based on data availability.

These observations open a series of critical research questions. What are the primary determinants of clinical obesity, and to what extent is it driven by anthropometric measures versus metabolic dysfunction? Are certain populations more prone to clinical obesity due to genetic predispositions, early-life exposures, or dietary patterns that influence metabolic health independently of BMI? Additionally, how does clinical obesity relate to long-term health outcomes compared to BMI-only obesity? Answering these questions will be essential to refining obesity classification systems, improving risk prediction, and developing more effective public health strategies.

### Strengths and limitations

Our study analyzed population-based national health surveys from multiple countries, adhering to a strict and internationally recognized protocol to ensure high-quality data. We developed a pragmatic definition of clinical obesity that maximizes the use of readily available population health data. To generate robust estimates, we accounted for the complex survey design in our prevalence calculations, ensuring our findings reflect the prevalence of clinical obesity in each country. Our prevalences of obesity as per BMI only were consistent with global metrics (Table E in S1 Text) [7]. This is the first study to estimate the prevalence of clinical obesity under this new definition across multiple countries, marking a significant step forward in global obesity research [8].

There are, however, limitations to acknowledge. First, while our pragmatic definition of clinical obesity aligns with the core principles established by the expert panel that coined this concept, it does not incorporate the full spectrum of cardiometabolic conditions they recommended and does not incorporate features of limitations in daily activities [8]. This was a necessary trade-off to leverage available population-based health data, as few national health surveys currently collect all the required variables. Without this pragmatic approach, many countries would lack any estimate of clinical obesity prevalence. This decision will likely underestimate the true prevalence of clinical obesity, as it does not account for the full spectrum of cardiometabolic conditions. Thus, the true prevalence of clinical obesity remains uncertain. Second, our complete-case analysis suggests that included participants were slightly healthier than those excluded. This raises the possibility that the true prevalence of clinical obesity may be higher than our estimates, underscoring the need for continued research and improved data collection efforts. Third, a major limitation, our analysis included data from 56 countries. While this represents a substantial contribution, it still covers only a fraction of the world’s nations and territories. Moreover, for some regions such as the Americas, we included very few countries. Future research should expand this effort by incorporating additional surveys, such as the Demographic and Health Surveys (DHS) or other national health surveys, or by designing new epidemiological studies to assess the burden of clinical obesity in a broader range of countries [7,10–12]. Additionally, expert panels should consider defining a pragmatic version of clinical obesity that can be applied in population-based studies, particularly in low- and middle-income countries where comprehensive biomarker and cardiometabolic data may not be available. Until data from more countries—particularly those with larger populations and a substantial obesity burden—become available, our findings should be considered a preliminary multi-country snapshot rather than a globally representative analysis. Fourth, the data we analyzed were collected in different years. As a result, our prevalence estimates should be interpreted in the context of the data collection periods, as more recent prevalence rates may differ from those based on older datasets. For countries with older datasets—those collected ten or more years ago—our prevalence estimates may underestimate the current burden of obesity, as its prevalence has increased in most countries over time [7]. Fifth, we used total cholesterol rather than LDL cholesterol, which is the primary target of most clinical guidelines for preventing cardiovascular events. This choice was driven by pragmatic considerations, as data on LDL cholesterol are much more limited in WHO STEPS surveys and many national health surveys as well as population-based epidemiological studies worldwide [10]. Sixth, our study’s descriptive and cross-sectional nature prevents us from assessing the long-term consequences of clinical obesity. However, given the well-established long-term health risks associated with elevated BMI and the additional cardiometabolic conditions included in clinical obesity, we argue that long-term outcome analyses may not yield fundamentally different conclusions. Instead, our primary contribution lies in providing the first multinational estimates of clinical obesity prevalence. Seventh, we stand by our decision to use a universal BMI threshold, as this aligns with current global estimates and enables a comparison of how the new clinical obesity definition differs from the *status quo* [7]. However, regions and countries with specific BMI thresholds should apply our pragmatic approach while incorporating their population-specific BMI cutoffs to ensure more contextually relevant estimates. Finally, our objective was to leverage nationally representative health surveys to produce the first estimates of clinical obesity in countries where appropriate data were available—despite variation in the years of data collection. We did not aim to generate estimates for all countries or harmonize data across time, which would require a different methodological approach and represent a substantial undertaking. Importantly, country-specific results provide the greatest utility for national policymakers and public health stakeholders, who rely on context-specific data to guide the design of targeted interventions and allocate resources appropriately. These estimates are also highly valuable for local researchers, enabling them to prioritize public health challenges, support grant applications, and design follow-up studies tailored to the specific epidemiological and contextual characteristics of their populations.

## Conclusions

The adoption of a clinical obesity definition alters obesity prevalence estimates compared to BMI-based classifications. Our results emphasize the need to carefully consider how obesity is defined in population surveillance to ensure its relevance to health outcomes. While the clinical obesity framework offers a more precise measure of obesity-related disease burden [8], its implementation in routine surveillance will require further adaptation to overcome data availability challenges. Future efforts should explore pragmatic solutions for integrating functional and metabolic health markers into global obesity monitoring frameworks.

## Supporting information

S1 Text Figure A in S1 Text. Survey-adjusted and age-standardized prevalence of clinical obesity versus prevalence of BMI-only obesity by country and sex. Table A in S1 Text. Countries included in the analysis, their world region and when (year) data was collected. Table B in S1 Text. Comparison of included and excluded observations in the analysis. Table C in S1 Text. Description of the study sample by country. Table D in S1 Text. Survey-adjusted as well as age-standardized prevalence (%) of clinical obesity. Table E in S1 Text. Survey-adjusted and age-standardized prevalence of clinical obesity and BMI-only obesity together with the relative change as well as the absolute change, and the best-available evidence of the prevalence of BMI-only obesity worldwide from the NCD-RisC for comparison purposes. Flowchart A in S1 Text. Analytical sample.
(DOCX)
