# Supplementary material for: Prevalence of pre-clinical and clinical obesity in adults: Pooled analysis of 56 population-based national health surveys
Source: PLOS Glob Public Health. 2025 Jul 24;5(7):e0004838. doi: 10.1371/journal.pgph.0004838 (PMC12289025; doi:10.1371/journal.pgph.0004838)
Supplement: S1 Text — Survey-adjusted and age-standardized prevalence of clinical obesity versus prevalence of BMI-only obesity by country and sex. Table A in S1 Text. Countries included in the analysis, their world region and when (year) data was collected. Table B in S1 Text. Comparison of included and excluded observations in the analysis. Table C in S1 Text. Description of the study sample by country. Table D in S1 Text. Survey-adjusted as well as age-standardized prevalence (%) of clinical obesity. Table E in S1 Text. Survey-adjusted and age-standardized prevalence of clinical obesity and BMI-only obesity together with the relative change as well as the absolute change, and the best-available evidence of the prevalence of BMI-only obesity worldwide from the NCD-RisC for comparison purposes. Flowchart A in S1 Text. Analytical sample. (DOCX) [file pgph.0004838.s001.docx]

**Prevalence of pre-clinical and clinical obesity in adults: Pooled analysis of 56 population-based national health surveys**

**Contents**

[S1 Figure. Survey-adjusted and age-standardized prevalence of clinical obesity versus prevalence of BMI-only obesity by country and sex. 3](#_Toc197941545)

[S1 Table. Countries included in the analysis, their world region and when (year) data was collected. 4](#_Toc197941546)

[S2 Table. Comparison of included and excluded observations in the analysis. 5](#_Toc197941547)

[S3 Table. Description of the study sample by country. 6](#_Toc197941548)

[S4 Table. Survey-adjusted as well as age-standardized prevalence (%) of clinical obesity (Figure 1 in manuscript) and BMI-only categories. 14](#_Toc197941549)

[S5 Table. Survey-adjusted and age-standardized prevalence of clinical obesity and BMI-only obesity together with the relative change (Figure 2 in manuscript) as well as the absolute change, and the best-available evidence of the prevalence of BMI-only obesity worldwide from the NCD-RisC for comparison purposes. 23](#_Toc197941550)

[S1 Flowchart. Analytical sample. 25](#_Toc197941551)

# **Figure A in S1 Text. Survey-adjusted and age-standardized prevalence of clinical obesity versus prevalence of BMI-only obesity by country and sex.**


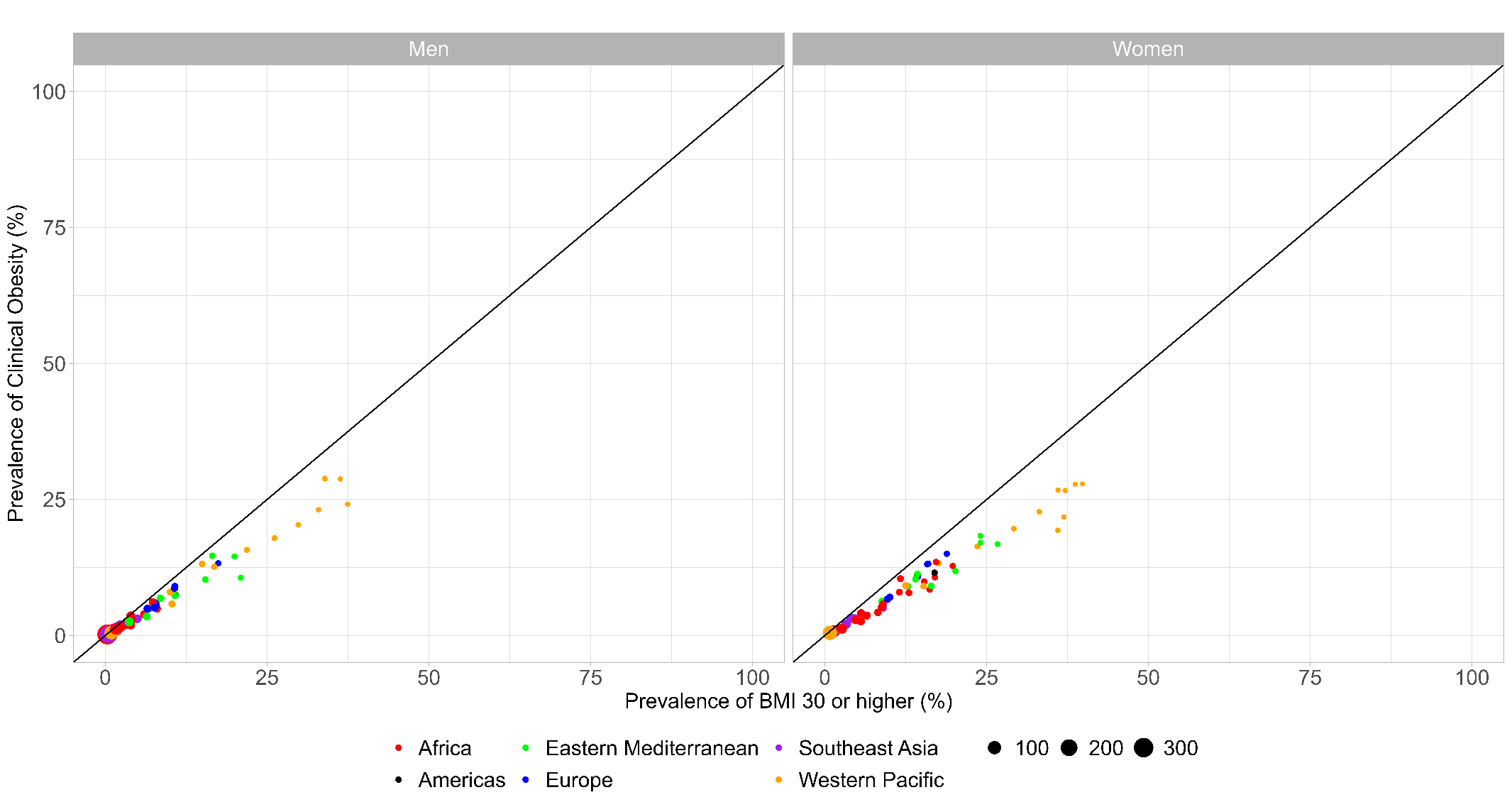


The size of the dots corresponds to the magnitude of the ratio between the relative and absolute change in absolute terms. That is, a higher ratio represents that the relative change was much higher than the absolute change. The underlying results are available in Supplementary Table 5, together with the relative change and absolute change between clinical obesity and prevalence of BMI-only obesity. These results account for the complex survey design of each WHO STEPS survey and were age-standardized taking as reference the WHO standard population.

# **Table A in S1 Text. Countries included in the analysis, their world region and when (year) data was collected.**

| **Country** | **Region** | **Data year** |
| --- | --- | --- |
| Afghanistan | Eastern Mediterranean | 2018 |
| Algeria | Africa | 2016 |
| American Samoa | Western Pacific | 2004 |
| Armenia | Europe | 2016 |
| Azerbaijan | Europe | 2017 |
| Bangladesh | Southeast Asia | 2018 |
| Belarus | Europe | 2016 |
| Benin | Africa | 2015 |
| Bhutan | Southeast Asia | 2019 |
| Botswana | Africa | 2014 |
| Brunei Darussalam | Western Pacific | 2015 |
| Cabo Verde | Africa | 2020 |
| Cambodia | Western Pacific | 2010 |
| Comoros | Africa | 2011 |
| Cook Islands | Western Pacific | 2013 |
| Ecuador | Americas | 2018 |
| Eritrea | Africa | 2010 |
| Eswatini | Africa | 2014 |
| Ethiopia | Africa | 2015 |
| French Polynesia | Western Pacific | 2010 |
| Georgia | Europe | 2016 |
| Iraq | E,astern Mediterranean | 2015 |
| Kenya | Africa | 2015 |
| Kiribati | Western Pacific | 2015 |
| Kuwait | Eastern Mediterranean | 2014 |
| Kyrgyzstan | Eastern Mediterranean | 2013 |
| Lao People's Democratic Republic | Western Pacific | 2013 |
| Lebanon | Eastern Mediterranean | 2017 |
| Lesotho | Africa | 2012 |
| Libya | Eastern Mediterranean | 2009 |
| Malawi | Africa | 2017 |
| Mongolia | Western Pacific | 2019 |
| Morocco | Eastern Mediterranean | 2017 |
| Myanmar | Southeast Asia | 2014 |
| Nauru | Western Pacific | 2015 |
| Nepal | Southeast Asia | 2019 |
| Niue | Western Pacific | 2011 |
| Qatar | Eastern Mediterranean | 2012 |
| Republic of Moldova | Europe | 2013 |
| Rwanda | Africa | 2012 |
| Samoa | Western Pacific | 2013 |
| Sao Tome and Principe | Africa | 2019 |
| Seychelles | Africa | 2004 |
| Solomon Islands | Western Pacific | 2015 |
| Sri Lanka | Southeast Asia | 2014 |
| Sudan | Eastern Mediterranean | 2016 |
| Tajikistan | Europe | 2016 |
| Timor Leste | Southeast Asia | 2014 |
| Togo | Africa | 2010 |
| Tokelau | Western Pacific | 2014 |
| Turkmenistan | Europe | 2018 |
| Tuvalu | Western Pacific | 2015 |
| Uganda | Africa | 2014 |
| United Republic of Tanzania | Africa | 2012 |
| Vietnam | Western Pacific | 2015 |
| Zambia | Africa | 2017 |

# **Table B in S1 Text. Comparison of included and excluded observations in the analysis.**

|  | **Complete (n=142,250)** | **Missing (n=45,119)** | **p-value** |
| --- | --- | --- | --- |
| Sex |  |  |  |
| Men | 56,155 | 19,534 | <0.001 |
| Women | 86,095 | 25,585 |  |
| Age | 42.5 | 41.8 | <0.001 |
| Body mass index (kg/m^2^) | 25.4 | 26.2 | <0.001 |
| Waist-to-height ratio | 0.53 | 0.54 | <0.001 |
| Systolic blood pressure (mmHg) | 126.8 | 127.5 | <0.001 |
| Diastolic blood pressure (mmHg) | 81.4 | 80.3 | <0.001 |
| Fasting plasma glucose (mg/dl) | 91.3 | 94.8 | <0.001 |
| Total cholesterol (mg/dl) | 165.8 | 172.8 | <0.001 |
| Self-reported hypertension |  |  |  |
| No | 116,487 | 34,912 | <0.001 |
| Yes | 25,763 | 7,172 |  |
| Self-reported diabetes |  |  |  |
| No | 134,662 | 39,592 | <0.001 |
| Yes | 7,588 | 2,759 |  |

P-value for Chi2 test for categorical variables t-test for numerical variables.

# **Table C in S1 Text. Description of the study sample by country.**

|  | **Sex (1=men; 2=women)** | **Age** | **Rural (0) and urban (1)** | **Current smoker (0=no; 1=yes)** | **Self-reported diabetes (0=no; 1=yes)** | **Self-reported hypertension (0=no; 1=yes)** | **Fasting plasma glucose** | **Total cholesterol** | **Body mass index** | **Waist-to-height ratio** | **Clinical obesity (0=none; 1=pre-clinical; 2=clinical)** | **BMI categories (0=underweight; 1=normal; 2=overweight; 3=obesity)** |
| --- | --- | --- | --- | --- | --- | --- | --- | --- | --- | --- | --- | --- |
| **Afghanistan** | : | : | : | : | : | : | : | : | : | : | : | : |
| **(N=2358)** | 1: 1242 (52.7%) | Mean (SD): 41.4 (10.9) | 0: 1129 (47.9%) | 0: 2127 (90.2%) | 0: 2186 (92.7%) | 0: 1759 (74.6%) | Mean (SD): 95.3 (39.0) | Mean (SD): 155 (41.8) | Mean (SD): 26.0 (5.73) | Mean (SD): 0.562 (0.108) | 0: 1906 (80.8%) | 0: 136 (5.8%) |
|  | 2: 1116 (47.3%) | Median [Min, Max]: 40.0 [25.0, 64.0] | 1: 1229 (52.1%) | 1: 231 (9.8%) | 1: 172 (7.3%) | 1: 599 (25.4%) | Median [Min, Max]: 86.0 [36.0, 400] | Median [Min, Max]: 150 [77.0, 393] | Median [Min, Max]: 25.3 [10.9, 72.2] | Median [Min, Max]: 0.558 [0.202, 1.28] | 1: 120 (5.1%) | 1: 993 (42.1%) |
|  |  |  | Missing: 0 (0%) | Missing: 0 (0%) |  |  |  |  |  |  | 2: 332 (14.1%) | 2: 754 (32.0%) |
|  |  |  |  |  |  |  |  |  |  |  |  | 3: 475 (20.1%) |
| **Algeria** | : | : | : | : | : | : | : | : | : | : | : | : |
| **(N=4776)** | 1: 2154 (45.1%) | Mean (SD): 42.8 (10.5) | 0: 1622 (34.0%) | 0: 4118 (86.2%) | 0: 4344 (91.0%) | 0: 3874 (81.1%) | Mean (SD): 101 (37.0) | Mean (SD): 169 (40.3) | Mean (SD): 27.4 (5.54) | Mean (SD): 0.575 (0.0928) | 0: 3477 (72.8%) | 0: 100 (2.1%) |
|  | 2: 2622 (54.9%) | Median [Min, Max]: 42.0 [25.0, 64.0] | 1: 3154 (66.0%) | 1: 658 (13.8%) | 1: 432 (9.0%) | 1: 902 (18.9%) | Median [Min, Max]: 93.0 [37.0, 495] | Median [Min, Max]: 165 [70.0, 500] | Median [Min, Max]: 26.8 [12.1, 69.2] | Median [Min, Max]: 0.567 [0.318, 1.23] | 1: 452 (9.5%) | 1: 1585 (33.2%) |
|  |  |  | Missing: 0 (0%) | Missing: 0 (0%) |  |  |  |  |  |  | 2: 847 (17.7%) | 2: 1780 (37.3%) |
|  |  |  |  |  |  |  |  |  |  |  |  | 3: 1311 (27.4%) |
| **American Samoa** | : | : | : | : | : | : | : | : | : | : | : | : |
| **(N=1313)** | 1: 584 (44.5%) | Mean (SD): 43.5 (10.2) | 0: 0 (0%) | 0: 838 (63.8%) | 0: 1156 (88.0%) | 0: 1134 (86.4%) | Mean (SD): 149 (69.7) | Mean (SD): 187 (28.3) | Mean (SD): 35.7 (7.35) | Mean (SD): 0.634 (0.0905) | 0: 276 (21.0%) | 0: 3 (0.2%) |
|  | 2: 729 (55.5%) | Median [Min, Max]: 43.0 [25.0, 64.0] | 1: 0 (0%) | 1: 474 (36.1%) | 1: 157 (12.0%) | 1: 179 (13.6%) | Median [Min, Max]: 123 [84.4, 512] | Median [Min, Max]: 181 [140, 298] | Median [Min, Max]: 35.1 [14.3, 72.4] | Median [Min, Max]: 0.630 [0.232, 1.12] | 1: 253 (19.3%) | 1: 65 (5.0%) |
|  |  |  | Missing: 1313 (100%) | Missing: 1 (0.1%) |  |  |  |  |  |  | 2: 784 (59.7%) | 2: 204 (15.5%) |
|  |  |  |  |  |  |  |  |  |  |  |  | 3: 1041 (79.3%) |
| **Armenia** | : | : | : | : | : | : | : | : | : | : | : | : |
| **(N=1223)** | 1: 318 (26.0%) | Mean (SD): 45.5 (11.6) | 0: 456 (37.3%) | 0: 1025 (83.8%) | 0: 1141 (93.3%) | 0: 995 (81.4%) | Mean (SD): 85.1 (32.9) | Mean (SD): 175 (44.4) | Mean (SD): 27.6 (6.27) | Mean (SD): 0.580 (0.0967) | 0: 865 (70.7%) | 0: 38 (3.1%) |
|  | 2: 905 (74.0%) | Median [Min, Max]: 47.0 [25.0, 64.0] | 1: 767 (62.7%) | 1: 198 (16.2%) | 1: 82 (6.7%) | 1: 228 (18.6%) | Median [Min, Max]: 79.2 [36.0, 401] | Median [Min, Max]: 172 [100, 401] | Median [Min, Max]: 26.7 [15.1, 57.9] | Median [Min, Max]: 0.572 [0.261, 1.07] | 1: 73 (6.0%) | 1: 446 (36.5%) |
|  |  |  | Missing: 0 (0%) | Missing: 0 (0%) |  |  |  |  |  |  | 2: 285 (23.3%) | 2: 376 (30.7%) |
|  |  |  |  |  |  |  |  |  |  |  |  | 3: 363 (29.7%) |
| **Azerbaijan** | : | : | : | : | : | : | : | : | : | : | : | : |
| **(N=2068)** | 1: 850 (41.1%) | Mean (SD): 46.0 (11.2) | 0: 895 (43.3%) | 0: 1626 (78.6%) | 0: 1926 (93.1%) | 0: 1501 (72.6%) | Mean (SD): 95.3 (38.2) | Mean (SD): 177 (44.5) | Mean (SD): 27.8 (5.24) | Mean (SD): 0.566 (0.0887) | 0: 1482 (71.7%) | 0: 34 (1.6%) |
|  | 2: 1218 (58.9%) | Median [Min, Max]: 47.0 [25.0, 64.0] | 1: 1173 (56.7%) | 1: 442 (21.4%) | 1: 142 (6.9%) | 1: 567 (27.4%) | Median [Min, Max]: 86.4 [36.0, 540] | Median [Min, Max]: 174 [100, 401] | Median [Min, Max]: 27.2 [14.8, 65.8] | Median [Min, Max]: 0.562 [0.327, 1.04] | 1: 139 (6.7%) | 1: 626 (30.3%) |
|  |  |  | Missing: 0 (0%) | Missing: 0 (0%) |  |  |  |  |  |  | 2: 447 (21.6%) | 2: 820 (39.7%) |
|  |  |  |  |  |  |  |  |  |  |  |  | 3: 588 (28.4%) |
| **Bangladesh** | : | : | : | : | : | : | : | : | : | : | : | : |
| **(N=5950)** | 1: 2786 (46.8%) | Mean (SD): 41.1 (10.0) | 0: 3124 (52.5%) | 0: 4476 (75.2%) | 0: 5532 (93.0%) | 0: 4815 (80.9%) | Mean (SD): 101 (36.2) | Mean (SD): 174 (38.9) | Mean (SD): 23.2 (4.46) | Mean (SD): 0.516 (0.0726) | 0: 5532 (93.0%) | 0: 760 (12.8%) |
|  | 2: 3164 (53.2%) | Median [Min, Max]: 40.0 [25.0, 64.0] | 1: 2826 (47.5%) | 1: 1474 (24.8%) | 1: 418 (7.0%) | 1: 1135 (19.1%) | Median [Min, Max]: 93.0 [41.0, 466] | Median [Min, Max]: 171 [74.0, 650] | Median [Min, Max]: 22.8 [10.4, 64.7] | Median [Min, Max]: 0.513 [0.220, 1.17] | 1: 108 (1.8%) | 1: 3331 (56.0%) |
|  |  |  | Missing: 0 (0%) | Missing: 0 (0%) |  |  |  |  |  |  | 2: 310 (5.2%) | 2: 1436 (24.1%) |
|  |  |  |  |  |  |  |  |  |  |  |  | 3: 423 (7.1%) |
| **Belarus** | : | : | : | : | : | : | : | : | : | : | : | : |
| **(N=3955)** | 1: 1676 (42.4%) | Mean (SD): 46.5 (10.9) | 0: 0 (0%) | 0: 2809 (71.0%) | 0: 3719 (94.0%) | 0: 2361 (59.7%) | Mean (SD): 86.5 (23.5) | Mean (SD): 189 (39.9) | Mean (SD): 27.7 (5.42) | Mean (SD): 0.542 (0.0875) | 0: 2783 (70.4%) | 0: 53 (1.3%) |
|  | 2: 2279 (57.6%) | Median [Min, Max]: 47.0 [25.0, 64.0] | 1: 0 (0%) | 1: 1146 (29.0%) | 1: 236 (6.0%) | 1: 1594 (40.3%) | Median [Min, Max]: 82.8 [37.8, 463] | Median [Min, Max]: 186 [100, 396] | Median [Min, Max]: 27.1 [15.1, 72.7] | Median [Min, Max]: 0.534 [0.331, 0.915] | 1: 154 (3.9%) | 1: 1276 (32.3%) |
|  |  |  | Missing: 3955 (100%) | Missing: 0 (0%) |  |  |  |  |  |  | 2: 1018 (25.7%) | 2: 1436 (36.3%) |
|  |  |  |  |  |  |  |  |  |  |  |  | 3: 1190 (30.1%) |
| **Benin** | : | : | : | : | : | : | : | : | : | : | : | : |
| **(N=3790)** | 1: 1805 (47.6%) | Mean (SD): 39.6 (10.3) | 0: 1950 (51.5%) | 0: 3565 (94.1%) | 0: 3763 (99.3%) | 0: 3539 (93.4%) | Mean (SD): 89.6 (28.3) | Mean (SD): 156 (46.3) | Mean (SD): 23.9 (4.90) | Mean (SD): 0.509 (0.0715) | 0: 3393 (89.5%) | 0: 308 (8.1%) |
|  | 2: 1985 (52.4%) | Median [Min, Max]: 38.0 [25.0, 64.0] | 1: 1840 (48.5%) | 1: 225 (5.9%) | 1: 27 (0.7%) | 1: 251 (6.6%) | Median [Min, Max]: 86.0 [41.0, 390] | Median [Min, Max]: 147 [74.0, 450] | Median [Min, Max]: 22.9 [11.4, 59.9] | Median [Min, Max]: 0.497 [0.315, 0.938] | 1: 103 (2.7%) | 1: 2296 (60.6%) |
|  |  |  | Missing: 0 (0%) | Missing: 0 (0%) |  |  |  |  |  |  | 2: 294 (7.8%) | 2: 776 (20.5%) |
|  |  |  |  |  |  |  |  |  |  |  |  | 3: 410 (10.8%) |
| **Bhutan** | : | : | : | : | : | : | : | : | : | : | : | : |
| **(N=4459)** | 1: 1726 (38.7%) | Mean (SD): 41.3 (10.7) | 0: 2834 (63.6%) | 0: 4193 (94.0%) | 0: 4284 (96.1%) | 0: 3539 (79.4%) | Mean (SD): 83.3 (20.4) | Mean (SD): 147 (38.4) | Mean (SD): 25.9 (4.41) | Mean (SD): 0.544 (0.0724) | 0: 3779 (84.8%) | 0: 95 (2.1%) |
|  | 2: 2733 (61.3%) | Median [Min, Max]: 40.0 [25.0, 64.0] | 1: 1625 (36.4%) | 1: 266 (6.0%) | 1: 175 (3.9%) | 1: 920 (20.6%) | Median [Min, Max]: 81.0 [36.0, 506] | Median [Min, Max]: 141 [77.0, 400] | Median [Min, Max]: 25.5 [10.0, 66.2] | Median [Min, Max]: 0.541 [0.228, 1.16] | 1: 279 (6.3%) | 1: 1913 (42.9%) |
|  |  |  | Missing: 0 (0%) | Missing: 0 (0%) |  |  |  |  |  |  | 2: 401 (9.0%) | 2: 1768 (39.7%) |
|  |  |  |  |  |  |  |  |  |  |  |  | 3: 683 (15.3%) |
| **Botswana** | : | : | : | : | : | : | : | : | : | : | : | : |
| **(N=2398)** | 1: 750 (31.3%) | Mean (SD): 40.8 (11.2) | 0: 0 (0%) | 0: 2062 (86.0%) | 0: 2324 (96.9%) | 0: 1622 (67.6%) | Mean (SD): 82.9 (28.5) | Mean (SD): 150 (43.7) | Mean (SD): 25.4 (6.33) | Mean (SD): 0.527 (0.100) | 0: 1923 (80.2%) | 0: 225 (9.4%) |
|  | 2: 1648 (68.7%) | Median [Min, Max]: 39.0 [25.0, 64.0] | 1: 0 (0%) | 1: 336 (14.0%) | 1: 74 (3.1%) | 1: 776 (32.4%) | Median [Min, Max]: 79.2 [37.8, 540] | Median [Min, Max]: 143 [100, 401] | Median [Min, Max]: 24.3 [12.2, 64.4] | Median [Min, Max]: 0.518 [0.200, 1.00] | 1: 126 (5.3%) | 1: 1072 (44.7%) |
|  |  |  | Missing: 2398 (100%) | Missing: 0 (0%) |  |  |  |  |  |  | 2: 349 (14.6%) | 2: 606 (25.3%) |
|  |  |  |  |  |  |  |  |  |  |  |  | 3: 495 (20.6%) |
| **Brunei Darussalam** | : | : | : | : | : | : | : | : | : | : | : | : |
| **(N=1405)** | 1: 582 (41.4%) | Mean (SD): 43.8 (11.3) | 0: 0 (0%) | 0: 1190 (84.7%) | 0: 1205 (85.8%) | 0: 923 (65.7%) | Mean (SD): 99.2 (38.1) | Mean (SD): 200 (40.2) | Mean (SD): 28.2 (5.44) | Mean (SD): 0.568 (0.0833) | 0: 959 (68.3%) | 0: 26 (1.9%) |
|  | 2: 823 (58.6%) | Median [Min, Max]: 43.0 [25.0, 64.0] | 1: 0 (0%) | 1: 215 (15.3%) | 1: 200 (14.2%) | 1: 482 (34.3%) | Median [Min, Max]: 88.2 [43.2, 392] | Median [Min, Max]: 196 [79.7, 364] | Median [Min, Max]: 27.6 [13.2, 54.1] | Median [Min, Max]: 0.564 [0.333, 1.27] | 1: 77 (5.5%) | 1: 365 (26.0%) |
|  |  |  | Missing: 1405 (100%) | Missing: 0 (0%) |  |  |  |  |  |  | 2: 369 (26.3%) | 2: 565 (40.2%) |
|  |  |  |  |  |  |  |  |  |  |  |  | 3: 449 (32.0%) |
| **Cabo Verde** | : | : | : | : | : | : | : | : | : | : | : | : |
| **(N=1783)** | 1: 660 (37.0%) | Mean (SD): 43.6 (11.3) | 0: 751 (42.1%) | 0: 1654 (92.8%) | 0: 1683 (94.4%) | 0: 1292 (72.5%) | Mean (SD): 87.7 (30.6) | Mean (SD): 165 (42.8) | Mean (SD): 26.3 (5.20) | Mean (SD): 0.516 (0.0805) | 0: 1430 (80.2%) | 0: 43 (2.4%) |
|  | 2: 1123 (63.0%) | Median [Min, Max]: 43.0 [25.0, 64.0] | 1: 1032 (57.9%) | 1: 129 (7.2%) | 1: 100 (5.6%) | 1: 491 (27.5%) | Median [Min, Max]: 82.0 [36.0, 427] | Median [Min, Max]: 161 [100, 400] | Median [Min, Max]: 25.7 [15.2, 63.1] | Median [Min, Max]: 0.508 [0.306, 0.969] | 1: 111 (6.2%) | 1: 736 (41.3%) |
|  |  |  | Missing: 0 (0%) | Missing: 0 (0%) |  |  |  |  |  |  | 2: 242 (13.6%) | 2: 643 (36.1%) |
|  |  |  |  |  |  |  |  |  |  |  |  | 3: 361 (20.2%) |
| **Cambodia** | : | : | : | : | : | : | : | : | : | : | : | : |
| **(N=5011)** | 1: 1786 (35.6%) | Mean (SD): 43.4 (11.0) | 0: 4123 (82.3%) | 0: 3807 (76.0%) | 0: 4899 (97.8%) | 0: 4445 (88.7%) | Mean (SD): 79.8 (25.2) | Mean (SD): 175 (27.2) | Mean (SD): 21.9 (3.49) | Mean (SD): 0.491 (0.0618) | 0: 4893 (97.6%) | 0: 681 (13.6%) |
|  | 2: 3225 (64.4%) | Median [Min, Max]: 43.0 [25.0, 64.0] | 1: 888 (17.7%) | 1: 1204 (24.0%) | 1: 112 (2.2%) | 1: 566 (11.3%) | Median [Min, Max]: 75.5 [36.6, 420] | Median [Min, Max]: 167 [150, 300] | Median [Min, Max]: 21.4 [10.9, 42.0] | Median [Min, Max]: 0.481 [0.279, 0.810] | 1: 44 (0.9%) | 1: 3450 (68.8%) |
|  |  |  | Missing: 0 (0%) | Missing: 0 (0%) |  |  |  |  |  |  | 2: 74 (1.5%) | 2: 761 (15.2%) |
|  |  |  |  |  |  |  |  |  |  |  |  | 3: 119 (2.4%) |
| **Comoros** | : | : | : | : | : | : | : | : | : | : | : | : |
| **(N=1423)** | 1: 327 (23.0%) | Mean (SD): 42.4 (11.5) | 0: 934 (65.6%) | 0: 1332 (93.6%) | 0: 1351 (94.9%) | 0: 1183 (83.1%) | Mean (SD): 74.2 (34.1) | Mean (SD): 179 (27.7) | Mean (SD): 26.8 (6.09) | Mean (SD): 0.573 (0.0961) | 0: 1061 (74.6%) | 0: 49 (3.4%) |
|  | 2: 1096 (77.0%) | Median [Min, Max]: 41.0 [25.0, 64.0] | 1: 489 (34.4%) | 1: 91 (6.4%) | 1: 72 (5.1%) | 1: 240 (16.9%) | Median [Min, Max]: 68.0 [36.0, 467] | Median [Min, Max]: 170 [117, 300] | Median [Min, Max]: 25.9 [11.3, 67.6] | Median [Min, Max]: 0.564 [0.221, 1.32] | 1: 122 (8.6%) | 1: 584 (41.0%) |
|  |  |  | Missing: 0 (0%) | Missing: 0 (0%) |  |  |  |  |  |  | 2: 240 (16.9%) | 2: 424 (29.8%) |
|  |  |  |  |  |  |  |  |  |  |  |  | 3: 366 (25.7%) |
| **Cook Islands** | : | : | : | : | : | : | : | : | : | : | : | : |
| **(N=616)** | 1: 282 (45.8%) | Mean (SD): 44.4 (10.8) | 0: 0 (0%) | 0: 447 (72.6%) | 0: 532 (86.4%) | 0: 479 (77.8%) | Mean (SD): 132 (42.5) | Mean (SD): 198 (33.4) | Mean (SD): 34.4 (7.92) | Mean (SD): 0.625 (0.112) | 0: 195 (31.7%) | 0: 2 (0.3%) |
|  | 2: 334 (54.2%) | Median [Min, Max]: 45.0 [25.0, 64.0] | 1: 0 (0%) | 1: 169 (27.4%) | 1: 84 (13.6%) | 1: 137 (22.2%) | Median [Min, Max]: 120 [63.9, 440] | Median [Min, Max]: 193 [150, 300] | Median [Min, Max]: 33.7 [12.0, 75.5] | Median [Min, Max]: 0.618 [0.223, 1.14] | 1: 78 (12.7%) | 1: 57 (9.3%) |
|  |  |  | Missing: 616 (100%) | Missing: 0 (0%) |  |  |  |  |  |  | 2: 343 (55.7%) | 2: 132 (21.4%) |
|  |  |  |  |  |  |  |  |  |  |  |  | 3: 425 (69.0%) |
| **Ecuador** | : | : | : | : | : | : | : | : | : | : | : | : |
| **(N=3083)** | 1: 1267 (41.1%) | Mean (SD): 43.0 (11.1) | 0: 0 (0%) | 0: 2682 (87.0%) | 0: 2857 (92.7%) | 0: 2494 (80.9%) | Mean (SD): 96.4 (38.2) | Mean (SD): 178 (48.4) | Mean (SD): 28.0 (4.90) | Mean (SD): 0.581 (0.0754) | 0: 2180 (70.7%) | 0: 27 (0.9%) |
|  | 2: 1816 (58.9%) | Median [Min, Max]: 42.0 [25.0, 64.0] | 1: 0 (0%) | 1: 401 (13.0%) | 1: 226 (7.3%) | 1: 589 (19.1%) | Median [Min, Max]: 90.0 [36.0, 439] | Median [Min, Max]: 176 [94.0, 386] | Median [Min, Max]: 27.5 [15.2, 70.3] | Median [Min, Max]: 0.576 [0.359, 0.953] | 1: 298 (9.7%) | 1: 853 (27.7%) |
|  |  |  | Missing: 3083 (100%) | Missing: 0 (0%) |  |  |  |  |  |  | 2: 605 (19.6%) | 2: 1296 (42.0%) |
|  |  |  |  |  |  |  |  |  |  |  |  | 3: 907 (29.4%) |
| **Eritrea** | : | : | : | : | : | : | : | : | : | : | : | : |
| **(N=4350)** | 1: 1094 (25.1%) | Mean (SD): 41.7 (11.2) | 0: 0 (0%) | 0: 4192 (96.4%) | 0: 4261 (98.0%) | 0: 4061 (93.4%) | Mean (SD): 80.3 (28.5) | Mean (SD): 185 (31.5) | Mean (SD): 20.3 (3.89) | Mean (SD): 0.473 (0.0692) | 0: 4252 (97.7%) | 0: 1587 (36.5%) |
|  | 2: 3256 (74.9%) | Median [Min, Max]: 40.0 [25.0, 64.0] | 1: 0 (0%) | 1: 158 (3.6%) | 1: 89 (2.0%) | 1: 289 (6.6%) | Median [Min, Max]: 76.6 [36.6, 481] | Median [Min, Max]: 176 [150, 300] | Median [Min, Max]: 19.6 [11.2, 42.4] | Median [Min, Max]: 0.464 [0.231, 1.23] | 1: 37 (0.9%) | 1: 2248 (51.7%) |
|  |  |  | Missing: 4350 (100%) | Missing: 0 (0%) |  |  |  |  |  |  | 2: 61 (1.4%) | 2: 412 (9.5%) |
|  |  |  |  |  |  |  |  |  |  |  |  | 3: 103 (2.4%) |
| **Eswatini** | : | : | : | : | : | : | : | : | : | : | : | : |
| **(N=1645)** | 1: 558 (33.9%) | Mean (SD): 41.8 (11.3) | 0: 0 (0%) | 0: 1524 (92.6%) | 0: 1588 (96.5%) | 0: 1270 (77.2%) | Mean (SD): 94.5 (26.4) | Mean (SD): 154 (40.8) | Mean (SD): 27.9 (6.86) | Mean (SD): 0.553 (0.0995) | 0: 1129 (68.6%) | 0: 51 (3.1%) |
|  | 2: 1087 (66.1%) | Median [Min, Max]: 40.0 [25.0, 64.0] | 1: 0 (0%) | 1: 121 (7.4%) | 1: 57 (3.5%) | 1: 375 (22.8%) | Median [Min, Max]: 91.8 [36.0, 398] | Median [Min, Max]: 148 [100, 401] | Median [Min, Max]: 26.6 [12.0, 79.4] | Median [Min, Max]: 0.539 [0.250, 1.33] | 1: 163 (9.9%) | 1: 600 (36.5%) |
|  |  |  | Missing: 1645 (100%) | Missing: 0 (0%) |  |  |  |  |  |  | 2: 353 (21.5%) | 2: 468 (28.4%) |
|  |  |  |  |  |  |  |  |  |  |  |  | 3: 526 (32.0%) |
| **Ethiopia** | : | : | : | : | : | : | : | : | : | : | : | : |
| **(N=6181)** | 1: 2625 (42.5%) | Mean (SD): 38.2 (10.3) | 0: 4543 (73.5%) | 0: 5806 (93.9%) | 0: 6107 (98.8%) | 0: 5805 (93.9%) | Mean (SD): 82.8 (22.7) | Mean (SD): 141 (35.7) | Mean (SD): 21.1 (3.67) | Mean (SD): 0.473 (0.0683) | 0: 6019 (97.4%) | 0: 1372 (22.2%) |
|  | 2: 3556 (57.5%) | Median [Min, Max]: 36.0 [25.0, 64.0] | 1: 1638 (26.5%) | 1: 375 (6.1%) | 1: 74 (1.2%) | 1: 376 (6.1%) | Median [Min, Max]: 79.0 [37.0, 521] | Median [Min, Max]: 134 [100, 400] | Median [Min, Max]: 20.4 [10.6, 51.4] | Median [Min, Max]: 0.462 [0.255, 1.25] | 1: 52 (0.8%) | 1: 4092 (66.2%) |
|  |  |  | Missing: 0 (0%) | Missing: 0 (0%) |  |  |  |  |  |  | 2: 110 (1.8%) | 2: 543 (8.8%) |
|  |  |  |  |  |  |  |  |  |  |  |  | 3: 174 (2.8%) |
| **French Polynesia** | : | : | : | : | : | : | : | : | : | : | : | : |
| **(N=1723)** | 1: 736 (42.7%) | Mean (SD): 43.4 (10.6) | 0: 0 (0%) | 0: 1087 (63.1%) | 0: 1534 (89.0%) | 0: 1475 (85.6%) | Mean (SD): 87.7 (26.5) | Mean (SD): 197 (32.6) | Mean (SD): 30.1 (6.82) | Mean (SD): 0.579 (0.101) | 0: 949 (55.1%) | 0: 24 (1.4%) |
|  | 2: 987 (57.3%) | Median [Min, Max]: 43.0 [25.0, 64.0] | 1: 0 (0%) | 1: 636 (36.9%) | 1: 189 (11.0%) | 1: 248 (14.4%) | Median [Min, Max]: 85.5 [36.6, 331] | Median [Min, Max]: 192 [125, 298] | Median [Min, Max]: 29.2 [16.6, 65.2] | Median [Min, Max]: 0.571 [0.202, 1.20] | 1: 211 (12.2%) | 1: 405 (23.5%) |
|  |  |  | Missing: 1723 (100%) | Missing: 0 (0%) |  |  |  |  |  |  | 2: 563 (32.7%) | 2: 509 (29.5%) |
|  |  |  |  |  |  |  |  |  |  |  |  | 3: 785 (45.6%) |
| **Georgia** | : | : | : | : | : | : | : | : | : | : | : | : |
| **(N=2452)** | 1: 667 (27.2%) | Mean (SD): 47.9 (11.0) | 0: 0 (0%) | 0: 1938 (79.0%) | 0: 2277 (92.9%) | 0: 1578 (64.4%) | Mean (SD): 83.6 (33.4) | Mean (SD): 178 (49.7) | Mean (SD): 29.5 (6.76) | Mean (SD): 0.577 (0.103) | 0: 1436 (58.6%) | 0: 46 (1.9%) |
|  | 2: 1785 (72.8%) | Median [Min, Max]: 50.0 [25.0, 64.0] | 1: 0 (0%) | 1: 514 (21.0%) | 1: 175 (7.1%) | 1: 874 (35.6%) | Median [Min, Max]: 75.6 [36.0, 488] | Median [Min, Max]: 173 [100, 401] | Median [Min, Max]: 28.7 [13.7, 65.3] | Median [Min, Max]: 0.573 [0.242, 1.16] | 1: 176 (7.2%) | 1: 619 (25.2%) |
|  |  |  | Missing: 2452 (100%) | Missing: 0 (0%) |  |  |  |  |  |  | 2: 840 (34.3%) | 2: 759 (31.0%) |
|  |  |  |  |  |  |  |  |  |  |  |  | 3: 1028 (41.9%) |
| **Iraq** | : | : | : | : | : | : | : | : | : | : | : | : |
| **(N=793)** | 1: 280 (35.3%) | Mean (SD): 41.3 (10.6) | 0: 0 (0%) | 0: 678 (85.5%) | 0: 707 (89.2%) | 0: 573 (72.3%) | Mean (SD): 99.1 (42.6) | Mean (SD): 178 (45.7) | Mean (SD): 30.2 (6.98) | Mean (SD): 0.622 (0.111) | 0: 443 (55.9%) | 0: 11 (1.4%) |
|  | 2: 513 (64.7%) | Median [Min, Max]: 40.0 [25.0, 64.0] | 1: 0 (0%) | 1: 115 (14.5%) | 1: 86 (10.8%) | 1: 220 (27.7%) | Median [Min, Max]: 90.0 [42.0, 507] | Median [Min, Max]: 175 [70.0, 356] | Median [Min, Max]: 29.4 [16.9, 75.6] | Median [Min, Max]: 0.616 [0.261, 1.29] | 1: 92 (11.6%) | 1: 152 (19.2%) |
|  |  |  | Missing: 793 (100%) | Missing: 0 (0%) |  |  |  |  |  |  | 2: 258 (32.5%) | 2: 276 (34.8%) |
|  |  |  |  |  |  |  |  |  |  |  |  | 3: 354 (44.6%) |
| **Kenya** | : | : | : | : | : | : | : | : | : | : | : | : |
| **(N=3076)** | 1: 1254 (40.8%) | Mean (SD): 40.0 (10.8) | 0: 1396 (45.4%) | 0: 2792 (90.8%) | 0: 3021 (98.2%) | 0: 2718 (88.4%) | Mean (SD): 85.1 (24.0) | Mean (SD): 145 (39.5) | Mean (SD): 23.9 (5.33) | Mean (SD): 0.497 (0.0835) | 0: 2720 (88.4%) | 0: 336 (10.9%) |
|  | 2: 1822 (59.2%) | Median [Min, Max]: 38.0 [25.0, 64.0] | 1: 1680 (54.6%) | 1: 284 (9.2%) | 1: 55 (1.8%) | 1: 358 (11.6%) | Median [Min, Max]: 82.8 [36.0, 432] | Median [Min, Max]: 139 [99.4, 398] | Median [Min, Max]: 22.9 [10.0, 75.2] | Median [Min, Max]: 0.488 [0.201, 0.982] | 1: 141 (4.6%) | 1: 1664 (54.1%) |
|  |  |  | Missing: 0 (0%) | Missing: 0 (0%) |  |  |  |  |  |  | 2: 215 (7.0%) | 2: 702 (22.8%) |
|  |  |  |  |  |  |  |  |  |  |  |  | 3: 374 (12.2%) |
| **Kiribati** | : | : | : | : | : | : | : | : | : | : | : | : |
| **(N=896)** | 1: 398 (44.4%) | Mean (SD): 41.4 (10.9) | 0: 0 (0%) | 0: 452 (50.4%) | 0: 828 (92.4%) | 0: 770 (85.9%) | Mean (SD): 111 (44.8) | Mean (SD): 159 (56.6) | Mean (SD): 30.3 (6.46) | Mean (SD): 0.569 (0.0896) | 0: 479 (53.5%) | 0: 9 (1.0%) |
|  | 2: 498 (55.6%) | Median [Min, Max]: 41.0 [25.0, 64.0] | 1: 0 (0%) | 1: 444 (49.6%) | 1: 68 (7.6%) | 1: 126 (14.1%) | Median [Min, Max]: 103 [36.0, 410] | Median [Min, Max]: 150 [100, 502] | Median [Min, Max]: 29.7 [12.5, 78.5] | Median [Min, Max]: 0.564 [0.361, 1.20] | 1: 128 (14.3%) | 1: 172 (19.2%) |
|  |  |  | Missing: 896 (100%) | Missing: 0 (0%) |  |  |  |  |  |  | 2: 289 (32.3%) | 2: 289 (32.3%) |
|  |  |  |  |  |  |  |  |  |  |  |  | 3: 426 (47.5%) |
| **Kuwait** | : | : | : | : | : | : | : | : | : | : | : | : |
| **(N=1492)** | 1: 564 (37.8%) | Mean (SD): 40.2 (10.4) | 0: 0 (0%) | 0: 1221 (81.8%) | 0: 1294 (86.7%) | 0: 1179 (79.0%) | Mean (SD): 105 (44.3) | Mean (SD): 197 (37.7) | Mean (SD): 30.5 (6.24) | Mean (SD): 0.569 (0.0890) | 0: 813 (54.5%) | 0: 3 (0.2%) |
|  | 2: 928 (62.2%) | Median [Min, Max]: 39.0 [25.0, 64.0] | 1: 0 (0%) | 1: 271 (18.2%) | 1: 198 (13.3%) | 1: 313 (21.0%) | Median [Min, Max]: 91.8 [52.2, 414] | Median [Min, Max]: 193 [112, 553] | Median [Min, Max]: 29.6 [16.3, 79.5] | Median [Min, Max]: 0.562 [0.253, 1.26] | 1: 175 (11.7%) | 1: 242 (16.2%) |
|  |  |  | Missing: 1492 (100%) | Missing: 0 (0%) |  |  |  |  |  |  | 2: 504 (33.8%) | 2: 550 (36.9%) |
|  |  |  |  |  |  |  |  |  |  |  |  | 3: 697 (46.7%) |
| **Kyrgyzstan** | : | : | : | : | : | : | : | : | : | : | : | : |
| **(N=2476)** | 1: 914 (36.9%) | Mean (SD): 44.1 (11.3) | 0: 1482 (59.9%) | 0: 2004 (80.9%) | 0: 2362 (95.4%) | 0: 1824 (73.7%) | Mean (SD): 89.4 (32.0) | Mean (SD): 172 (38.2) | Mean (SD): 27.5 (5.56) | Mean (SD): 0.558 (0.0867) | 0: 1775 (71.7%) | 0: 39 (1.6%) |
|  | 2: 1562 (63.1%) | Median [Min, Max]: 44.0 [25.0, 64.0] | 1: 994 (40.1%) | 1: 472 (19.1%) | 1: 114 (4.6%) | 1: 652 (26.3%) | Median [Min, Max]: 84.6 [36.0, 416] | Median [Min, Max]: 168 [100, 401] | Median [Min, Max]: 26.7 [15.4, 64.1] | Median [Min, Max]: 0.550 [0.358, 0.997] | 1: 124 (5.0%) | 1: 870 (35.1%) |
|  |  |  | Missing: 0 (0%) | Missing: 0 (0%) |  |  |  |  |  |  | 2: 577 (23.3%) | 2: 858 (34.7%) |
|  |  |  |  |  |  |  |  |  |  |  |  | 3: 709 (28.6%) |
| **Lao People's Democratic Republic** | : | : | : | : | : | : | : | : | : | : | : | : |
| **(N=2068)** | 1: 834 (40.3%) | Mean (SD): 42.0 (10.5) | 0: 1429 (69.1%) | 0: 1440 (69.6%) | 0: 1991 (96.3%) | 0: 1879 (90.9%) | Mean (SD): 83.1 (35.9) | Mean (SD): 166 (43.4) | Mean (SD): 23.1 (4.02) | Mean (SD): 0.511 (0.0655) | 0: 1942 (93.9%) | 0: 199 (9.6%) |
|  | 2: 1234 (59.7%) | Median [Min, Max]: 41.5 [25.0, 64.0] | 1: 639 (30.9%) | 1: 628 (30.4%) | 1: 77 (3.7%) | 1: 189 (9.1%) | Median [Min, Max]: 75.6 [36.0, 504] | Median [Min, Max]: 161 [82.4, 432] | Median [Min, Max]: 22.5 [12.2, 48.6] | Median [Min, Max]: 0.503 [0.220, 0.800] | 1: 42 (2.0%) | 1: 1314 (63.5%) |
|  |  |  | Missing: 0 (0%) | Missing: 0 (0%) |  |  |  |  |  |  | 2: 84 (4.1%) | 2: 426 (20.6%) |
|  |  |  |  |  |  |  |  |  |  |  |  | 3: 129 (6.2%) |
| **Lebanon** | : | : | : | : | : | : | : | : | : | : | : | : |
| **(N=945)** | 1: 342 (36.2%) | Mean (SD): 46.7 (10.1) | 0: 0 (0%) | 0: 595 (63.0%) | 0: 853 (90.3%) | 0: 706 (74.7%) | Mean (SD): 100 (40.5) | Mean (SD): 215 (44.9) | Mean (SD): 28.1 (5.41) | Mean (SD): 0.577 (0.101) | 0: 655 (69.3%) | 0: 12 (1.3%) |
|  | 2: 603 (63.8%) | Median [Min, Max]: 48.0 [25.0, 64.0] | 1: 0 (0%) | 1: 350 (37.0%) | 1: 92 (9.7%) | 1: 239 (25.3%) | Median [Min, Max]: 89.0 [54.0, 396] | Median [Min, Max]: 213 [71.0, 457] | Median [Min, Max]: 27.6 [14.1, 56.5] | Median [Min, Max]: 0.575 [0.244, 1.07] | 1: 42 (4.4%) | 1: 288 (30.5%) |
|  |  |  | Missing: 945 (100%) | Missing: 0 (0%) |  |  |  |  |  |  | 2: 248 (26.2%) | 2: 345 (36.5%) |
|  |  |  |  |  |  |  |  |  |  |  |  | 3: 300 (31.7%) |
| **Lesotho** | : | : | : | : | : | : | : | : | : | : | : | : |
| **(N=1877)** | 1: 630 (33.6%) | Mean (SD): 43.3 (12.1) | 0: 0 (0%) | 0: 1574 (83.9%) | 0: 1824 (97.2%) | 0: 1507 (80.3%) | Mean (SD): 82.9 (25.4) | Mean (SD): 138 (33.5) | Mean (SD): 26.4 (6.98) | Mean (SD): 0.536 (0.0943) | 0: 1431 (76.2%) | 0: 103 (5.5%) |
|  | 2: 1247 (66.4%) | Median [Min, Max]: 42.0 [25.0, 64.0] | 1: 0 (0%) | 1: 303 (16.1%) | 1: 53 (2.8%) | 1: 370 (19.7%) | Median [Min, Max]: 79.2 [36.0, 481] | Median [Min, Max]: 133 [83.1, 401] | Median [Min, Max]: 24.7 [10.3, 76.6] | Median [Min, Max]: 0.520 [0.225, 1.18] | 1: 151 (8.0%) | 1: 861 (45.9%) |
|  |  |  | Missing: 1877 (100%) | Missing: 0 (0%) |  |  |  |  |  |  | 2: 295 (15.7%) | 2: 459 (24.5%) |
|  |  |  |  |  |  |  |  |  |  |  |  | 3: 454 (24.2%) |
| **Libya** | : | : | : | : | : | : | : | : | : | : | : | : |
| **(N=1661)** | 1: 927 (55.8%) | Mean (SD): 41.7 (10.3) | 0: 0 (0%) | 0: 1259 (75.8%) | 0: 1495 (90.0%) | 0: 1449 (87.2%) | Mean (SD): 106 (42.0) | Mean (SD): 177 (26.9) | Mean (SD): 28.1 (5.82) | Mean (SD): 0.576 (0.111) | 0: 1140 (68.6%) | 0: 41 (2.5%) |
|  | 2: 734 (44.2%) | Median [Min, Max]: 41.0 [25.0, 64.0] | 1: 0 (0%) | 1: 402 (24.2%) | 1: 166 (10.0%) | 1: 212 (12.8%) | Median [Min, Max]: 97.9 [38.0, 402] | Median [Min, Max]: 170 [139, 300] | Median [Min, Max]: 27.5 [10.0, 67.5] | Median [Min, Max]: 0.576 [0.200, 1.24] | 1: 167 (10.1%) | 1: 475 (28.6%) |
|  |  |  | Missing: 1661 (100%) | Missing: 0 (0%) |  |  |  |  |  |  | 2: 354 (21.3%) | 2: 607 (36.5%) |
|  |  |  |  |  |  |  |  |  |  |  |  | 3: 538 (32.4%) |
| **Malawi** | : | : | : | : | : | : | : | : | : | : | : | : |
| **(N=2777)** | 1: 1011 (36.4%) | Mean (SD): 40.7 (10.7) | 0: 2241 (80.7%) | 0: 2502 (90.1%) | 0: 2758 (99.3%) | 0: 2441 (87.9%) | Mean (SD): 86.4 (19.7) | Mean (SD): 147 (37.8) | Mean (SD): 23.5 (4.68) | Mean (SD): 0.496 (0.0682) | 0: 2525 (90.9%) | 0: 186 (6.7%) |
|  | 2: 1766 (63.6%) | Median [Min, Max]: 39.0 [25.0, 64.0] | 1: 536 (19.3%) | 1: 275 (9.9%) | 1: 19 (0.7%) | 1: 336 (12.1%) | Median [Min, Max]: 84.6 [36.0, 331] | Median [Min, Max]: 139 [96.7, 333] | Median [Min, Max]: 22.5 [12.5, 70.7] | Median [Min, Max]: 0.484 [0.314, 0.921] | 1: 105 (3.8%) | 1: 1852 (66.7%) |
|  |  |  | Missing: 0 (0%) | Missing: 0 (0%) |  |  |  |  |  |  | 2: 147 (5.3%) | 2: 479 (17.2%) |
|  |  |  |  |  |  |  |  |  |  |  |  | 3: 260 (9.4%) |
| **Mongolia** | : | : | : | : | : | : | : | : | : | : | : | : |
| **(N=5054)** | 1: 2279 (45.1%) | Mean (SD): 43.0 (10.9) | 0: 1773 (35.1%) | 0: 3698 (73.2%) | 0: 4788 (94.7%) | 0: 3403 (67.3%) | Mean (SD): 106 (26.9) | Mean (SD): 176 (39.4) | Mean (SD): 26.9 (5.03) | Mean (SD): 0.556 (0.0846) | 0: 3840 (76.0%) | 0: 86 (1.7%) |
|  | 2: 2775 (54.9%) | Median [Min, Max]: 42.0 [25.0, 64.0] | 1: 3281 (64.9%) | 1: 1356 (26.8%) | 1: 266 (5.3%) | 1: 1651 (32.7%) | Median [Min, Max]: 101 [54.0, 455] | Median [Min, Max]: 174 [96.7, 437] | Median [Min, Max]: 26.4 [10.0, 70.3] | Median [Min, Max]: 0.550 [0.203, 1.17] | 1: 271 (5.4%) | 1: 1853 (36.7%) |
|  |  |  | Missing: 0 (0%) | Missing: 0 (0%) |  |  |  |  |  |  | 2: 943 (18.7%) | 2: 1889 (37.4%) |
|  |  |  |  |  |  |  |  |  |  |  |  | 3: 1226 (24.3%) |
| **Morocco** | : | : | : | : | : | : | : | : | : | : | : | : |
| **(N=3457)** | 1: 1146 (33.2%) | Mean (SD): 44.3 (10.9) | 0: 1318 (38.1%) | 0: 3158 (91.4%) | 0: 3154 (91.2%) | 0: 2935 (84.9%) | Mean (SD): 104 (38.0) | Mean (SD): 145 (39.0) | Mean (SD): 27.3 (5.42) | Mean (SD): 0.584 (0.0860) | 0: 2513 (72.7%) | 0: 92 (2.7%) |
|  | 2: 2311 (66.8%) | Median [Min, Max]: 44.0 [25.0, 64.0] | 1: 2139 (61.9%) | 1: 299 (8.6%) | 1: 303 (8.8%) | 1: 522 (15.1%) | Median [Min, Max]: 95.0 [67.0, 525] | Median [Min, Max]: 141 [100, 400] | Median [Min, Max]: 26.8 [13.8, 67.3] | Median [Min, Max]: 0.582 [0.359, 0.933] | 1: 398 (11.5%) | 1: 1139 (32.9%) |
|  |  |  | Missing: 0 (0%) | Missing: 0 (0%) |  |  |  |  |  |  | 2: 546 (15.8%) | 2: 1280 (37.0%) |
|  |  |  |  |  |  |  |  |  |  |  |  | 3: 946 (27.4%) |
| **Myanmar** | : | : | : | : | : | : | : | : | : | : | : | : |
| **(N=7629)** | 1: 2629 (34.5%) | Mean (SD): 45.2 (10.7) | 0: 0 (0%) | 0: 5918 (77.6%) | 0: 7175 (94.0%) | 0: 5550 (72.7%) | Mean (SD): 95.1 (30.2) | Mean (SD): 182 (40.3) | Mean (SD): 22.8 (4.77) | Mean (SD): 0.501 (0.0765) | 0: 7139 (93.6%) | 0: 1283 (16.8%) |
|  | 2: 5000 (65.5%) | Median [Min, Max]: 46.0 [25.0, 64.0] | 1: 0 (0%) | 1: 1711 (22.4%) | 1: 454 (6.0%) | 1: 2079 (27.3%) | Median [Min, Max]: 88.0 [40.0, 408] | Median [Min, Max]: 180 [100, 450] | Median [Min, Max]: 22.1 [11.1, 72.9] | Median [Min, Max]: 0.493 [0.239, 1.28] | 1: 76 (1.0%) | 1: 4246 (55.7%) |
|  |  |  | Missing: 7629 (100%) | Missing: 0 (0%) |  |  |  |  |  |  | 2: 414 (5.4%) | 2: 1597 (20.9%) |
|  |  |  |  |  |  |  |  |  |  |  |  | 3: 503 (6.6%) |
| **Nauru** | : | : | : | : | : | : | : | : | : | : | : | : |
| **(N=740)** | 1: 345 (46.6%) | Mean (SD): 39.3 (10.6) | 0: 0 (0%) | 0: 410 (55.4%) | 0: 605 (81.8%) | 0: 563 (76.1%) | Mean (SD): 99.8 (57.3) | Mean (SD): 154 (53.5) | Mean (SD): 35.9 (8.15) | Mean (SD): 0.658 (0.132) | 0: 180 (24.3%) | 0: 0 (0%) |
|  | 2: 395 (53.4%) | Median [Min, Max]: 37.0 [25.0, 64.0] | 1: 0 (0%) | 1: 330 (44.6%) | 1: 135 (18.2%) | 1: 177 (23.9%) | Median [Min, Max]: 84.6 [36.0, 459] | Median [Min, Max]: 145 [100, 401] | Median [Min, Max]: 34.9 [18.7, 76.5] | Median [Min, Max]: 0.639 [0.315, 1.40] | 1: 211 (28.5%) | 1: 50 (6.8%) |
|  |  |  | Missing: 740 (100%) | Missing: 0 (0%) |  |  |  |  |  |  | 2: 349 (47.2%) | 2: 125 (16.9%) |
|  |  |  |  |  |  |  |  |  |  |  |  | 3: 565 (76.4%) |
| **Nepal** | : | : | : | : | : | : | : | : | : | : | : | : |
| **(N=4078)** | 1: 1442 (35.4%) | Mean (SD): 42.0 (11.0) | 0: 3544 (86.9%) | 0: 3260 (79.9%) | 0: 3971 (97.4%) | 0: 3676 (90.1%) | Mean (SD): 95.1 (28.8) | Mean (SD): 147 (38.7) | Mean (SD): 23.6 (4.40) | Mean (SD): 0.530 (0.0756) | 0: 3810 (93.4%) | 0: 311 (7.6%) |
|  | 2: 2636 (64.6%) | Median [Min, Max]: 40.0 [25.0, 64.0] | 1: 534 (13.1%) | 1: 818 (20.1%) | 1: 107 (2.6%) | 1: 402 (9.9%) | Median [Min, Max]: 91.0 [36.0, 524] | Median [Min, Max]: 141 [100, 400] | Median [Min, Max]: 23.0 [11.3, 69.9] | Median [Min, Max]: 0.523 [0.205, 1.22] | 1: 90 (2.2%) | 1: 2520 (61.8%) |
|  |  |  | Missing: 0 (0%) | Missing: 0 (0%) |  |  |  |  |  |  | 2: 178 (4.4%) | 2: 972 (23.8%) |
|  |  |  |  |  |  |  |  |  |  |  |  | 3: 275 (6.7%) |
| **Niue** | : | : | : | : | : | : | : | : | : | : | : | : |
| **(N=510)** | 1: 231 (45.3%) | Mean (SD): 45.0 (11.2) | 0: 0 (0%) | 0: 411 (80.6%) | 0: 404 (79.2%) | 0: 387 (75.9%) | Mean (SD): 127 (42.5) | Mean (SD): 182 (30.3) | Mean (SD): 33.1 (6.54) | Mean (SD): 0.595 (0.0866) | 0: 166 (32.5%) | 0: 1 (0.2%) |
|  | 2: 279 (54.7%) | Median [Min, Max]: 46.0 [25.0, 64.0] | 1: 0 (0%) | 1: 99 (19.4%) | 1: 106 (20.8%) | 1: 123 (24.1%) | Median [Min, Max]: 113 [36.0, 396] | Median [Min, Max]: 176 [112, 300] | Median [Min, Max]: 32.3 [18.0, 62.0] | Median [Min, Max]: 0.593 [0.266, 1.00] | 1: 103 (20.2%) | 1: 47 (9.2%) |
|  |  |  | Missing: 510 (100%) | Missing: 0 (0%) |  |  |  |  |  |  | 2: 241 (47.3%) | 2: 117 (22.9%) |
|  |  |  |  |  |  |  |  |  |  |  |  | 3: 345 (67.6%) |
| **Qatar** | : | : | : | : | : | : | : | : | : | : | : | : |
| **(N=1132)** | 1: 447 (39.5%) | Mean (SD): 42.3 (10.0) | 0: 0 (0%) | 0: 969 (85.6%) | 0: 891 (78.7%) | 0: 874 (77.2%) | Mean (SD): 98.4 (41.7) | Mean (SD): 167 (37.3) | Mean (SD): 30.9 (6.92) | Mean (SD): 0.606 (0.102) | 0: 567 (50.1%) | 0: 13 (1.1%) |
|  | 2: 685 (60.5%) | Median [Min, Max]: 42.0 [25.0, 64.0] | 1: 0 (0%) | 1: 163 (14.4%) | 1: 241 (21.3%) | 1: 258 (22.8%) | Median [Min, Max]: 86.0 [37.0, 326] | Median [Min, Max]: 165 [100, 400] | Median [Min, Max]: 30.1 [14.3, 60.4] | Median [Min, Max]: 0.600 [0.319, 1.34] | 1: 209 (18.5%) | 1: 211 (18.6%) |
|  |  |  | Missing: 1132 (100%) | Missing: 0 (0%) |  |  |  |  |  |  | 2: 356 (31.4%) | 2: 338 (29.9%) |
|  |  |  |  |  |  |  |  |  |  |  |  | 3: 570 (50.4%) |
| **Republic of Moldova** | : | : | : | : | : | : | : | : | : | : | : | : |
| **(N=2610)** | 1: 946 (36.2%) | Mean (SD): 46.9 (11.5) | 0: 1245 (47.7%) | 0: 2097 (80.3%) | 0: 2396 (91.8%) | 0: 1765 (67.6%) | Mean (SD): 95.9 (29.5) | Mean (SD): 181 (38.8) | Mean (SD): 28.2 (5.88) | Mean (SD): 0.549 (0.0949) | 0: 1751 (67.1%) | 0: 32 (1.2%) |
|  | 2: 1664 (63.8%) | Median [Min, Max]: 48.0 [25.0, 64.0] | 1: 1365 (52.3%) | 1: 513 (19.7%) | 1: 214 (8.2%) | 1: 845 (32.4%) | Median [Min, Max]: 90.0 [37.8, 502] | Median [Min, Max]: 179 [100, 397] | Median [Min, Max]: 27.3 [12.1, 66.2] | Median [Min, Max]: 0.537 [0.275, 1.27] | 1: 125 (4.8%) | 1: 841 (32.2%) |
|  |  |  | Missing: 0 (0%) | Missing: 0 (0%) |  |  |  |  |  |  | 2: 734 (28.1%) | 2: 870 (33.3%) |
|  |  |  |  |  |  |  |  |  |  |  |  | 3: 867 (33.2%) |
| **Rwanda** | : | : | : | : | : | : | : | : | : | : | : | : |
| **(N=5063)** | 1: 1918 (37.9%) | Mean (SD): 39.8 (10.8) | 0: 0 (0%) | 0: 4161 (82.2%) | 0: 5042 (99.6%) | 0: 4872 (96.2%) | Mean (SD): 70.3 (21.4) | Mean (SD): 126 (31.2) | Mean (SD): 22.6 (3.60) | Mean (SD): 0.485 (0.0640) | 0: 4878 (96.3%) | 0: 374 (7.4%) |
|  | 2: 3145 (62.1%) | Median [Min, Max]: 38.0 [25.0, 64.0] | 1: 0 (0%) | 1: 902 (17.8%) | 1: 21 (0.4%) | 1: 191 (3.8%) | Median [Min, Max]: 66.6 [36.0, 450] | Median [Min, Max]: 118 [100, 401] | Median [Min, Max]: 22.1 [11.6, 62.3] | Median [Min, Max]: 0.475 [0.224, 1.11] | 1: 88 (1.7%) | 1: 3775 (74.6%) |
|  |  |  | Missing: 5063 (100%) | Missing: 0 (0%) |  |  |  |  |  |  | 2: 97 (1.9%) | 2: 727 (14.4%) |
|  |  |  |  |  |  |  |  |  |  |  |  | 3: 187 (3.7%) |
| **Samoa** | : | : | : | : | : | : | : | : | : | : | : | : |
| **(N=1134)** | 1: 446 (39.3%) | Mean (SD): 43.4 (10.9) | 0: 0 (0%) | 0: 852 (75.1%) | 0: 1052 (92.8%) | 0: 1034 (91.2%) | Mean (SD): 124 (45.7) | Mean (SD): 174 (28.7) | Mean (SD): 33.5 (7.22) | Mean (SD): 0.624 (0.0980) | 0: 394 (34.7%) | 0: 4 (0.4%) |
|  | 2: 688 (60.7%) | Median [Min, Max]: 43.0 [25.0, 64.0] | 1: 0 (0%) | 1: 282 (24.9%) | 1: 82 (7.2%) | 1: 100 (8.8%) | Median [Min, Max]: 112 [36.0, 443] | Median [Min, Max]: 162 [143, 300] | Median [Min, Max]: 32.8 [11.5, 61.0] | Median [Min, Max]: 0.626 [0.202, 1.07] | 1: 294 (25.9%) | 1: 111 (9.8%) |
|  |  |  | Missing: 1134 (100%) | Missing: 0 (0%) |  |  |  |  |  |  | 2: 446 (39.3%) | 2: 270 (23.8%) |
|  |  |  |  |  |  |  |  |  |  |  |  | 3: 749 (66.0%) |
| **Sao Tome and Principe** | : | : | : | : | : | : | : | : | : | : | : | : |
| **(N=1392)** | 1: 548 (39.4%) | Mean (SD): 40.2 (10.4) | 0: 569 (40.9%) | 0: 1319 (94.8%) | 0: 1321 (94.9%) | 0: 1036 (74.4%) | Mean (SD): 102 (30.6) | Mean (SD): 208 (32.9) | Mean (SD): 25.7 (5.62) | Mean (SD): 0.533 (0.0862) | 0: 1141 (82.0%) | 0: 69 (5.0%) |
|  | 2: 844 (60.6%) | Median [Min, Max]: 38.0 [25.0, 64.0] | 1: 823 (59.1%) | 1: 73 (5.2%) | 1: 71 (5.1%) | 1: 356 (25.6%) | Median [Min, Max]: 99.0 [36.0, 450] | Median [Min, Max]: 201 [100, 352] | Median [Min, Max]: 24.8 [14.0, 69.9] | Median [Min, Max]: 0.525 [0.200, 1.03] | 1: 21 (1.5%) | 1: 652 (46.8%) |
|  |  |  | Missing: 0 (0%) | Missing: 0 (0%) |  |  |  |  |  |  | 2: 230 (16.5%) | 2: 416 (29.9%) |
|  |  |  |  |  |  |  |  |  |  |  |  | 3: 255 (18.3%) |
| **Seychelles** | : | : | : | : | : | : | : | : | : | : | : | : |
| **(N=1231)** | 1: 553 (44.9%) | Mean (SD): 45.1 (11.1) | 0: 0 (0%) | 0: 987 (80.2%) | 0: 1148 (93.3%) | 0: 838 (68.1%) | Mean (SD): 109 (39.4) | Mean (SD): 213 (49.7) | Mean (SD): 27.4 (5.80) | Mean (SD): 0.552 (0.0853) | 0: 888 (72.1%) | 0: 42 (3.4%) |
|  | 2: 678 (55.1%) | Median [Min, Max]: 45.0 [25.0, 64.0] | 1: 0 (0%) | 1: 244 (19.8%) | 1: 83 (6.7%) | 1: 393 (31.9%) | Median [Min, Max]: 99.0 [50.4, 452] | Median [Min, Max]: 206 [90.9, 448] | Median [Min, Max]: 26.9 [16.0, 53.3] | Median [Min, Max]: 0.547 [0.357, 0.848] | 1: 56 (4.5%) | 1: 409 (33.2%) |
|  |  |  | Missing: 1231 (100%) | Missing: 0 (0%) |  |  |  |  |  |  | 2: 287 (23.3%) | 2: 436 (35.4%) |
|  |  |  |  |  |  |  |  |  |  |  |  | 3: 344 (27.9%) |
| **Solomon Islands** | : | : | : | : | : | : | : | : | : | : | : | : |
| **(N=1322)** | 1: 593 (44.9%) | Mean (SD): 41.8 (10.2) | 0: 0 (0%) | 0: 853 (64.5%) | 0: 1269 (96.0%) | 0: 1157 (87.5%) | Mean (SD): 82.4 (32.2) | Mean (SD): 177 (43.7) | Mean (SD): 27.6 (5.71) | Mean (SD): 0.552 (0.0901) | 0: 941 (71.2%) | 0: 22 (1.7%) |
|  | 2: 729 (55.1%) | Median [Min, Max]: 41.0 [25.0, 64.0] | 1: 0 (0%) | 1: 469 (35.5%) | 1: 53 (4.0%) | 1: 165 (12.5%) | Median [Min, Max]: 75.6 [36.0, 409] | Median [Min, Max]: 173 [86.2, 391] | Median [Min, Max]: 26.8 [11.7, 64.2] | Median [Min, Max]: 0.541 [0.212, 1.28] | 1: 155 (11.7%) | 1: 459 (34.7%) |
|  |  |  | Missing: 1322 (100%) | Missing: 0 (0%) |  |  |  |  |  |  | 2: 226 (17.1%) | 2: 454 (34.3%) |
|  |  |  |  |  |  |  |  |  |  |  |  | 3: 387 (29.3%) |
| **Sri Lanka** | : | : | : | : | : | : | : | : | : | : | : | : |
| **(N=3401)** | 1: 1308 (38.5%) | Mean (SD): 45.0 (10.8) | 0: 0 (0%) | 0: 2945 (86.6%) | 0: 2969 (87.3%) | 0: 2835 (83.4%) | Mean (SD): 86.4 (39.6) | Mean (SD): 159 (46.0) | Mean (SD): 23.6 (4.70) | Mean (SD): 0.536 (0.0843) | 0: 3149 (92.6%) | 0: 431 (12.7%) |
|  | 2: 2093 (61.5%) | Median [Min, Max]: 45.0 [25.0, 64.0] | 1: 0 (0%) | 1: 456 (13.4%) | 1: 432 (12.7%) | 1: 566 (16.6%) | Median [Min, Max]: 79.0 [36.0, 499] | Median [Min, Max]: 155 [100, 400] | Median [Min, Max]: 23.4 [12.5, 75.6] | Median [Min, Max]: 0.533 [0.206, 1.27] | 1: 77 (2.3%) | 1: 1786 (52.5%) |
|  |  |  | Missing: 3401 (100%) | Missing: 0 (0%) |  |  |  |  |  |  | 2: 175 (5.1%) | 2: 923 (27.1%) |
|  |  |  |  |  |  |  |  |  |  |  |  | 3: 261 (7.7%) |
| **Sudan** | : | : | : | : | : | : | : | : | : | : | : | : |
| **(N=4978)** | 1: 1797 (36.1%) | Mean (SD): 40.9 (10.5) | 0: 3305 (66.4%) | 0: 4666 (93.7%) | 0: 4680 (94.0%) | 0: 4451 (89.4%) | Mean (SD): 87.9 (44.2) | Mean (SD): 156 (43.1) | Mean (SD): 24.3 (5.83) | Mean (SD): 0.527 (0.0868) | 0: 4296 (86.3%) | 0: 667 (13.4%) |
|  | 2: 3181 (63.9%) | Median [Min, Max]: 40.0 [25.0, 64.0] | 1: 1673 (33.6%) | 1: 312 (6.3%) | 1: 298 (6.0%) | 1: 527 (10.6%) | Median [Min, Max]: 77.4 [36.0, 472] | Median [Min, Max]: 150 [100, 401] | Median [Min, Max]: 23.3 [11.9, 72.2] | Median [Min, Max]: 0.520 [0.201, 1.22] | 1: 184 (3.7%) | 1: 2423 (48.7%) |
|  |  |  | Missing: 0 (0%) | Missing: 0 (0%) |  |  |  |  |  |  | 2: 498 (10.0%) | 2: 1180 (23.7%) |
|  |  |  |  |  |  |  |  |  |  |  |  | 3: 708 (14.2%) |
| **Tajikistan** | : | : | : | : | : | : | : | : | : | : | : | : |
| **(N=2058)** | 1: 857 (41.6%) | Mean (SD): 42.2 (10.8) | 0: 0 (0%) | 0: 1944 (94.5%) | 0: 1992 (96.8%) | 0: 1579 (76.7%) | Mean (SD): 96.3 (32.6) | Mean (SD): 157 (38.2) | Mean (SD): 26.7 (5.37) | Mean (SD): 0.531 (0.107) | 0: 1622 (78.8%) | 0: 56 (2.7%) |
|  | 2: 1201 (58.4%) | Median [Min, Max]: 42.0 [25.0, 64.0] | 1: 0 (0%) | 1: 114 (5.5%) | 1: 66 (3.2%) | 1: 479 (23.3%) | Median [Min, Max]: 90.0 [36.0, 436] | Median [Min, Max]: 153 [100, 355] | Median [Min, Max]: 26.0 [11.1, 53.7] | Median [Min, Max]: 0.533 [0.200, 1.00] | 1: 79 (3.8%) | 1: 804 (39.1%) |
|  |  |  | Missing: 2058 (100%) | Missing: 0 (0%) |  |  |  |  |  |  | 2: 357 (17.3%) | 2: 722 (35.1%) |
|  |  |  |  |  |  |  |  |  |  |  |  | 3: 476 (23.1%) |
| **Timor Leste** | : | : | : | : | : | : | : | : | : | : | : | : |
| **(N=1817)** | 1: 785 (43.2%) | Mean (SD): 42.1 (11.0) | 0: 0 (0%) | 0: 1201 (66.1%) | 0: 1806 (99.4%) | 0: 1635 (90.0%) | Mean (SD): 77.5 (27.5) | Mean (SD): 148 (37.0) | Mean (SD): 21.4 (4.13) | Mean (SD): 0.501 (0.0676) | 0: 1770 (97.4%) | 0: 343 (18.9%) |
|  | 2: 1032 (56.8%) | Median [Min, Max]: 41.0 [25.0, 64.0] | 1: 0 (0%) | 1: 616 (33.9%) | 1: 11 (0.6%) | 1: 182 (10.0%) | Median [Min, Max]: 73.8 [36.0, 394] | Median [Min, Max]: 142 [100, 363] | Median [Min, Max]: 20.8 [11.8, 68.8] | Median [Min, Max]: 0.493 [0.273, 0.780] | 1: 20 (1.1%) | 1: 1232 (67.8%) |
|  |  |  | Missing: 1817 (100%) | Missing: 0 (0%) |  |  |  |  |  |  | 2: 27 (1.5%) | 2: 194 (10.7%) |
|  |  |  |  |  |  |  |  |  |  |  |  | 3: 48 (2.6%) |
| **Togo** | : | : | : | : | : | : | : | : | : | : | : | : |
| **(N=1110)** | 1: 496 (44.7%) | Mean (SD): 40.1 (10.8) | 0: 0 (0%) | 0: 1007 (90.7%) | 0: 1098 (98.9%) | 0: 1009 (90.9%) | Mean (SD): 85.2 (22.7) | Mean (SD): 171 (21.0) | Mean (SD): 23.7 (4.76) | Mean (SD): 0.505 (0.0756) | 0: 1013 (91.3%) | 0: 87 (7.8%) |
|  | 2: 614 (55.3%) | Median [Min, Max]: 39.0 [25.0, 64.0] | 1: 0 (0%) | 1: 103 (9.3%) | 1: 12 (1.1%) | 1: 101 (9.1%) | Median [Min, Max]: 84.4 [37.7, 278] | Median [Min, Max]: 165 [150, 300] | Median [Min, Max]: 22.9 [12.7, 59.5] | Median [Min, Max]: 0.488 [0.224, 1.09] | 1: 37 (3.3%) | 1: 705 (63.5%) |
|  |  |  | Missing: 1110 (100%) | Missing: 0 (0%) |  |  |  |  |  |  | 2: 60 (5.4%) | 2: 220 (19.8%) |
|  |  |  |  |  |  |  |  |  |  |  |  | 3: 98 (8.8%) |
| **Tokelau** | : | : | : | : | : | : | : | : | : | : | : | : |
| **(N=415)** | 1: 198 (47.7%) | Mean (SD): 43.3 (11.6) | 0: 0 (0%) | 0: 181 (43.6%) | 0: 319 (76.9%) | 0: 321 (77.3%) | Mean (SD): 149 (62.6) | Mean (SD): 204 (45.1) | Mean (SD): 34.3 (6.58) | Mean (SD): 0.612 (0.0882) | 0: 110 (26.5%) | 0: 1 (0.2%) |
|  | 2: 217 (52.3%) | Median [Min, Max]: 43.0 [25.0, 64.0] | 1: 0 (0%) | 1: 234 (56.4%) | 1: 96 (23.1%) | 1: 94 (22.7%) | Median [Min, Max]: 126 [83.9, 440] | Median [Min, Max]: 202 [103, 356] | Median [Min, Max]: 34.2 [12.7, 73.1] | Median [Min, Max]: 0.605 [0.392, 1.18] | 1: 54 (13.0%) | 1: 23 (5.5%) |
|  |  |  | Missing: 415 (100%) | Missing: 0 (0%) |  |  |  |  |  |  | 2: 251 (60.5%) | 2: 85 (20.5%) |
|  |  |  |  |  |  |  |  |  |  |  |  | 3: 306 (73.7%) |
| **Turkmenistan** | : | : | : | : | : | : | : | : | : | : | : | : |
| **(N=3170)** | 1: 1374 (43.3%) | Mean (SD): 42.1 (10.7) | 0: 1608 (50.7%) | 0: 3040 (95.9%) | 0: 3108 (98.0%) | 0: 2646 (83.5%) | Mean (SD): 94.2 (31.8) | Mean (SD): 168 (45.4) | Mean (SD): 26.4 (4.87) | Mean (SD): 0.551 (0.0796) | 0: 2543 (80.2%) | 0: 74 (2.3%) |
|  | 2: 1796 (56.7%) | Median [Min, Max]: 41.0 [25.0, 64.0] | 1: 1562 (49.3%) | 1: 130 (4.1%) | 1: 62 (2.0%) | 1: 524 (16.5%) | Median [Min, Max]: 90.0 [36.0, 454] | Median [Min, Max]: 163 [100, 401] | Median [Min, Max]: 25.6 [13.8, 52.1] | Median [Min, Max]: 0.548 [0.204, 1.00] | 1: 181 (5.7%) | 1: 1312 (41.4%) |
|  |  |  | Missing: 0 (0%) | Missing: 0 (0%) |  |  |  |  |  |  | 2: 446 (14.1%) | 2: 1155 (36.4%) |
|  |  |  |  |  |  |  |  |  |  |  |  | 3: 629 (19.8%) |
| **Tuvalu** | : | : | : | : | : | : | : | : | : | : | : | : |
| **(N=796)** | 1: 362 (45.5%) | Mean (SD): 44.4 (11.6) | 0: 0 (0%) | 0: 518 (65.1%) | 0: 716 (89.9%) | 0: 665 (83.5%) | Mean (SD): 91.4 (44.8) | Mean (SD): 164 (44.4) | Mean (SD): 34.1 (7.28) | Mean (SD): 0.622 (0.101) | 0: 242 (30.4%) | 0: 3 (0.4%) |
|  | 2: 434 (54.5%) | Median [Min, Max]: 45.0 [25.0, 64.0] | 1: 0 (0%) | 1: 278 (34.9%) | 1: 80 (10.1%) | 1: 131 (16.5%) | Median [Min, Max]: 77.4 [36.0, 369] | Median [Min, Max]: 159 [100, 401] | Median [Min, Max]: 33.3 [12.3, 79.3] | Median [Min, Max]: 0.618 [0.201, 1.24] | 1: 154 (19.3%) | 1: 58 (7.3%) |
|  |  |  | Missing: 796 (100%) | Missing: 0 (0%) |  |  |  |  |  |  | 2: 400 (50.3%) | 2: 174 (21.9%) |
|  |  |  |  |  |  |  |  |  |  |  |  | 3: 561 (70.5%) |
| **Uganda** | : | : | : | : | : | : | : | : | : | : | : | : |
| **(N=2463)** | 1: 1026 (41.7%) | Mean (SD): 39.2 (10.5) | 0: 1838 (74.6%) | 0: 2212 (89.8%) | 0: 2436 (98.9%) | 0: 2244 (91.1%) | Mean (SD): 72.3 (20.4) | Mean (SD): 138 (38.0) | Mean (SD): 23.1 (4.66) | Mean (SD): 0.497 (0.0674) | 0: 2288 (92.9%) | 0: 232 (9.4%) |
|  | 2: 1437 (58.3%) | Median [Min, Max]: 37.0 [25.0, 64.0] | 1: 625 (25.4%) | 1: 251 (10.2%) | 1: 27 (1.1%) | 1: 219 (8.9%) | Median [Min, Max]: 70.2 [36.0, 385] | Median [Min, Max]: 130 [100, 401] | Median [Min, Max]: 22.2 [11.8, 72.0] | Median [Min, Max]: 0.484 [0.213, 1.00] | 1: 68 (2.8%) | 1: 1625 (66.0%) |
|  |  |  | Missing: 0 (0%) | Missing: 0 (0%) |  |  |  |  |  |  | 2: 107 (4.3%) | 2: 429 (17.4%) |
|  |  |  |  |  |  |  |  |  |  |  |  | 3: 177 (7.2%) |
| **United Republic of Tanzania** | : | : | : | : | : | : | : | : | : | : | : | : |
| **(N=1717)** | 1: 768 (44.7%) | Mean (SD): 42.8 (11.3) | 0: 0 (0%) | 0: 1522 (88.6%) | 0: 1674 (97.5%) | 0: 1560 (90.9%) | Mean (SD): 87.1 (27.2) | Mean (SD): 176 (30.0) | Mean (SD): 23.1 (4.94) | Mean (SD): 0.522 (0.0784) | 0: 1575 (91.7%) | 0: 194 (11.3%) |
|  | 2: 949 (55.3%) | Median [Min, Max]: 42.0 [25.0, 64.0] | 1: 0 (0%) | 1: 195 (11.4%) | 1: 43 (2.5%) | 1: 157 (9.1%) | Median [Min, Max]: 82.8 [36.0, 365] | Median [Min, Max]: 166 [131, 300] | Median [Min, Max]: 22.1 [13.1, 63.4] | Median [Min, Max]: 0.506 [0.220, 1.00] | 1: 42 (2.4%) | 1: 1069 (62.3%) |
|  |  |  | Missing: 1717 (100%) | Missing: 0 (0%) |  |  |  |  |  |  | 2: 100 (5.8%) | 2: 309 (18.0%) |
|  |  |  |  |  |  |  |  |  |  |  |  | 3: 145 (8.4%) |
| **Vietnam** | : | : | : | : | : | : | : | : | : | : | : | : |
| **(N=2564)** | 1: 1088 (42.4%) | Mean (SD): 44.3 (10.5) | 0: 1420 (55.4%) | 0: 1925 (75.1%) | 0: 2495 (97.3%) | 0: 2189 (85.4%) | Mean (SD): 72.8 (22.5) | Mean (SD): 179 (47.9) | Mean (SD): 22.4 (3.22) | Mean (SD): 0.498 (0.0575) | 0: 2517 (98.2%) | 0: 233 (9.1%) |
|  | 2: 1476 (57.6%) | Median [Min, Max]: 44.0 [25.0, 64.0] | 1: 1144 (44.6%) | 1: 636 (24.8%) | 1: 69 (2.7%) | 1: 375 (14.6%) | Median [Min, Max]: 70.2 [36.0, 504] | Median [Min, Max]: 174 [88.9, 401] | Median [Min, Max]: 22.1 [14.1, 52.9] | Median [Min, Max]: 0.495 [0.249, 0.936] | 1: 15 (0.6%) | 1: 1868 (72.9%) |
|  |  |  | Missing: 0 (0%) | Missing: 3 (0.1%) |  |  |  |  |  |  | 2: 32 (1.2%) | 2: 414 (16.1%) |
|  |  |  |  |  |  |  |  |  |  |  |  | 3: 49 (1.9%) |
| **Zambia** | : | : | : | : | : | : | : | : | : | : | : | : |
| **(N=2386)** | 1: 944 (39.6%) | Mean (SD): 40.2 (10.7) | 0: 1588 (66.6%) | 0: 2064 (86.5%) | 0: 2351 (98.5%) | 0: 2073 (86.9%) | Mean (SD): 90.4 (29.5) | Mean (SD): 137 (37.4) | Mean (SD): 23.5 (5.03) | Mean (SD): 0.503 (0.0754) | 0: 2185 (91.6%) | 0: 164 (6.9%) |
|  | 2: 1442 (60.4%) | Median [Min, Max]: 39.0 [25.0, 64.0] | 1: 798 (33.4%) | 1: 322 (13.5%) | 1: 35 (1.5%) | 1: 313 (13.1%) | Median [Min, Max]: 88.2 [36.0, 491] | Median [Min, Max]: 128 [100, 317] | Median [Min, Max]: 22.4 [10.1, 75.4] | Median [Min, Max]: 0.490 [0.200, 1.19] | 1: 75 (3.1%) | 1: 1593 (66.8%) |
|  |  |  | Missing: 0 (0%) | Missing: 0 (0%) |  |  |  |  |  |  | 2: 126 (5.3%) | 2: 426 (17.9%) |
|  |  |  |  |  |  |  |  |  |  |  |  | 3: 203 (8.5%) |
| **Overall** | : | : | : | : | : | : | : | : | : | : | : | : |
| **(N=142250)** | 1: 56155 (39.5%) | Mean (SD): 42.5 (11.0) | 0: 47117 (33.1%) | 0: 118333 (83.2%) | 0: 134662 (94.7%) | 0: 116487 (81.9%) | Mean (SD): 91.3 (34.8) | Mean (SD): 166 (43.8) | Mean (SD): 25.4 (6.08) | Mean (SD): 0.534 (0.0916) | 0: 115360 (81.1%) | 0: 11413 (8.0%) |
|  | 2: 86095 (60.5%) | Median [Min, Max]: 42.0 [25.0, 64.0] | 1: 34454 (24.2%) | 1: 23913 (16.8%) | 1: 7588 (5.3%) | 1: 25763 (18.1%) | Median [Min, Max]: 85.5 [36.0, 540] | Median [Min, Max]: 162 [70.0, 650] | Median [Min, Max]: 24.4 [10.0, 79.5] | Median [Min, Max]: 0.523 [0.200, 1.40] | 1: 7426 (5.2%) | 1: 66313 (46.6%) |
|  |  |  | Missing: 60679 (42.7%) | Missing: 4 (0.0%) |  |  |  |  |  |  | 2: 19464 (13.7%) | 2: 37181 (26.1%) |
|  |  |  |  |  |  |  |  |  |  |  |  | 3: 27343 (19.2%) |

Table created with *table1* function in R.

# **Table D in S1 Text. Survey-adjusted as well as age-standardized prevalence (%) of clinical obesity (Figure 1 in manuscript) and BMI-only categories.**

| **Country** | **Sex** | **Survey-weighted (SVY)** | | | | | **Survey-weighted (SVY) and age-standardized** | | | **Design and misspecification effects** | | | |
| --- | --- | --- | --- | --- | --- | --- | --- | --- | --- | --- | --- | --- | --- |
|  |  | **Clinical obesity** | **Prevalence** | **SE** | **Lower 95% CI** | **Upper 95% CI** | **Prevalence** | **Lower 95% CI** | **Upper 95% CI** | **DEFF** | **DEFT** | **MEFF** | **MEFT** |
| Afghanistan | Men | 0 (None) | 0.8636 | 0.0150 | 0.8314 | 0.8904 | 0.4228 | 0.4070 | 0.4360 | 4.1420 | 2.0352 | 2.2469 | 1.4990 |
| Afghanistan | Men | 1 (Pre-clinical) | 0.0333 | 0.0118 | 0.0165 | 0.0658 | 0.0163 | 0.0081 | 0.0322 | 9.3132 | 3.0518 | 3.9871 | 1.9968 |
| Afghanistan | Men | 2 (Clinical) | 0.1032 | 0.0119 | 0.0821 | 0.1289 | 0.0505 | 0.0402 | 0.0631 | 3.3105 | 1.8195 | 1.9397 | 1.3928 |
| Afghanistan | Women | 0 | 0.7527 | 0.0281 | 0.6936 | 0.8036 | 0.3685 | 0.3396 | 0.3935 | 8.0683 | 2.8405 | 4.7944 | 2.1896 |
| Afghanistan | Women | 1 | 0.0628 | 0.0140 | 0.0403 | 0.0964 | 0.0307 | 0.0197 | 0.0472 | 6.3194 | 2.5138 | 4.0357 | 2.0089 |
| Afghanistan | Women | 2 | 0.1846 | 0.0256 | 0.1396 | 0.2400 | 0.0904 | 0.0683 | 0.1175 | 8.2457 | 2.8715 | 4.8209 | 2.1957 |
| Algeria | Men | 0 | 0.8370 | 0.0090 | 0.8185 | 0.8539 | 0.4098 | 0.4007 | 0.4180 | 2.2676 | 1.5059 | 1.2529 | 1.1194 |
| Algeria | Men | 1 | 0.0626 | 0.0056 | 0.0524 | 0.0746 | 0.0306 | 0.0257 | 0.0365 | 2.0617 | 1.4359 | 1.2225 | 1.1057 |
| Algeria | Men | 2 | 0.1005 | 0.0075 | 0.0867 | 0.1161 | 0.0492 | 0.0425 | 0.0568 | 2.3513 | 1.5334 | 1.2441 | 1.1154 |
| Algeria | Women | 0 | 0.6570 | 0.0104 | 0.6364 | 0.6770 | 0.3217 | 0.3116 | 0.3315 | 1.7200 | 1.3115 | 1.2249 | 1.1068 |
| Algeria | Women | 1 | 0.1240 | 0.0069 | 0.1110 | 0.1383 | 0.0607 | 0.0544 | 0.0677 | 1.6004 | 1.2651 | 1.1656 | 1.0796 |
| Algeria | Women | 2 | 0.2190 | 0.0089 | 0.2019 | 0.2370 | 0.1072 | 0.0989 | 0.1160 | 1.6884 | 1.2994 | 1.1691 | 1.0813 |
| American Samoa | Men | 0 | 0.2618 | 0.0116 | 0.2397 | 0.2852 | 0.1282 | 0.1174 | 0.1396 | 0.0015 | 0.0389 | 0.4115 | 0.6415 |
| American Samoa | Men | 1 | 0.1504 | 0.0075 | 0.1362 | 0.1657 | 0.0736 | 0.0667 | 0.0811 | 0.0010 | 0.0310 | 0.2805 | 0.5296 |
| American Samoa | Men | 2 | 0.5878 | 0.0146 | 0.5588 | 0.6162 | 0.2878 | 0.2736 | 0.3017 | 0.0019 | 0.0439 | 0.5248 | 0.7244 |
| American Samoa | Women | 0 | 0.1893 | 0.0149 | 0.1618 | 0.2202 | 0.0927 | 0.0792 | 0.1078 | 0.0034 | 0.0579 | 1.1254 | 1.0609 |
| American Samoa | Women | 1 | 0.2415 | 0.0153 | 0.2129 | 0.2727 | 0.1183 | 0.1042 | 0.1335 | 0.0030 | 0.0544 | 0.9319 | 0.9654 |
| American Samoa | Women | 2 | 0.5692 | 0.0211 | 0.5273 | 0.6101 | 0.2787 | 0.2582 | 0.2987 | 0.0042 | 0.0652 | 1.3442 | 1.1594 |
| Armenia | Men | 0 | 0.8474 | 0.0218 | 0.7997 | 0.8854 | 0.4149 | 0.3915 | 0.4335 | 1.0675 | 1.0332 | 0.9571 | 0.9783 |
| Armenia | Men | 1 | 0.0311 | 0.0106 | 0.0159 | 0.0601 | 0.0152 | 0.0078 | 0.0294 | 1.0850 | 1.0416 | 0.9803 | 0.9901 |
| Armenia | Men | 2 | 0.1215 | 0.0190 | 0.0888 | 0.1640 | 0.0595 | 0.0435 | 0.0803 | 0.9888 | 0.9944 | 0.8666 | 0.9309 |
| Armenia | Women | 0 | 0.7102 | 0.0181 | 0.6734 | 0.7444 | 0.3477 | 0.3297 | 0.3645 | 0.3838 | 0.6195 | 1.3502 | 1.1620 |
| Armenia | Women | 1 | 0.0691 | 0.0097 | 0.0523 | 0.0907 | 0.0338 | 0.0256 | 0.0444 | 0.3513 | 0.5927 | 1.3520 | 1.1628 |
| Armenia | Women | 2 | 0.2207 | 0.0170 | 0.1892 | 0.2558 | 0.1081 | 0.0926 | 0.1252 | 0.4024 | 0.6343 | 1.3546 | 1.1639 |
| Azerbaijan | Men | 0 | 0.8406 | 0.0144 | 0.8103 | 0.8669 | 0.4116 | 0.3967 | 0.4245 | 1.6109 | 1.2692 | 1.0996 | 1.0486 |
| Azerbaijan | Men | 1 | 0.0471 | 0.0092 | 0.0320 | 0.0689 | 0.0231 | 0.0157 | 0.0337 | 1.9663 | 1.4022 | 1.4725 | 1.2135 |
| Azerbaijan | Men | 2 | 0.1122 | 0.0117 | 0.0913 | 0.1373 | 0.0550 | 0.0447 | 0.0672 | 1.4244 | 1.1935 | 0.9146 | 0.9563 |
| Azerbaijan | Women | 0 | 0.7078 | 0.0178 | 0.6717 | 0.7415 | 0.3466 | 0.3289 | 0.3630 | 1.5832 | 1.2583 | 1.7185 | 1.3109 |
| Azerbaijan | Women | 1 | 0.0704 | 0.0092 | 0.0544 | 0.0907 | 0.0345 | 0.0266 | 0.0444 | 1.3316 | 1.1539 | 1.4285 | 1.1952 |
| Azerbaijan | Women | 2 | 0.2218 | 0.0156 | 0.1928 | 0.2537 | 0.1086 | 0.0944 | 0.1242 | 1.4465 | 1.2027 | 1.5195 | 1.2327 |
| Bangladesh | Men | 0 | 0.9752 | 0.0037 | 0.9669 | 0.9815 | 0.4775 | 0.4734 | 0.4805 | 9.5292 | 3.0869 | 1.1657 | 1.0797 |
| Bangladesh | Men | 1 | 0.0042 | 0.0014 | 0.0022 | 0.0083 | 0.0021 | 0.0011 | 0.0040 | 8.5186 | 2.9187 | 1.1609 | 1.0774 |
| Bangladesh | Men | 2 | 0.0205 | 0.0033 | 0.0149 | 0.0282 | 0.0101 | 0.0073 | 0.0138 | 9.5249 | 3.0863 | 1.1386 | 1.0670 |
| Bangladesh | Women | 0 | 0.9102 | 0.0066 | 0.8962 | 0.9224 | 0.4456 | 0.4388 | 0.4516 | 10.7029 | 3.2715 | 1.5109 | 1.2292 |
| Bangladesh | Women | 1 | 0.0267 | 0.0036 | 0.0205 | 0.0347 | 0.0131 | 0.0100 | 0.0170 | 9.7859 | 3.1282 | 1.4059 | 1.1857 |
| Bangladesh | Women | 2 | 0.0632 | 0.0058 | 0.0527 | 0.0756 | 0.0309 | 0.0258 | 0.0370 | 11.3138 | 3.3636 | 1.5722 | 1.2539 |
| Belarus | Men | 0 | 0.7819 | 0.0129 | 0.7555 | 0.8062 | 0.3828 | 0.3699 | 0.3947 | 0.9792 | 0.9896 | 1.5897 | 1.2609 |
| Belarus | Men | 1 | 0.0338 | 0.0050 | 0.0252 | 0.0450 | 0.0165 | 0.0124 | 0.0220 | 0.7580 | 0.8706 | 1.3549 | 1.1640 |
| Belarus | Men | 2 | 0.1843 | 0.0119 | 0.1621 | 0.2088 | 0.0902 | 0.0794 | 0.1022 | 0.9377 | 0.9684 | 1.4965 | 1.2233 |
| Belarus | Women | 0 | 0.6794 | 0.0136 | 0.6522 | 0.7055 | 0.3326 | 0.3193 | 0.3454 | 0.9053 | 0.9514 | 1.8637 | 1.3652 |
| Belarus | Women | 1 | 0.0512 | 0.0065 | 0.0398 | 0.0656 | 0.0250 | 0.0195 | 0.0321 | 0.9345 | 0.9667 | 2.2913 | 1.5137 |
| Belarus | Women | 2 | 0.2694 | 0.0120 | 0.2466 | 0.2936 | 0.1319 | 0.1207 | 0.1437 | 0.7770 | 0.8815 | 1.5527 | 1.2461 |
| Benin | Men | 0 | 0.9496 | 0.0082 | 0.9310 | 0.9634 | 0.4649 | 0.4558 | 0.4717 | 0.4309 | 0.6564 | 2.2151 | 1.4883 |
| Benin | Men | 1 | 0.0141 | 0.0041 | 0.0079 | 0.0250 | 0.0069 | 0.0039 | 0.0122 | 0.3799 | 0.6164 | 1.9510 | 1.3968 |
| Benin | Men | 2 | 0.0363 | 0.0063 | 0.0257 | 0.0510 | 0.0178 | 0.0126 | 0.0250 | 0.3549 | 0.5957 | 1.8173 | 1.3481 |
| Benin | Women | 0 | 0.8887 | 0.0183 | 0.8475 | 0.9199 | 0.4351 | 0.4149 | 0.4504 | 1.2335 | 1.1106 | 5.2857 | 2.2991 |
| Benin | Women | 1 | 0.0274 | 0.0060 | 0.0178 | 0.0421 | 0.0134 | 0.0087 | 0.0206 | 0.4961 | 0.7043 | 2.0109 | 1.4181 |
| Benin | Women | 2 | 0.0838 | 0.0142 | 0.0599 | 0.1162 | 0.0410 | 0.0293 | 0.0569 | 0.9574 | 0.9785 | 4.0846 | 2.0210 |
| Bhutan | Men | 0 | 0.8986 | 0.0084 | 0.8806 | 0.9141 | 0.4399 | 0.4311 | 0.4476 | 0.0403 | 0.2008 | 1.3165 | 1.1474 |
| Bhutan | Men | 1 | 0.0382 | 0.0055 | 0.0287 | 0.0508 | 0.0187 | 0.0140 | 0.0249 | 0.0426 | 0.2063 | 1.4173 | 1.1905 |
| Bhutan | Men | 2 | 0.0632 | 0.0068 | 0.0509 | 0.0781 | 0.0309 | 0.0249 | 0.0383 | 0.0406 | 0.2015 | 1.3079 | 1.1436 |
| Bhutan | Women | 0 | 0.8153 | 0.0096 | 0.7955 | 0.8336 | 0.3992 | 0.3895 | 0.4081 | 0.0240 | 0.1550 | 1.6721 | 1.2931 |
| Bhutan | Women | 1 | 0.0808 | 0.0068 | 0.0683 | 0.0954 | 0.0396 | 0.0334 | 0.0467 | 0.0245 | 0.1565 | 1.7512 | 1.3233 |
| Bhutan | Women | 2 | 0.1039 | 0.0072 | 0.0903 | 0.1192 | 0.0509 | 0.0442 | 0.0583 | 0.0223 | 0.1492 | 1.5207 | 1.2332 |
| Botswana | Men | 0 | 0.9329 | 0.0125 | 0.9038 | 0.9536 | 0.4567 | 0.4425 | 0.4669 | 0.1966 | 0.4434 | 1.6927 | 1.3010 |
| Botswana | Men | 1 | 0.0253 | 0.0088 | 0.0127 | 0.0498 | 0.0124 | 0.0062 | 0.0244 | 0.2505 | 0.5005 | 2.6450 | 1.6264 |
| Botswana | Men | 2 | 0.0419 | 0.0088 | 0.0276 | 0.0630 | 0.0205 | 0.0135 | 0.0308 | 0.1526 | 0.3906 | 1.1790 | 1.0858 |
| Botswana | Women | 0 | 0.7750 | 0.0140 | 0.7463 | 0.8013 | 0.3794 | 0.3654 | 0.3923 | 0.0860 | 0.2933 | 1.7107 | 1.3079 |
| Botswana | Women | 1 | 0.0625 | 0.0076 | 0.0491 | 0.0793 | 0.0306 | 0.0240 | 0.0388 | 0.0759 | 0.2755 | 1.5570 | 1.2478 |
| Botswana | Women | 2 | 0.1625 | 0.0121 | 0.1402 | 0.1877 | 0.0796 | 0.0686 | 0.0919 | 0.0820 | 0.2864 | 1.5800 | 1.2570 |
| Brunei Darussalam | Men | 0 | 0.6966 | 0.0240 | 0.6476 | 0.7416 | 0.3411 | 0.3171 | 0.3631 | 0.0364 | 0.1907 | 1.6609 | 1.2888 |
| Brunei Darussalam | Men | 1 | 0.0345 | 0.0083 | 0.0215 | 0.0551 | 0.0169 | 0.0105 | 0.0270 | 0.0276 | 0.1661 | 1.5960 | 1.2634 |
| Brunei Darussalam | Men | 2 | 0.2688 | 0.0215 | 0.2288 | 0.3130 | 0.1316 | 0.1120 | 0.1533 | 0.0314 | 0.1773 | 1.4192 | 1.1913 |
| Brunei Darussalam | Women | 0 | 0.6420 | 0.0219 | 0.5981 | 0.6838 | 0.3143 | 0.2928 | 0.3348 | 0.0275 | 0.1659 | 1.7480 | 1.3221 |
| Brunei Darussalam | Women | 1 | 0.0864 | 0.0119 | 0.0658 | 0.1127 | 0.0423 | 0.0322 | 0.0552 | 0.0235 | 0.1534 | 1.6641 | 1.2900 |
| Brunei Darussalam | Women | 2 | 0.2715 | 0.0217 | 0.2311 | 0.3161 | 0.1329 | 0.1132 | 0.1547 | 0.0314 | 0.1771 | 1.9696 | 1.4034 |
| Cabo Verde | Men | 0 | 0.9189 | 0.0151 | 0.8841 | 0.9440 | 0.4499 | 0.4329 | 0.4622 | 0.1453 | 0.3812 | 1.8072 | 1.3443 |
| Cabo Verde | Men | 1 | 0.0224 | 0.0097 | 0.0095 | 0.0519 | 0.0110 | 0.0046 | 0.0254 | 0.2070 | 0.4550 | 2.8128 | 1.6772 |
| Cabo Verde | Men | 2 | 0.0587 | 0.0113 | 0.0401 | 0.0852 | 0.0287 | 0.0196 | 0.0417 | 0.1102 | 0.3320 | 1.3229 | 1.1502 |
| Cabo Verde | Women | 0 | 0.7381 | 0.0236 | 0.6894 | 0.7816 | 0.3614 | 0.3375 | 0.3827 | 0.1484 | 0.3853 | 3.2329 | 1.7980 |
| Cabo Verde | Women | 1 | 0.1012 | 0.0159 | 0.0740 | 0.1370 | 0.0496 | 0.0362 | 0.0671 | 0.1442 | 0.3798 | 3.6473 | 1.9098 |
| Cabo Verde | Women | 2 | 0.1606 | 0.0177 | 0.1288 | 0.1985 | 0.0787 | 0.0631 | 0.0972 | 0.1204 | 0.3470 | 2.4383 | 1.5615 |
| Cambodia | Men | 0 | 0.9901 | 0.0024 | 0.9842 | 0.9939 | 0.4848 | 0.4818 | 0.4866 | 0.5445 | 0.7379 | 0.8004 | 0.8947 |
| Cambodia | Men | 1 | 0.0023 | 0.0012 | 0.0008 | 0.0062 | 0.0011 | 0.0004 | 0.0030 | 0.5563 | 0.7458 | 0.8726 | 0.9341 |
| Cambodia | Men | 2 | 0.0076 | 0.0021 | 0.0044 | 0.0129 | 0.0037 | 0.0022 | 0.0063 | 0.5340 | 0.7308 | 0.7695 | 0.8772 |
| Cambodia | Women | 0 | 0.9727 | 0.0035 | 0.9649 | 0.9789 | 0.4763 | 0.4724 | 0.4793 | 0.4540 | 0.6738 | 1.4071 | 1.1862 |
| Cambodia | Women | 1 | 0.0123 | 0.0024 | 0.0085 | 0.0179 | 0.0060 | 0.0041 | 0.0088 | 0.4409 | 0.6640 | 1.5031 | 1.2260 |
| Cambodia | Women | 2 | 0.0149 | 0.0025 | 0.0108 | 0.0206 | 0.0073 | 0.0053 | 0.0101 | 0.4026 | 0.6345 | 1.1588 | 1.0765 |
| Comoros | Men | 0 | 0.9274 | 0.0159 | 0.8895 | 0.9530 | 0.4541 | 0.4355 | 0.4666 | 0.0347 | 0.1862 | 1.2544 | 1.1200 |
| Comoros | Men | 1 | 0.0288 | 0.0102 | 0.0144 | 0.0571 | 0.0141 | 0.0070 | 0.0280 | 0.0344 | 0.1854 | 1.1422 | 1.0687 |
| Comoros | Men | 2 | 0.0437 | 0.0133 | 0.0239 | 0.0787 | 0.0214 | 0.0117 | 0.0385 | 0.0394 | 0.1986 | 1.5190 | 1.2325 |
| Comoros | Women | 0 | 0.6874 | 0.0163 | 0.6545 | 0.7186 | 0.3366 | 0.3205 | 0.3518 | 0.0126 | 0.1121 | 1.3701 | 1.1705 |
| Comoros | Women | 1 | 0.1099 | 0.0105 | 0.0910 | 0.1322 | 0.0538 | 0.0445 | 0.0647 | 0.0114 | 0.1066 | 1.3140 | 1.1463 |
| Comoros | Women | 2 | 0.2027 | 0.0156 | 0.1738 | 0.2350 | 0.0992 | 0.0851 | 0.1151 | 0.0152 | 0.1235 | 1.6264 | 1.2753 |
| Cook Islands | Men | 0 | 0.3076 | 0.0158 | 0.2775 | 0.3393 | 0.1506 | 0.1359 | 0.1661 | 0.0003 | 0.0183 | 0.3176 | 0.5635 |
| Cook Islands | Men | 1 | 0.1025 | 0.0123 | 0.0808 | 0.1293 | 0.0502 | 0.0395 | 0.0633 | 0.0005 | 0.0218 | 0.5094 | 0.7137 |
| Cook Islands | Men | 2 | 0.5899 | 0.0169 | 0.5565 | 0.6225 | 0.2888 | 0.2725 | 0.3048 | 0.0003 | 0.0184 | 0.3284 | 0.5730 |
| Cook Islands | Women | 0 | 0.2712 | 0.0168 | 0.2395 | 0.3054 | 0.1328 | 0.1172 | 0.1495 | 0.0005 | 0.0224 | 0.4419 | 0.6648 |
| Cook Islands | Women | 1 | 0.1826 | 0.0227 | 0.1422 | 0.2314 | 0.0894 | 0.0696 | 0.1133 | 0.0012 | 0.0348 | 1.3074 | 1.1434 |
| Cook Islands | Women | 2 | 0.5462 | 0.0170 | 0.5128 | 0.5792 | 0.2674 | 0.2510 | 0.2836 | 0.0004 | 0.0202 | 0.3858 | 0.6212 |
| Ecuador | Men | 0 | 0.7796 | 0.0167 | 0.7451 | 0.8106 | 0.3817 | 0.3648 | 0.3969 | 2.2628 | 1.5043 | 2.0348 | 1.4265 |
| Ecuador | Men | 1 | 0.0690 | 0.0082 | 0.0546 | 0.0870 | 0.0338 | 0.0267 | 0.0426 | 1.4563 | 1.2068 | 1.2780 | 1.1305 |
| Ecuador | Men | 2 | 0.1514 | 0.0148 | 0.1245 | 0.1828 | 0.0741 | 0.0610 | 0.0895 | 2.3811 | 1.5431 | 2.1559 | 1.4683 |
| Ecuador | Women | 0 | 0.6542 | 0.0149 | 0.6246 | 0.6827 | 0.3203 | 0.3058 | 0.3343 | 1.4663 | 1.2109 | 1.7826 | 1.3352 |
| Ecuador | Women | 1 | 0.1098 | 0.0077 | 0.0956 | 0.1260 | 0.0538 | 0.0468 | 0.0617 | 0.9220 | 0.9602 | 1.0778 | 1.0382 |
| Ecuador | Women | 2 | 0.2359 | 0.0135 | 0.2104 | 0.2635 | 0.1155 | 0.1030 | 0.1290 | 1.5305 | 1.2371 | 1.8992 | 1.3781 |
| Eritrea | Men | 0 | 0.9917 | 0.0032 | 0.9823 | 0.9961 | 0.4855 | 0.4809 | 0.4877 | 0.0860 | 0.2933 | 1.3859 | 1.1772 |
| Eritrea | Men | 1 | 0.0032 | 0.0019 | 0.0010 | 0.0101 | 0.0016 | 0.0005 | 0.0050 | 0.0756 | 0.2750 | 1.0733 | 1.0360 |
| Eritrea | Men | 2 | 0.0050 | 0.0024 | 0.0020 | 0.0129 | 0.0025 | 0.0010 | 0.0063 | 0.0795 | 0.2819 | 1.4015 | 1.1838 |
| Eritrea | Women | 0 | 0.9712 | 0.0045 | 0.9611 | 0.9788 | 0.4755 | 0.4705 | 0.4792 | 0.2726 | 0.5221 | 2.4255 | 1.5574 |
| Eritrea | Women | 1 | 0.0119 | 0.0027 | 0.0076 | 0.0187 | 0.0059 | 0.0037 | 0.0091 | 0.2434 | 0.4934 | 2.4244 | 1.5570 |
| Eritrea | Women | 2 | 0.0168 | 0.0032 | 0.0116 | 0.0244 | 0.0082 | 0.0057 | 0.0119 | 0.2357 | 0.4855 | 1.9532 | 1.3976 |
| Eswatini | Men | 0 | 0.8854 | 0.0166 | 0.8485 | 0.9142 | 0.4335 | 0.4154 | 0.4476 | 0.0687 | 0.2622 | 1.3123 | 1.1456 |
| Eswatini | Men | 1 | 0.0347 | 0.0077 | 0.0224 | 0.0534 | 0.0170 | 0.0110 | 0.0262 | 0.0446 | 0.2113 | 0.7453 | 0.8633 |
| Eswatini | Men | 2 | 0.0799 | 0.0149 | 0.0551 | 0.1143 | 0.0391 | 0.0270 | 0.0560 | 0.0758 | 0.2754 | 1.5127 | 1.2299 |
| Eswatini | Women | 0 | 0.6015 | 0.0189 | 0.5640 | 0.6378 | 0.2945 | 0.2761 | 0.3123 | 0.0455 | 0.2133 | 1.6027 | 1.2660 |
| Eswatini | Women | 1 | 0.1374 | 0.0133 | 0.1133 | 0.1656 | 0.0673 | 0.0555 | 0.0811 | 0.0457 | 0.2139 | 1.7416 | 1.3197 |
| Eswatini | Women | 2 | 0.2611 | 0.0158 | 0.2314 | 0.2932 | 0.1278 | 0.1133 | 0.1435 | 0.0395 | 0.1988 | 1.3430 | 1.1589 |
| Ethiopia | Men | 0 | 0.9950 | 0.0019 | 0.9895 | 0.9977 | 0.4872 | 0.4845 | 0.4885 | 3.0109 | 1.7352 | 1.2468 | 1.1166 |
| Ethiopia | Men | 1 | 0.0006 | 0.0004 | 0.0002 | 0.0022 | 0.0003 | 0.0001 | 0.0011 | 1.1194 | 1.0580 | 0.3682 | 0.6068 |
| Ethiopia | Men | 2 | 0.0044 | 0.0019 | 0.0019 | 0.0100 | 0.0021 | 0.0009 | 0.0049 | 3.2730 | 1.8091 | 1.4032 | 1.1846 |
| Ethiopia | Women | 0 | 0.9757 | 0.0033 | 0.9683 | 0.9813 | 0.4777 | 0.4741 | 0.4805 | 1.5538 | 1.2465 | 1.0019 | 1.0009 |
| Ethiopia | Women | 1 | 0.0088 | 0.0020 | 0.0057 | 0.0136 | 0.0043 | 0.0028 | 0.0067 | 1.5065 | 1.2274 | 1.0057 | 1.0029 |
| Ethiopia | Women | 2 | 0.0155 | 0.0026 | 0.0112 | 0.0215 | 0.0076 | 0.0055 | 0.0105 | 1.4931 | 1.2219 | 0.9339 | 0.9664 |
| French Polynesia | Men | 0 | 0.5604 | 0.0239 | 0.5131 | 0.6066 | 0.2743 | 0.2512 | 0.2970 | 0.0363 | 0.1906 | 1.7079 | 1.3069 |
| French Polynesia | Men | 1 | 0.1182 | 0.0158 | 0.0905 | 0.1529 | 0.0579 | 0.0443 | 0.0748 | 0.0376 | 0.1939 | 1.8204 | 1.3492 |
| French Polynesia | Men | 2 | 0.3215 | 0.0235 | 0.2773 | 0.3691 | 0.1574 | 0.1358 | 0.1807 | 0.0395 | 0.1986 | 1.8446 | 1.3582 |
| French Polynesia | Women | 0 | 0.5252 | 0.0206 | 0.4848 | 0.5652 | 0.2571 | 0.2373 | 0.2767 | 0.0251 | 0.1584 | 1.6798 | 1.2961 |
| French Polynesia | Women | 1 | 0.1401 | 0.0130 | 0.1165 | 0.1677 | 0.0686 | 0.0570 | 0.0821 | 0.0209 | 0.1444 | 1.4934 | 1.2220 |
| French Polynesia | Women | 2 | 0.3347 | 0.0201 | 0.2965 | 0.3752 | 0.1639 | 0.1452 | 0.1837 | 0.0269 | 0.1641 | 1.8104 | 1.3455 |
| Georgia | Men | 0 | 0.6478 | 0.0223 | 0.6030 | 0.6902 | 0.3172 | 0.2952 | 0.3379 | 0.4334 | 0.6583 | 1.4281 | 1.1950 |
| Georgia | Men | 1 | 0.0813 | 0.0141 | 0.0576 | 0.1134 | 0.0398 | 0.0282 | 0.0555 | 0.5274 | 0.7262 | 1.9349 | 1.3910 |
| Georgia | Men | 2 | 0.2709 | 0.0209 | 0.2319 | 0.3138 | 0.1326 | 0.1135 | 0.1536 | 0.4416 | 0.6645 | 1.4145 | 1.1893 |
| Georgia | Women | 0 | 0.6213 | 0.0143 | 0.5928 | 0.6489 | 0.3042 | 0.2902 | 0.3177 | 0.1942 | 0.4407 | 1.4921 | 1.2215 |
| Georgia | Women | 1 | 0.0716 | 0.0082 | 0.0571 | 0.0893 | 0.0350 | 0.0280 | 0.0437 | 0.2226 | 0.4718 | 1.7941 | 1.3394 |
| Georgia | Women | 2 | 0.3072 | 0.0132 | 0.2819 | 0.3336 | 0.1504 | 0.1380 | 0.1633 | 0.1822 | 0.4269 | 1.3461 | 1.1602 |
| Iraq | Men | 0 | 0.6881 | 0.0397 | 0.6046 | 0.7610 | 0.3369 | 0.2960 | 0.3726 | 3.7560 | 1.9380 | 1.9754 | 1.4055 |
| Iraq | Men | 1 | 0.1017 | 0.0326 | 0.0529 | 0.1868 | 0.0498 | 0.0259 | 0.0915 | 5.9492 | 2.4391 | 3.6535 | 1.9114 |
| Iraq | Men | 2 | 0.2102 | 0.0308 | 0.1556 | 0.2776 | 0.1029 | 0.0762 | 0.1359 | 2.9188 | 1.7084 | 1.4259 | 1.1941 |
| Iraq | Women | 0 | 0.5124 | 0.0271 | 0.4587 | 0.5658 | 0.2509 | 0.2246 | 0.2770 | 1.6347 | 1.2786 | 1.5093 | 1.2285 |
| Iraq | Women | 1 | 0.1133 | 0.0152 | 0.0865 | 0.1471 | 0.0555 | 0.0423 | 0.0720 | 1.2783 | 1.1306 | 1.0449 | 1.0222 |
| Iraq | Women | 2 | 0.3743 | 0.0256 | 0.3250 | 0.4263 | 0.1832 | 0.1591 | 0.2087 | 1.5569 | 1.2477 | 1.4476 | 1.2032 |
| Kenya | Men | 0 | 0.9398 | 0.0134 | 0.9075 | 0.9613 | 0.4601 | 0.4443 | 0.4707 | 7.5969 | 2.7563 | 4.0569 | 2.0142 |
| Kenya | Men | 1 | 0.0174 | 0.0043 | 0.0107 | 0.0281 | 0.0085 | 0.0052 | 0.0138 | 2.5691 | 1.6028 | 1.2277 | 1.1080 |
| Kenya | Men | 2 | 0.0427 | 0.0130 | 0.0234 | 0.0769 | 0.0209 | 0.0114 | 0.0376 | 9.8790 | 3.1431 | 5.5387 | 2.3534 |
| Kenya | Women | 0 | 0.8386 | 0.0151 | 0.8068 | 0.8661 | 0.4106 | 0.3950 | 0.4240 | 4.0337 | 2.0084 | 3.1785 | 1.7828 |
| Kenya | Women | 1 | 0.0746 | 0.0118 | 0.0545 | 0.1014 | 0.0365 | 0.0267 | 0.0497 | 4.8555 | 2.2035 | 4.2508 | 2.0617 |
| Kenya | Women | 2 | 0.0867 | 0.0099 | 0.0691 | 0.1083 | 0.0425 | 0.0339 | 0.0530 | 2.9748 | 1.7248 | 2.1794 | 1.4763 |
| Kiribati | Men | 0 | 0.6640 | 0.0554 | 0.5485 | 0.7627 | 0.3251 | 0.2685 | 0.3734 | 0.0617 | 0.2484 | 5.3188 | 2.3063 |
| Kiribati | Men | 1 | 0.0778 | 0.0221 | 0.0441 | 0.1336 | 0.0381 | 0.0216 | 0.0654 | 0.0305 | 0.1746 | 2.0913 | 1.4461 |
| Kiribati | Men | 2 | 0.2582 | 0.0409 | 0.1864 | 0.3460 | 0.1264 | 0.0912 | 0.1694 | 0.0392 | 0.1980 | 3.5281 | 1.8783 |
| Kiribati | Women | 0 | 0.4101 | 0.0246 | 0.3629 | 0.4590 | 0.2008 | 0.1777 | 0.2247 | 0.0148 | 0.1216 | 1.2157 | 1.1026 |
| Kiribati | Women | 1 | 0.1885 | 0.0303 | 0.1360 | 0.2552 | 0.0923 | 0.0666 | 0.1249 | 0.0356 | 0.1887 | 3.1695 | 1.7803 |
| Kiribati | Women | 2 | 0.4014 | 0.0236 | 0.3561 | 0.4485 | 0.1965 | 0.1744 | 0.2196 | 0.0138 | 0.1173 | 1.1774 | 1.0851 |
| Kuwait | Men | 0 | 0.6060 | 0.0207 | 0.5647 | 0.6459 | 0.2967 | 0.2765 | 0.3162 | 0.1301 | 0.3607 | 1.0065 | 1.0032 |
| Kuwait | Men | 1 | 0.0972 | 0.0128 | 0.0748 | 0.1254 | 0.0476 | 0.0366 | 0.0614 | 0.1351 | 0.3675 | 1.0848 | 1.0415 |
| Kuwait | Men | 2 | 0.2968 | 0.0192 | 0.2605 | 0.3358 | 0.1453 | 0.1275 | 0.1644 | 0.1281 | 0.3578 | 0.9760 | 0.9879 |
| Kuwait | Women | 0 | 0.5201 | 0.0167 | 0.4874 | 0.5527 | 0.2547 | 0.2386 | 0.2706 | 0.0872 | 0.2953 | 1.0325 | 1.0161 |
| Kuwait | Women | 1 | 0.1314 | 0.0113 | 0.1108 | 0.1552 | 0.0643 | 0.0542 | 0.0760 | 0.0878 | 0.2963 | 1.0398 | 1.0197 |
| Kuwait | Women | 2 | 0.3485 | 0.0158 | 0.3182 | 0.3800 | 0.1706 | 0.1558 | 0.1861 | 0.0860 | 0.2933 | 1.0099 | 1.0049 |
| Kyrgyzstan | Men | 0 | 0.8273 | 0.0160 | 0.7936 | 0.8565 | 0.4050 | 0.3886 | 0.4193 | 2.2606 | 1.5035 | 1.3788 | 1.1742 |
| Kyrgyzstan | Men | 1 | 0.0325 | 0.0075 | 0.0206 | 0.0508 | 0.0159 | 0.0101 | 0.0249 | 2.2414 | 1.4971 | 1.3867 | 1.1776 |
| Kyrgyzstan | Men | 2 | 0.1402 | 0.0135 | 0.1158 | 0.1688 | 0.0686 | 0.0567 | 0.0826 | 1.9019 | 1.3791 | 1.1334 | 1.0646 |
| Kyrgyzstan | Women | 0 | 0.7116 | 0.0144 | 0.6825 | 0.7390 | 0.3484 | 0.3342 | 0.3618 | 1.1944 | 1.0929 | 1.4872 | 1.2195 |
| Kyrgyzstan | Women | 1 | 0.0576 | 0.0076 | 0.0444 | 0.0744 | 0.0282 | 0.0217 | 0.0364 | 1.2553 | 1.1204 | 1.6790 | 1.2958 |
| Kyrgyzstan | Women | 2 | 0.2308 | 0.0136 | 0.2053 | 0.2585 | 0.1130 | 0.1005 | 0.1266 | 1.2263 | 1.1074 | 1.4805 | 1.2168 |
| Lao People's Democratic Republic | Men | 0 | 0.9664 | 0.0072 | 0.9491 | 0.9780 | 0.4732 | 0.4647 | 0.4788 | 0.4854 | 0.6967 | 1.2852 | 1.1337 |
| Lao People's Democratic Republic | Men | 1 | 0.0147 | 0.0042 | 0.0084 | 0.0257 | 0.0072 | 0.0041 | 0.0126 | 0.3709 | 0.6090 | 0.9616 | 0.9806 |
| Lao People's Democratic Republic | Men | 2 | 0.0188 | 0.0052 | 0.0110 | 0.0322 | 0.0092 | 0.0054 | 0.0158 | 0.4418 | 0.6647 | 1.1880 | 1.0900 |
| Lao People's Democratic Republic | Women | 0 | 0.9177 | 0.0107 | 0.8942 | 0.9363 | 0.4493 | 0.4378 | 0.4584 | 0.6389 | 0.7993 | 1.9394 | 1.3926 |
| Lao People's Democratic Republic | Women | 1 | 0.0232 | 0.0044 | 0.0160 | 0.0335 | 0.0113 | 0.0078 | 0.0164 | 0.3598 | 0.5999 | 1.0324 | 1.0161 |
| Lao People's Democratic Republic | Women | 2 | 0.0592 | 0.0100 | 0.0423 | 0.0822 | 0.0290 | 0.0207 | 0.0402 | 0.7658 | 0.8751 | 2.3824 | 1.5435 |
| Lebanon | Men | 0 | 0.6675 | 0.0427 | 0.5793 | 0.7452 | 0.3268 | 0.2836 | 0.3649 | 3.6295 | 1.9051 | 2.9476 | 1.7169 |
| Lebanon | Men | 1 | 0.0331 | 0.0131 | 0.0151 | 0.0711 | 0.0162 | 0.0074 | 0.0348 | 2.3831 | 1.5437 | 2.0701 | 1.4388 |
| Lebanon | Men | 2 | 0.2994 | 0.0432 | 0.2220 | 0.3903 | 0.1466 | 0.1087 | 0.1911 | 3.9437 | 1.9859 | 3.2178 | 1.7938 |
| Lebanon | Women | 0 | 0.7212 | 0.0354 | 0.6469 | 0.7851 | 0.3531 | 0.3167 | 0.3844 | 3.1654 | 1.7792 | 3.5232 | 1.8770 |
| Lebanon | Women | 1 | 0.0667 | 0.0194 | 0.0373 | 0.1164 | 0.0327 | 0.0183 | 0.0570 | 3.0773 | 1.7542 | 4.5157 | 2.1250 |
| Lebanon | Women | 2 | 0.2121 | 0.0234 | 0.1698 | 0.2615 | 0.1038 | 0.0832 | 0.1280 | 1.6619 | 1.2892 | 1.7222 | 1.3123 |
| Lesotho | Men | 0 | 0.9243 | 0.0157 | 0.8873 | 0.9498 | 0.4525 | 0.4344 | 0.4650 | 0.2008 | 0.4481 | 2.1479 | 1.4656 |
| Lesotho | Men | 1 | 0.0355 | 0.0119 | 0.0182 | 0.0679 | 0.0174 | 0.0089 | 0.0332 | 0.2380 | 0.4878 | 2.7716 | 1.6648 |
| Lesotho | Men | 2 | 0.0402 | 0.0118 | 0.0225 | 0.0709 | 0.0197 | 0.0110 | 0.0347 | 0.2069 | 0.4548 | 2.0613 | 1.4357 |
| Lesotho | Women | 0 | 0.6757 | 0.0187 | 0.6381 | 0.7111 | 0.3308 | 0.3124 | 0.3482 | 0.0926 | 0.3044 | 1.9973 | 1.4132 |
| Lesotho | Women | 1 | 0.1514 | 0.0163 | 0.1221 | 0.1863 | 0.0741 | 0.0598 | 0.0912 | 0.1213 | 0.3483 | 3.5630 | 1.8876 |
| Lesotho | Women | 2 | 0.1729 | 0.0135 | 0.1480 | 0.2011 | 0.0847 | 0.0725 | 0.0984 | 0.0747 | 0.2733 | 1.3552 | 1.1641 |
| Libya | Men | 0 | 0.7821 | 0.0185 | 0.7435 | 0.8162 | 0.3829 | 0.3640 | 0.3996 | 0.4004 | 0.6328 | 1.8542 | 1.3617 |
| Libya | Men | 1 | 0.0677 | 0.0124 | 0.0470 | 0.0965 | 0.0331 | 0.0230 | 0.0473 | 0.4865 | 0.6975 | 2.8066 | 1.6753 |
| Libya | Men | 2 | 0.1502 | 0.0147 | 0.1237 | 0.1813 | 0.0736 | 0.0606 | 0.0888 | 0.3346 | 0.5784 | 1.4379 | 1.1991 |
| Libya | Women | 0 | 0.6009 | 0.0283 | 0.5443 | 0.6548 | 0.2942 | 0.2665 | 0.3206 | 0.5691 | 0.7544 | 2.3918 | 1.5466 |
| Libya | Women | 1 | 0.1573 | 0.0178 | 0.1253 | 0.1955 | 0.0770 | 0.0614 | 0.0957 | 0.4097 | 0.6401 | 1.7425 | 1.3200 |
| Libya | Women | 2 | 0.2419 | 0.0202 | 0.2046 | 0.2836 | 0.1184 | 0.1002 | 0.1388 | 0.3781 | 0.6149 | 1.5039 | 1.2263 |
| Malawi | Men | 0 | 0.9911 | 0.0025 | 0.9845 | 0.9949 | 0.4852 | 0.4820 | 0.4871 | 0.7212 | 0.8493 | 0.2904 | 0.5389 |
| Malawi | Men | 1 | 0.0044 | 0.0019 | 0.0019 | 0.0101 | 0.0021 | 0.0009 | 0.0050 | 0.8067 | 0.8982 | 0.4024 | 0.6343 |
| Malawi | Men | 2 | 0.0045 | 0.0016 | 0.0023 | 0.0091 | 0.0022 | 0.0011 | 0.0045 | 0.5651 | 0.7517 | 0.1900 | 0.4359 |
| Malawi | Women | 0 | 0.8868 | 0.0116 | 0.8618 | 0.9078 | 0.4342 | 0.4219 | 0.4444 | 1.4101 | 1.1875 | 2.1140 | 1.4540 |
| Malawi | Women | 1 | 0.0594 | 0.0088 | 0.0443 | 0.0793 | 0.0291 | 0.0217 | 0.0388 | 1.4417 | 1.2007 | 2.6427 | 1.6256 |
| Malawi | Women | 2 | 0.0538 | 0.0074 | 0.0410 | 0.0702 | 0.0263 | 0.0201 | 0.0344 | 1.1122 | 1.0546 | 1.3692 | 1.1701 |
| Mongolia | Men | 0 | 0.7964 | 0.0099 | 0.7764 | 0.8151 | 0.3899 | 0.3801 | 0.3991 | 0.1464 | 0.3826 | 1.3560 | 1.1645 |
| Mongolia | Men | 1 | 0.0405 | 0.0048 | 0.0321 | 0.0510 | 0.0198 | 0.0157 | 0.0250 | 0.1431 | 0.3783 | 1.3733 | 1.1719 |
| Mongolia | Men | 2 | 0.1631 | 0.0091 | 0.1461 | 0.1816 | 0.0798 | 0.0715 | 0.0889 | 0.1466 | 0.3828 | 1.3458 | 1.1601 |
| Mongolia | Women | 0 | 0.7492 | 0.0099 | 0.7293 | 0.7680 | 0.3668 | 0.3571 | 0.3760 | 0.1278 | 0.3575 | 1.3776 | 1.1737 |
| Mongolia | Women | 1 | 0.0634 | 0.0053 | 0.0538 | 0.0746 | 0.0310 | 0.0263 | 0.0365 | 0.1160 | 0.3407 | 1.2715 | 1.1276 |
| Mongolia | Women | 2 | 0.1874 | 0.0089 | 0.1706 | 0.2054 | 0.0918 | 0.0835 | 0.1006 | 0.1277 | 0.3573 | 1.3543 | 1.1638 |
| Morocco | Men | 0 | 0.8707 | 0.0113 | 0.8469 | 0.8913 | 0.4263 | 0.4146 | 0.4364 | 0.0008 | 0.0276 | 1.3162 | 1.1473 |
| Morocco | Men | 1 | 0.0579 | 0.0080 | 0.0441 | 0.0756 | 0.0283 | 0.0216 | 0.0370 | 0.0008 | 0.0279 | 1.4600 | 1.2083 |
| Morocco | Men | 2 | 0.0714 | 0.0086 | 0.0563 | 0.0902 | 0.0350 | 0.0276 | 0.0442 | 0.0007 | 0.0274 | 1.2221 | 1.1055 |
| Morocco | Women | 0 | 0.6649 | 0.0103 | 0.6443 | 0.6848 | 0.3255 | 0.3155 | 0.3353 | 0.0003 | 0.0181 | 1.0913 | 1.0446 |
| Morocco | Women | 1 | 0.1490 | 0.0080 | 0.1340 | 0.1654 | 0.0730 | 0.0656 | 0.0810 | 0.0003 | 0.0185 | 1.1849 | 1.0885 |
| Morocco | Women | 2 | 0.1861 | 0.0084 | 0.1702 | 0.2031 | 0.0911 | 0.0834 | 0.0994 | 0.0003 | 0.0177 | 1.0153 | 1.0076 |
| Myanmar | Men | 0 | 0.9660 | 0.0084 | 0.9451 | 0.9791 | 0.4729 | 0.4627 | 0.4794 | 20.9087 | 4.5726 | 6.6011 | 2.5693 |
| Myanmar | Men | 1 | 0.0064 | 0.0023 | 0.0032 | 0.0128 | 0.0031 | 0.0016 | 0.0063 | 7.8274 | 2.7978 | 2.5463 | 1.5957 |
| Myanmar | Men | 2 | 0.0276 | 0.0064 | 0.0174 | 0.0434 | 0.0135 | 0.0085 | 0.0213 | 15.0008 | 3.8731 | 4.7135 | 2.1711 |
| Myanmar | Women | 0 | 0.9190 | 0.0071 | 0.9040 | 0.9318 | 0.4499 | 0.4426 | 0.4562 | 6.4728 | 2.5442 | 3.2942 | 1.8150 |
| Myanmar | Women | 1 | 0.0152 | 0.0029 | 0.0105 | 0.0221 | 0.0075 | 0.0051 | 0.0108 | 5.3758 | 2.3186 | 3.4193 | 1.8491 |
| Myanmar | Women | 2 | 0.0658 | 0.0059 | 0.0552 | 0.0783 | 0.0322 | 0.0270 | 0.0383 | 5.3884 | 2.3213 | 2.6267 | 1.6207 |
| Nauru | Men | 0 | 0.2410 | 0.0174 | 0.2086 | 0.2767 | 0.1180 | 0.1021 | 0.1355 | 0.0007 | 0.0269 | 0.5691 | 0.7544 |
| Nauru | Men | 1 | 0.2657 | 0.0296 | 0.2118 | 0.3277 | 0.1301 | 0.1037 | 0.1605 | 0.0020 | 0.0445 | 1.5572 | 1.2479 |
| Nauru | Men | 2 | 0.4933 | 0.0337 | 0.4278 | 0.5590 | 0.2415 | 0.2094 | 0.2737 | 0.0020 | 0.0446 | 1.5600 | 1.2490 |
| Nauru | Women | 0 | 0.2531 | 0.0179 | 0.2197 | 0.2897 | 0.1239 | 0.1076 | 0.1418 | 0.0008 | 0.0277 | 0.6780 | 0.8234 |
| Nauru | Women | 1 | 0.3021 | 0.0217 | 0.2613 | 0.3463 | 0.1479 | 0.1279 | 0.1696 | 0.0010 | 0.0319 | 0.8796 | 0.9379 |
| Nauru | Women | 2 | 0.4448 | 0.0200 | 0.4059 | 0.4843 | 0.2178 | 0.1988 | 0.2371 | 0.0007 | 0.0272 | 0.6379 | 0.7987 |
| Nepal | Men | 0 | 0.9548 | 0.0081 | 0.9359 | 0.9683 | 0.4675 | 0.4582 | 0.4741 | 3.2101 | 1.7917 | 2.0300 | 1.4248 |
| Nepal | Men | 1 | 0.0082 | 0.0025 | 0.0045 | 0.0148 | 0.0040 | 0.0022 | 0.0072 | 1.5780 | 1.2562 | 0.7159 | 0.8461 |
| Nepal | Men | 2 | 0.0370 | 0.0077 | 0.0245 | 0.0554 | 0.0181 | 0.0120 | 0.0271 | 3.4972 | 1.8701 | 2.4130 | 1.5534 |
| Nepal | Women | 0 | 0.9333 | 0.0077 | 0.9164 | 0.9469 | 0.4569 | 0.4487 | 0.4636 | 2.3063 | 1.5187 | 2.2758 | 1.5086 |
| Nepal | Women | 1 | 0.0238 | 0.0042 | 0.0168 | 0.0336 | 0.0117 | 0.0082 | 0.0165 | 1.8407 | 1.3567 | 1.7645 | 1.3283 |
| Nepal | Women | 2 | 0.0429 | 0.0061 | 0.0324 | 0.0567 | 0.0210 | 0.0158 | 0.0278 | 2.2091 | 1.4863 | 2.2010 | 1.4836 |
| Niue | Men | 0 | 0.3270 | 0.0310 | 0.2694 | 0.3903 | 0.1601 | 0.1319 | 0.1911 | 0.0005 | 0.0226 | 1.0064 | 1.0032 |
| Niue | Men | 1 | 0.2011 | 0.0265 | 0.1541 | 0.2581 | 0.0985 | 0.0754 | 0.1264 | 0.0005 | 0.0227 | 1.0135 | 1.0067 |
| Niue | Men | 2 | 0.4719 | 0.0329 | 0.4083 | 0.5365 | 0.2311 | 0.1999 | 0.2627 | 0.0005 | 0.0226 | 0.9969 | 0.9984 |
| Niue | Women | 0 | 0.3269 | 0.0281 | 0.2743 | 0.3842 | 0.1600 | 0.1343 | 0.1881 | 0.0004 | 0.0211 | 1.0009 | 1.0004 |
| Niue | Women | 1 | 0.2088 | 0.0246 | 0.1647 | 0.2610 | 0.1022 | 0.0806 | 0.1278 | 0.0005 | 0.0213 | 1.0312 | 1.0155 |
| Niue | Women | 2 | 0.4643 | 0.0299 | 0.4065 | 0.5231 | 0.2273 | 0.1990 | 0.2561 | 0.0004 | 0.0211 | 0.9958 | 0.9979 |
| Qatar | Men | 0 | 0.5782 | 0.0289 | 0.5207 | 0.6337 | 0.2831 | 0.2549 | 0.3102 | 0.0232 | 0.1523 | 1.5492 | 1.2447 |
| Qatar | Men | 1 | 0.2047 | 0.0263 | 0.1579 | 0.2612 | 0.1002 | 0.0773 | 0.1279 | 0.0288 | 0.1696 | 2.1239 | 1.4574 |
| Qatar | Men | 2 | 0.2170 | 0.0274 | 0.1681 | 0.2755 | 0.1063 | 0.0823 | 0.1349 | 0.0298 | 0.1727 | 1.8993 | 1.3782 |
| Qatar | Women | 0 | 0.4596 | 0.0208 | 0.4192 | 0.5005 | 0.2250 | 0.2052 | 0.2450 | 0.0110 | 0.1050 | 1.1974 | 1.0943 |
| Qatar | Women | 1 | 0.1971 | 0.0176 | 0.1648 | 0.2340 | 0.0965 | 0.0807 | 0.1145 | 0.0125 | 0.1116 | 1.3811 | 1.1752 |
| Qatar | Women | 2 | 0.3433 | 0.0189 | 0.3073 | 0.3812 | 0.1681 | 0.1505 | 0.1866 | 0.0100 | 0.1002 | 1.0450 | 1.0223 |
| Republic of Moldova | Men | 0 | 0.7832 | 0.0172 | 0.7477 | 0.8150 | 0.3835 | 0.3661 | 0.3990 | 0.2236 | 0.4728 | 1.4642 | 1.2100 |
| Republic of Moldova | Men | 1 | 0.0395 | 0.0079 | 0.0265 | 0.0583 | 0.0193 | 0.0130 | 0.0286 | 0.2137 | 0.4623 | 1.5838 | 1.2585 |
| Republic of Moldova | Men | 2 | 0.1773 | 0.0151 | 0.1496 | 0.2089 | 0.0868 | 0.0732 | 0.1023 | 0.2014 | 0.4488 | 1.2711 | 1.1274 |
| Republic of Moldova | Women | 0 | 0.6791 | 0.0146 | 0.6498 | 0.7071 | 0.3325 | 0.3181 | 0.3462 | 0.1276 | 0.3572 | 1.5276 | 1.2360 |
| Republic of Moldova | Women | 1 | 0.0529 | 0.0065 | 0.0415 | 0.0673 | 0.0259 | 0.0203 | 0.0329 | 0.1106 | 0.3326 | 1.4177 | 1.1907 |
| Republic of Moldova | Women | 2 | 0.2680 | 0.0141 | 0.2413 | 0.2965 | 0.1312 | 0.1181 | 0.1452 | 0.1315 | 0.3627 | 1.5248 | 1.2348 |
| Rwanda | Men | 0 | 0.9899 | 0.0026 | 0.9833 | 0.9939 | 0.4846 | 0.4814 | 0.4866 | 0.3114 | 0.5580 | 1.2482 | 1.1172 |
| Rwanda | Men | 1 | 0.0026 | 0.0011 | 0.0011 | 0.0061 | 0.0013 | 0.0005 | 0.0030 | 0.2355 | 0.4852 | 0.9654 | 0.9825 |
| Rwanda | Men | 2 | 0.0075 | 0.0021 | 0.0043 | 0.0131 | 0.0037 | 0.0021 | 0.0064 | 0.2795 | 0.5287 | 1.1117 | 1.0544 |
| Rwanda | Women | 0 | 0.9465 | 0.0062 | 0.9331 | 0.9574 | 0.4634 | 0.4568 | 0.4688 | 0.3835 | 0.6193 | 2.4106 | 1.5526 |
| Rwanda | Women | 1 | 0.0277 | 0.0034 | 0.0217 | 0.0353 | 0.0136 | 0.0106 | 0.0173 | 0.2229 | 0.4721 | 1.4420 | 1.2008 |
| Rwanda | Women | 2 | 0.0258 | 0.0038 | 0.0192 | 0.0345 | 0.0126 | 0.0094 | 0.0169 | 0.2991 | 0.5469 | 1.8258 | 1.3512 |
| Samoa | Men | 0 | 0.4712 | 0.0075 | 0.4565 | 0.4859 | 0.2307 | 0.2235 | 0.2379 | 0.0018 | 0.0423 | 0.1000 | 0.3162 |
| Samoa | Men | 1 | 0.1632 | 0.0076 | 0.1489 | 0.1787 | 0.0799 | 0.0729 | 0.0875 | 0.0034 | 0.0580 | 0.1858 | 0.4311 |
| Samoa | Men | 2 | 0.3656 | 0.0085 | 0.3491 | 0.3824 | 0.1790 | 0.1709 | 0.1872 | 0.0025 | 0.0499 | 0.1403 | 0.3746 |
| Samoa | Women | 0 | 0.2741 | 0.0204 | 0.2359 | 0.3159 | 0.1342 | 0.1155 | 0.1546 | 0.0160 | 0.1265 | 1.4728 | 1.2136 |
| Samoa | Women | 1 | 0.3309 | 0.0219 | 0.2894 | 0.3751 | 0.1620 | 0.1417 | 0.1836 | 0.0165 | 0.1284 | 1.5127 | 1.2299 |
| Samoa | Women | 2 | 0.3951 | 0.0172 | 0.3619 | 0.4293 | 0.1934 | 0.1772 | 0.2102 | 0.0094 | 0.0972 | 0.8373 | 0.9150 |
| Sao Tome and Principe | Men | 0 | 0.9201 | 0.0141 | 0.8876 | 0.9438 | 0.4505 | 0.4346 | 0.4621 | 0.0392 | 0.1981 | 1.2724 | 1.1280 |
| Sao Tome and Principe | Men | 1 | 0.0060 | 0.0036 | 0.0018 | 0.0196 | 0.0029 | 0.0009 | 0.0096 | 0.0323 | 0.1796 | 1.0034 | 1.0017 |
| Sao Tome and Principe | Men | 2 | 0.0739 | 0.0132 | 0.0518 | 0.1044 | 0.0362 | 0.0254 | 0.0511 | 0.0370 | 0.1923 | 1.1995 | 1.0952 |
| Sao Tome and Principe | Women | 0 | 0.7658 | 0.0174 | 0.7299 | 0.7983 | 0.3749 | 0.3574 | 0.3908 | 0.0585 | 0.2419 | 1.4242 | 1.1934 |
| Sao Tome and Principe | Women | 1 | 0.0206 | 0.0055 | 0.0122 | 0.0348 | 0.0101 | 0.0060 | 0.0170 | 0.0524 | 0.2289 | 1.3128 | 1.1458 |
| Sao Tome and Principe | Women | 2 | 0.2135 | 0.0178 | 0.1808 | 0.2504 | 0.1045 | 0.0885 | 0.1226 | 0.0649 | 0.2547 | 1.5752 | 1.2551 |
| Seychelles | Men | 0 | 0.8503 | 0.0154 | 0.8176 | 0.8781 | 0.4163 | 0.4003 | 0.4299 | 0.0298 | 0.1726 | 0.9766 | 0.9882 |
| Seychelles | Men | 1 | 0.0232 | 0.0071 | 0.0127 | 0.0420 | 0.0114 | 0.0062 | 0.0206 | 0.0355 | 0.1883 | 1.4224 | 1.1927 |
| Seychelles | Men | 2 | 0.1264 | 0.0141 | 0.1013 | 0.1567 | 0.0619 | 0.0496 | 0.0767 | 0.0287 | 0.1695 | 0.9134 | 0.9557 |
| Seychelles | Women | 0 | 0.6511 | 0.0187 | 0.6135 | 0.6869 | 0.3188 | 0.3004 | 0.3363 | 0.0250 | 0.1581 | 1.0141 | 1.0070 |
| Seychelles | Women | 1 | 0.0729 | 0.0107 | 0.0545 | 0.0969 | 0.0357 | 0.0267 | 0.0475 | 0.0274 | 0.1656 | 1.2534 | 1.1196 |
| Seychelles | Women | 2 | 0.2760 | 0.0173 | 0.2434 | 0.3111 | 0.1351 | 0.1192 | 0.1523 | 0.0241 | 0.1553 | 0.9445 | 0.9718 |
| Solomon Islands | Men | 0 | 0.7915 | 0.0206 | 0.7482 | 0.8290 | 0.3875 | 0.3663 | 0.4059 | 0.0830 | 0.2881 | 1.3295 | 1.1531 |
| Solomon Islands | Men | 1 | 0.0902 | 0.0140 | 0.0663 | 0.1216 | 0.0441 | 0.0324 | 0.0595 | 0.0769 | 0.2772 | 1.2183 | 1.1038 |
| Solomon Islands | Men | 2 | 0.1183 | 0.0145 | 0.0927 | 0.1499 | 0.0579 | 0.0454 | 0.0734 | 0.0651 | 0.2552 | 0.9954 | 0.9977 |
| Solomon Islands | Women | 0 | 0.6929 | 0.0236 | 0.6447 | 0.7372 | 0.3392 | 0.3156 | 0.3609 | 0.0935 | 0.3058 | 1.8810 | 1.3715 |
| Solomon Islands | Women | 1 | 0.1215 | 0.0146 | 0.0958 | 0.1531 | 0.0595 | 0.0469 | 0.0750 | 0.0706 | 0.2658 | 1.3993 | 1.1829 |
| Solomon Islands | Women | 2 | 0.1856 | 0.0179 | 0.1530 | 0.2233 | 0.0909 | 0.0749 | 0.1093 | 0.0757 | 0.2751 | 1.5166 | 1.2315 |
| Sri Lanka | Men | 0 | 0.9640 | 0.0055 | 0.9516 | 0.9733 | 0.4720 | 0.4659 | 0.4765 | 1.4435 | 1.2014 | 0.9318 | 0.9653 |
| Sri Lanka | Men | 1 | 0.0111 | 0.0031 | 0.0065 | 0.0190 | 0.0055 | 0.0032 | 0.0093 | 1.4293 | 1.1955 | 1.0101 | 1.0051 |
| Sri Lanka | Men | 2 | 0.0249 | 0.0045 | 0.0174 | 0.0355 | 0.0122 | 0.0085 | 0.0174 | 1.4238 | 1.1933 | 0.8814 | 0.9388 |
| Sri Lanka | Women | 0 | 0.9066 | 0.0078 | 0.8902 | 0.9207 | 0.4439 | 0.4359 | 0.4508 | 1.1845 | 1.0883 | 1.4880 | 1.2199 |
| Sri Lanka | Women | 1 | 0.0296 | 0.0046 | 0.0219 | 0.0400 | 0.0145 | 0.0107 | 0.0196 | 1.2104 | 1.1002 | 1.5404 | 1.2411 |
| Sri Lanka | Women | 2 | 0.0638 | 0.0063 | 0.0526 | 0.0773 | 0.0312 | 0.0257 | 0.0378 | 1.0982 | 1.0480 | 1.3721 | 1.1714 |
| Sudan | Men | 0 | 0.9299 | 0.0081 | 0.9123 | 0.9442 | 0.4553 | 0.4467 | 0.4623 | 2.6794 | 1.6369 | 1.6793 | 1.2959 |
| Sudan | Men | 1 | 0.0184 | 0.0043 | 0.0116 | 0.0290 | 0.0090 | 0.0057 | 0.0142 | 2.7103 | 1.6463 | 1.6345 | 1.2785 |
| Sudan | Men | 2 | 0.0516 | 0.0069 | 0.0397 | 0.0669 | 0.0253 | 0.0194 | 0.0328 | 2.5769 | 1.6053 | 1.6316 | 1.2773 |
| Sudan | Women | 0 | 0.8261 | 0.0123 | 0.8006 | 0.8490 | 0.4045 | 0.3920 | 0.4157 | 2.4010 | 1.5495 | 3.4071 | 1.8458 |
| Sudan | Women | 1 | 0.0454 | 0.0046 | 0.0372 | 0.0554 | 0.0222 | 0.0182 | 0.0271 | 1.1166 | 1.0567 | 1.5427 | 1.2420 |
| Sudan | Women | 2 | 0.1285 | 0.0105 | 0.1093 | 0.1505 | 0.0629 | 0.0535 | 0.0737 | 2.2268 | 1.4922 | 3.1922 | 1.7867 |
| Tajikistan | Men | 0 | 0.8728 | 0.0182 | 0.8328 | 0.9044 | 0.4273 | 0.4077 | 0.4428 | 2.2076 | 1.4858 | 2.1706 | 1.4733 |
| Tajikistan | Men | 1 | 0.0263 | 0.0079 | 0.0145 | 0.0471 | 0.0129 | 0.0071 | 0.0231 | 1.8077 | 1.3445 | 1.7498 | 1.3228 |
| Tajikistan | Men | 2 | 0.1009 | 0.0164 | 0.0729 | 0.1379 | 0.0494 | 0.0357 | 0.0675 | 2.2044 | 1.4847 | 2.1468 | 1.4652 |
| Tajikistan | Women | 0 | 0.8264 | 0.0157 | 0.7933 | 0.8551 | 0.4046 | 0.3884 | 0.4186 | 0.9679 | 0.9838 | 1.5704 | 1.2532 |
| Tajikistan | Women | 1 | 0.0375 | 0.0072 | 0.0256 | 0.0546 | 0.0184 | 0.0126 | 0.0267 | 0.8130 | 0.9016 | 1.5145 | 1.2306 |
| Tajikistan | Women | 2 | 0.1361 | 0.0125 | 0.1134 | 0.1626 | 0.0667 | 0.0555 | 0.0796 | 0.7501 | 0.8661 | 1.1373 | 1.0664 |
| Timor Leste | Men | 0 | 0.9910 | 0.0052 | 0.9726 | 0.9971 | 0.4852 | 0.4762 | 0.4882 | 0.3273 | 0.5721 | 1.1124 | 1.0547 |
| Timor Leste | Men | 1 | 0.0038 | 0.0024 | 0.0011 | 0.0133 | 0.0019 | 0.0005 | 0.0065 | 0.1736 | 0.4166 | 0.5297 | 0.7278 |
| Timor Leste | Men | 2 | 0.0052 | 0.0032 | 0.0015 | 0.0175 | 0.0026 | 0.0008 | 0.0086 | 0.2227 | 0.4719 | 0.8174 | 0.9041 |
| Timor Leste | Women | 0 | 0.9806 | 0.0090 | 0.9522 | 0.9923 | 0.4801 | 0.4662 | 0.4858 | 0.3715 | 0.6095 | 2.8036 | 1.6744 |
| Timor Leste | Women | 1 | 0.0085 | 0.0044 | 0.0031 | 0.0230 | 0.0041 | 0.0015 | 0.0113 | 0.1945 | 0.4410 | 1.5686 | 1.2525 |
| Timor Leste | Women | 2 | 0.0109 | 0.0052 | 0.0043 | 0.0275 | 0.0053 | 0.0021 | 0.0135 | 0.2159 | 0.4646 | 1.5366 | 1.2396 |
| Togo | Men | 0 | 0.9553 | 0.0141 | 0.9178 | 0.9761 | 0.4677 | 0.4494 | 0.4779 | 0.2805 | 0.5296 | 3.6032 | 1.8982 |
| Togo | Men | 1 | 0.0136 | 0.0095 | 0.0034 | 0.0525 | 0.0067 | 0.0017 | 0.0257 | 0.4055 | 0.6368 | 5.6032 | 2.3671 |
| Togo | Men | 2 | 0.0312 | 0.0110 | 0.0155 | 0.0616 | 0.0153 | 0.0076 | 0.0302 | 0.2402 | 0.4901 | 3.0260 | 1.7395 |
| Togo | Women | 0 | 0.8222 | 0.0237 | 0.7709 | 0.8640 | 0.4025 | 0.3774 | 0.4230 | 0.3017 | 0.5493 | 2.9487 | 1.7172 |
| Togo | Women | 1 | 0.0718 | 0.0168 | 0.0451 | 0.1126 | 0.0352 | 0.0221 | 0.0551 | 0.3328 | 0.5768 | 3.4085 | 1.8462 |
| Togo | Women | 2 | 0.1060 | 0.0184 | 0.0749 | 0.1479 | 0.0519 | 0.0367 | 0.0724 | 0.2812 | 0.5303 | 2.7849 | 1.6688 |
| Tokelau | Men | 0 | 0.3065 | 0.0639 | 0.1968 | 0.4435 | 0.1500 | 0.0963 | 0.2171 | 0.0013 | 0.0355 | 3.9281 | 1.9819 |
| Tokelau | Men | 1 | 0.1055 | 0.0158 | 0.0783 | 0.1407 | 0.0516 | 0.0383 | 0.0689 | 0.0002 | 0.0132 | 0.6620 | 0.8136 |
| Tokelau | Men | 2 | 0.5881 | 0.0549 | 0.4780 | 0.6900 | 0.2879 | 0.2340 | 0.3378 | 0.0008 | 0.0285 | 2.5470 | 1.5959 |
| Tokelau | Women | 0 | 0.2455 | 0.0329 | 0.1868 | 0.3156 | 0.1202 | 0.0914 | 0.1545 | 0.0004 | 0.0207 | 1.2699 | 1.1269 |
| Tokelau | Women | 1 | 0.2101 | 0.0212 | 0.1715 | 0.2546 | 0.1028 | 0.0840 | 0.1246 | 0.0002 | 0.0140 | 0.6701 | 0.8186 |
| Tokelau | Women | 2 | 0.5444 | 0.0536 | 0.4389 | 0.6461 | 0.2665 | 0.2149 | 0.3163 | 0.0008 | 0.0291 | 2.5498 | 1.5968 |
| Turkmenistan | Men | 0 | 0.8452 | 0.0128 | 0.8184 | 0.8688 | 0.4138 | 0.4007 | 0.4253 | 0.6216 | 0.7884 | 1.6987 | 1.3033 |
| Turkmenistan | Men | 1 | 0.0502 | 0.0078 | 0.0369 | 0.0679 | 0.0246 | 0.0181 | 0.0332 | 0.6313 | 0.7945 | 1.8543 | 1.3617 |
| Turkmenistan | Men | 2 | 0.1046 | 0.0104 | 0.0859 | 0.1268 | 0.0512 | 0.0421 | 0.0621 | 0.5698 | 0.7548 | 1.5069 | 1.2276 |
| Turkmenistan | Women | 0 | 0.7945 | 0.0126 | 0.7687 | 0.8182 | 0.3890 | 0.3764 | 0.4006 | 0.4460 | 0.6678 | 1.6210 | 1.2732 |
| Turkmenistan | Women | 1 | 0.0617 | 0.0076 | 0.0484 | 0.0784 | 0.0302 | 0.0237 | 0.0384 | 0.4554 | 0.6749 | 1.7117 | 1.3083 |
| Turkmenistan | Women | 2 | 0.1438 | 0.0106 | 0.1242 | 0.1658 | 0.0704 | 0.0608 | 0.0812 | 0.4185 | 0.6469 | 1.4763 | 1.2151 |
| Tuvalu | Men | 0 | 0.4126 | 0.0371 | 0.3423 | 0.4867 | 0.2020 | 0.1676 | 0.2383 | 0.0038 | 0.0617 | 2.0796 | 1.4421 |
| Tuvalu | Men | 1 | 0.1717 | 0.0163 | 0.1422 | 0.2060 | 0.0841 | 0.0696 | 0.1009 | 0.0012 | 0.0353 | 0.6328 | 0.7955 |
| Tuvalu | Men | 2 | 0.4156 | 0.0252 | 0.3673 | 0.4657 | 0.2035 | 0.1798 | 0.2280 | 0.0017 | 0.0418 | 0.9365 | 0.9677 |
| Tuvalu | Women | 0 | 0.2124 | 0.0207 | 0.1746 | 0.2558 | 0.1040 | 0.0855 | 0.1252 | 0.0015 | 0.0382 | 1.0461 | 1.0228 |
| Tuvalu | Women | 1 | 0.2197 | 0.0207 | 0.1818 | 0.2629 | 0.1076 | 0.0890 | 0.1287 | 0.0014 | 0.0378 | 1.1575 | 1.0759 |
| Tuvalu | Women | 2 | 0.5680 | 0.0284 | 0.5116 | 0.6227 | 0.2781 | 0.2505 | 0.3049 | 0.0019 | 0.0434 | 1.4282 | 1.1951 |
| Uganda | Men | 0 | 0.9723 | 0.0068 | 0.9552 | 0.9830 | 0.4760 | 0.4677 | 0.4813 | 2.6331 | 1.6227 | 1.8113 | 1.3458 |
| Uganda | Men | 1 | 0.0079 | 0.0033 | 0.0034 | 0.0179 | 0.0039 | 0.0017 | 0.0088 | 2.1209 | 1.4563 | 1.4504 | 1.2043 |
| Uganda | Men | 2 | 0.0199 | 0.0061 | 0.0108 | 0.0362 | 0.0097 | 0.0053 | 0.0177 | 2.9239 | 1.7099 | 2.0165 | 1.4200 |
| Uganda | Women | 0 | 0.9029 | 0.0119 | 0.8769 | 0.9239 | 0.4420 | 0.4293 | 0.4523 | 2.3461 | 1.5317 | 2.2230 | 1.4910 |
| Uganda | Women | 1 | 0.0386 | 0.0074 | 0.0264 | 0.0559 | 0.0189 | 0.0129 | 0.0274 | 2.1218 | 1.4567 | 1.9505 | 1.3966 |
| Uganda | Women | 2 | 0.0586 | 0.0093 | 0.0428 | 0.0796 | 0.0287 | 0.0210 | 0.0390 | 2.2589 | 1.5030 | 2.1730 | 1.4741 |
| United Republic of Tanzania | Men | 0 | 0.9763 | 0.0077 | 0.9554 | 0.9875 | 0.4780 | 0.4677 | 0.4835 | 1.2313 | 1.1096 | 1.3479 | 1.1610 |
| United Republic of Tanzania | Men | 1 | 0.0064 | 0.0037 | 0.0020 | 0.0200 | 0.0031 | 0.0010 | 0.0098 | 1.0530 | 1.0262 | 1.1871 | 1.0896 |
| United Republic of Tanzania | Men | 2 | 0.0173 | 0.0060 | 0.0088 | 0.0339 | 0.0085 | 0.0043 | 0.0166 | 0.9999 | 0.9999 | 1.0765 | 1.0375 |
| United Republic of Tanzania | Women | 0 | 0.8173 | 0.0285 | 0.7546 | 0.8668 | 0.4001 | 0.3695 | 0.4244 | 3.3235 | 1.8231 | 7.2492 | 2.6924 |
| United Republic of Tanzania | Women | 1 | 0.0651 | 0.0190 | 0.0364 | 0.1137 | 0.0318 | 0.0178 | 0.0557 | 3.6125 | 1.9007 | 9.6214 | 3.1018 |
| United Republic of Tanzania | Women | 2 | 0.1177 | 0.0191 | 0.0850 | 0.1607 | 0.0576 | 0.0416 | 0.0787 | 2.1508 | 1.4666 | 4.4995 | 2.1212 |
| Vietnam | Men | 0 | 0.9825 | 0.0053 | 0.9685 | 0.9904 | 0.4811 | 0.4742 | 0.4849 | 15.7060 | 3.9631 | 1.8739 | 1.3689 |
| Vietnam | Men | 1 | 0.0066 | 0.0031 | 0.0026 | 0.0167 | 0.0033 | 0.0013 | 0.0082 | 14.2982 | 3.7813 | 2.3331 | 1.5275 |
| Vietnam | Men | 2 | 0.0108 | 0.0044 | 0.0049 | 0.0238 | 0.0053 | 0.0024 | 0.0116 | 17.1250 | 4.1382 | 1.7567 | 1.3254 |
| Vietnam | Women | 0 | 0.9839 | 0.0033 | 0.9759 | 0.9893 | 0.4817 | 0.4778 | 0.4844 | 6.9681 | 2.6397 | 0.8544 | 0.9243 |
| Vietnam | Women | 1 | 0.0059 | 0.0022 | 0.0028 | 0.0123 | 0.0029 | 0.0014 | 0.0060 | 8.2486 | 2.8720 | 1.0720 | 1.0354 |
| Vietnam | Women | 2 | 0.0102 | 0.0025 | 0.0062 | 0.0166 | 0.0050 | 0.0031 | 0.0081 | 6.3569 | 2.5213 | 0.7530 | 0.8678 |
| Zambia | Men | 0 | 0.9668 | 0.0079 | 0.9474 | 0.9792 | 0.4733 | 0.4638 | 0.4794 | 1.7793 | 1.3339 | 1.8968 | 1.3773 |
| Zambia | Men | 1 | 0.0086 | 0.0031 | 0.0042 | 0.0173 | 0.0042 | 0.0021 | 0.0085 | 1.0412 | 1.0204 | 0.9562 | 0.9779 |
| Zambia | Men | 2 | 0.0246 | 0.0072 | 0.0139 | 0.0433 | 0.0121 | 0.0068 | 0.0212 | 1.9631 | 1.4011 | 2.2166 | 1.4888 |
| Zambia | Women | 0 | 0.8683 | 0.0121 | 0.8427 | 0.8903 | 0.4251 | 0.4126 | 0.4359 | 1.1673 | 1.0804 | 2.0252 | 1.4231 |
| Zambia | Women | 1 | 0.0568 | 0.0078 | 0.0432 | 0.0742 | 0.0278 | 0.0212 | 0.0363 | 1.0413 | 1.0205 | 2.0251 | 1.4231 |
| Zambia | Women | 2 | 0.0749 | 0.0093 | 0.0586 | 0.0953 | 0.0367 | 0.0287 | 0.0466 | 1.1304 | 1.0632 | 1.8399 | 1.3564 |
|  |  |  |  |  |  |  |  |  |  |  |  |  |  |
|  |  |  |  |  |  |  |  |  |  |  |  |  |  |
|  |  |  |  |  |  |  |  |  |  |  |  |  |  |
| **Country** | **Sex** | **BMI-only obesity** | **Prevalence** | **SE** | **Lower 95% CI** | **Upper 95% CI** | **Prevalence** | **Lower 95% CI** | **Upper 95% CI** | **DEFF** | **DEFT** | **MEFF** | **MEFT** |
| Afghanistan | Men | 0 (BMI <18.5) | 0.0454 | 0.0156 | 0.0230 | 0.0879 | 0.0222 | 0.0112 | 0.0430 | 12.1521 | 3.4860 | 9.0413 | 3.0069 |
| Afghanistan | Men | 1 (18.5-24.99) | 0.4784 | 0.0221 | 0.4353 | 0.5219 | 0.2342 | 0.2131 | 0.2555 | 4.2536 | 2.0624 | 2.4408 | 1.5623 |
| Afghanistan | Men | 2 (25.0-29.99) | 0.3346 | 0.0225 | 0.2920 | 0.3801 | 0.1638 | 0.1430 | 0.1861 | 4.9343 | 2.2213 | 2.8010 | 1.6736 |
| Afghanistan | Men | 3 (30+) | 0.1415 | 0.0155 | 0.1138 | 0.1746 | 0.0693 | 0.0557 | 0.0855 | 4.2639 | 2.0649 | 2.3219 | 1.5238 |
| Afghanistan | Women | 0 | 0.0846 | 0.0157 | 0.0585 | 0.1208 | 0.0414 | 0.0286 | 0.0592 | 6.0369 | 2.4570 | 3.5914 | 1.8951 |
| Afghanistan | Women | 1 | 0.3737 | 0.0267 | 0.3230 | 0.4273 | 0.1830 | 0.1582 | 0.2092 | 5.7778 | 2.4037 | 3.4330 | 1.8528 |
| Afghanistan | Women | 2 | 0.2794 | 0.0221 | 0.2382 | 0.3248 | 0.1368 | 0.1166 | 0.1590 | 4.6145 | 2.1481 | 2.6187 | 1.6182 |
| Afghanistan | Women | 3 | 0.2623 | 0.0284 | 0.2104 | 0.3217 | 0.1284 | 0.1030 | 0.1575 | 7.9341 | 2.8168 | 4.7059 | 2.1693 |
| Algeria | Men | 0 | 0.0255 | 0.0036 | 0.0193 | 0.0335 | 0.0125 | 0.0095 | 0.0164 | 1.9661 | 1.4022 | 1.1693 | 1.0813 |
| Algeria | Men | 1 | 0.4261 | 0.0129 | 0.4012 | 0.4515 | 0.2086 | 0.1964 | 0.2211 | 2.5743 | 1.6045 | 1.4652 | 1.2104 |
| Algeria | Men | 2 | 0.3850 | 0.0119 | 0.3620 | 0.4085 | 0.1885 | 0.1772 | 0.2000 | 2.2708 | 1.5069 | 1.2729 | 1.1282 |
| Algeria | Men | 3 | 0.1634 | 0.0090 | 0.1465 | 0.1819 | 0.0800 | 0.0717 | 0.0891 | 2.2656 | 1.5052 | 1.2515 | 1.1187 |
| Algeria | Women | 0 | 0.0192 | 0.0028 | 0.0144 | 0.0256 | 0.0094 | 0.0070 | 0.0125 | 1.5199 | 1.2329 | 1.1564 | 1.0754 |
| Algeria | Women | 1 | 0.2812 | 0.0103 | 0.2615 | 0.3018 | 0.1377 | 0.1281 | 0.1478 | 1.8857 | 1.3732 | 1.4248 | 1.1936 |
| Algeria | Women | 2 | 0.3520 | 0.0093 | 0.3340 | 0.3705 | 0.1724 | 0.1635 | 0.1814 | 1.3675 | 1.1694 | 0.9882 | 0.9941 |
| Algeria | Women | 3 | 0.3475 | 0.0103 | 0.3277 | 0.3679 | 0.1701 | 0.1604 | 0.1801 | 1.6783 | 1.2955 | 1.1965 | 1.0939 |
| American Samoa | Men | 0 | 0.0006 | 0.0006 | 0.0001 | 0.0040 | 0.0003 | 0.0000 | 0.0020 | 0.0012 | 0.0351 | 0.1080 | 0.3286 |
| American Samoa | Men | 1 | 0.0494 | 0.0113 | 0.0314 | 0.0770 | 0.0242 | 0.0154 | 0.0377 | 0.0059 | 0.0770 | 1.4000 | 1.1832 |
| American Samoa | Men | 2 | 0.2083 | 0.0183 | 0.1746 | 0.2465 | 0.1020 | 0.0855 | 0.1207 | 0.0044 | 0.0666 | 1.2488 | 1.1175 |
| American Samoa | Men | 3 | 0.7417 | 0.0116 | 0.7184 | 0.7638 | 0.3632 | 0.3517 | 0.3739 | 0.0015 | 0.0390 | 0.4122 | 0.6421 |
| American Samoa | Women | 0 | 0.0026 | 0.0027 | 0.0004 | 0.0188 | 0.0013 | 0.0002 | 0.0092 | 0.0063 | 0.0792 | 1.8804 | 1.3713 |
| American Samoa | Women | 1 | 0.0531 | 0.0106 | 0.0358 | 0.0780 | 0.0260 | 0.0175 | 0.0382 | 0.0052 | 0.0719 | 1.9353 | 1.3911 |
| American Samoa | Women | 2 | 0.1311 | 0.0067 | 0.1185 | 0.1448 | 0.0642 | 0.0580 | 0.0709 | 0.0009 | 0.0304 | 0.3030 | 0.5504 |
| American Samoa | Women | 3 | 0.8132 | 0.0142 | 0.7838 | 0.8395 | 0.3982 | 0.3838 | 0.4110 | 0.0031 | 0.0555 | 1.0372 | 1.0184 |
| Armenia | Men | 0 | 0.0396 | 0.0156 | 0.0181 | 0.0844 | 0.0194 | 0.0089 | 0.0413 | 1.8637 | 1.3652 | 2.5321 | 1.5913 |
| Armenia | Men | 1 | 0.4909 | 0.0360 | 0.4210 | 0.5612 | 0.2404 | 0.2061 | 0.2748 | 1.5108 | 1.2291 | 1.6784 | 1.2955 |
| Armenia | Men | 2 | 0.3108 | 0.0307 | 0.2541 | 0.3738 | 0.1522 | 0.1244 | 0.1830 | 1.2781 | 1.1305 | 1.3343 | 1.1551 |
| Armenia | Men | 3 | 0.1587 | 0.0224 | 0.1195 | 0.2077 | 0.0777 | 0.0585 | 0.1017 | 1.0978 | 1.0478 | 0.9806 | 0.9903 |
| Armenia | Women | 0 | 0.0444 | 0.0103 | 0.0280 | 0.0697 | 0.0217 | 0.0137 | 0.0341 | 0.6051 | 0.7779 | 3.2215 | 1.7949 |
| Armenia | Women | 1 | 0.3885 | 0.0198 | 0.3505 | 0.4279 | 0.1902 | 0.1716 | 0.2095 | 0.3954 | 0.6288 | 1.5695 | 1.2528 |
| Armenia | Women | 2 | 0.2765 | 0.0181 | 0.2423 | 0.3134 | 0.1354 | 0.1186 | 0.1535 | 0.3956 | 0.6290 | 1.4255 | 1.1939 |
| Armenia | Women | 3 | 0.2907 | 0.0181 | 0.2565 | 0.3273 | 0.1423 | 0.1256 | 0.1602 | 0.3805 | 0.6168 | 1.3364 | 1.1560 |
| Azerbaijan | Men | 0 | 0.0167 | 0.0053 | 0.0089 | 0.0311 | 0.0082 | 0.0043 | 0.0152 | 1.8000 | 1.3416 | 1.6041 | 1.2665 |
| Azerbaijan | Men | 1 | 0.3828 | 0.0213 | 0.3420 | 0.4254 | 0.1874 | 0.1675 | 0.2083 | 1.9920 | 1.4114 | 1.7090 | 1.3073 |
| Azerbaijan | Men | 2 | 0.4403 | 0.0237 | 0.3945 | 0.4870 | 0.2155 | 0.1932 | 0.2384 | 2.3537 | 1.5342 | 1.9288 | 1.3888 |
| Azerbaijan | Men | 3 | 0.1602 | 0.0144 | 0.1340 | 0.1905 | 0.0785 | 0.0656 | 0.0933 | 1.5980 | 1.2641 | 1.0910 | 1.0445 |
| Azerbaijan | Women | 0 | 0.0216 | 0.0057 | 0.0129 | 0.0360 | 0.0106 | 0.0063 | 0.0176 | 1.5708 | 1.2533 | 2.3110 | 1.5202 |
| Azerbaijan | Women | 1 | 0.3355 | 0.0197 | 0.2981 | 0.3751 | 0.1643 | 0.1460 | 0.1837 | 1.7913 | 1.3384 | 2.3658 | 1.5381 |
| Azerbaijan | Women | 2 | 0.3496 | 0.0184 | 0.3144 | 0.3864 | 0.1712 | 0.1540 | 0.1892 | 1.5341 | 1.2386 | 1.7703 | 1.3305 |
| Azerbaijan | Women | 3 | 0.2933 | 0.0178 | 0.2597 | 0.3293 | 0.1436 | 0.1271 | 0.1612 | 1.5740 | 1.2546 | 1.7103 | 1.3078 |
| Bangladesh | Men | 0 | 0.1504 | 0.0097 | 0.1323 | 0.1704 | 0.0736 | 0.0648 | 0.0835 | 12.6774 | 3.5605 | 2.0833 | 1.4434 |
| Bangladesh | Men | 1 | 0.6406 | 0.0127 | 0.6153 | 0.6653 | 0.3137 | 0.3012 | 0.3257 | 12.1337 | 3.4833 | 1.9262 | 1.3879 |
| Bangladesh | Men | 2 | 0.1842 | 0.0109 | 0.1637 | 0.2066 | 0.0902 | 0.0802 | 0.1012 | 13.6203 | 3.6906 | 2.1069 | 1.4515 |
| Bangladesh | Men | 3 | 0.0248 | 0.0037 | 0.0185 | 0.0331 | 0.0121 | 0.0091 | 0.0162 | 9.5292 | 3.0869 | 1.1657 | 1.0797 |
| Bangladesh | Women | 0 | 0.1186 | 0.0083 | 0.1032 | 0.1359 | 0.0580 | 0.0505 | 0.0665 | 13.1315 | 3.6237 | 2.2310 | 1.4937 |
| Bangladesh | Women | 1 | 0.5125 | 0.0122 | 0.4885 | 0.5365 | 0.2509 | 0.2392 | 0.2627 | 11.8781 | 3.4465 | 1.8941 | 1.3763 |
| Bangladesh | Women | 2 | 0.2783 | 0.0119 | 0.2555 | 0.3023 | 0.1363 | 0.1251 | 0.1480 | 14.0333 | 3.7461 | 2.2216 | 1.4905 |
| Bangladesh | Women | 3 | 0.0906 | 0.0067 | 0.0783 | 0.1046 | 0.0444 | 0.0383 | 0.0512 | 10.7519 | 3.2790 | 1.5090 | 1.2284 |
| Belarus | Men | 0 | 0.0110 | 0.0039 | 0.0054 | 0.0221 | 0.0054 | 0.0027 | 0.0108 | 1.4202 | 1.1917 | 2.9205 | 1.7089 |
| Belarus | Men | 1 | 0.3415 | 0.0155 | 0.3118 | 0.3726 | 0.1672 | 0.1527 | 0.1824 | 1.0685 | 1.0337 | 1.8145 | 1.3470 |
| Belarus | Men | 2 | 0.4278 | 0.0142 | 0.4002 | 0.4559 | 0.2095 | 0.1959 | 0.2232 | 0.8251 | 0.9083 | 1.3854 | 1.1770 |
| Belarus | Men | 3 | 0.2196 | 0.0130 | 0.1953 | 0.2461 | 0.1075 | 0.0956 | 0.1205 | 0.9765 | 0.9882 | 1.5848 | 1.2589 |
| Belarus | Women | 0 | 0.0212 | 0.0040 | 0.0146 | 0.0306 | 0.0104 | 0.0072 | 0.0150 | 0.8148 | 0.9026 | 2.2058 | 1.4852 |
| Belarus | Women | 1 | 0.3433 | 0.0136 | 0.3172 | 0.3705 | 0.1681 | 0.1553 | 0.1814 | 0.8718 | 0.9337 | 1.9502 | 1.3965 |
| Belarus | Women | 2 | 0.3094 | 0.0128 | 0.2848 | 0.3351 | 0.1515 | 0.1394 | 0.1641 | 0.8228 | 0.9071 | 1.7404 | 1.3192 |
| Belarus | Women | 3 | 0.3261 | 0.0136 | 0.3000 | 0.3533 | 0.1596 | 0.1469 | 0.1730 | 0.8991 | 0.9482 | 1.8513 | 1.3606 |
| Benin | Men | 0 | 0.0915 | 0.0207 | 0.0582 | 0.1408 | 0.0448 | 0.0285 | 0.0690 | 1.5889 | 1.2605 | 10.4907 | 3.2389 |
| Benin | Men | 1 | 0.6857 | 0.0197 | 0.6458 | 0.7230 | 0.3357 | 0.3162 | 0.3540 | 0.5579 | 0.7469 | 3.2157 | 1.7932 |
| Benin | Men | 2 | 0.1709 | 0.0129 | 0.1471 | 0.1976 | 0.0837 | 0.0720 | 0.0968 | 0.3616 | 0.6013 | 2.0097 | 1.4176 |
| Benin | Men | 3 | 0.0520 | 0.0083 | 0.0378 | 0.0710 | 0.0254 | 0.0185 | 0.0348 | 0.4377 | 0.6616 | 2.2355 | 1.4952 |
| Benin | Women | 0 | 0.0650 | 0.0127 | 0.0441 | 0.0949 | 0.0318 | 0.0216 | 0.0464 | 0.9702 | 0.9850 | 4.2409 | 2.0594 |
| Benin | Women | 1 | 0.6482 | 0.0547 | 0.5352 | 0.7468 | 0.3174 | 0.2620 | 0.3656 | 4.7740 | 2.1850 | 23.8882 | 4.8876 |
| Benin | Women | 2 | 0.1723 | 0.0282 | 0.1238 | 0.2347 | 0.0844 | 0.0606 | 0.1149 | 2.0302 | 1.4248 | 9.0320 | 3.0053 |
| Benin | Women | 3 | 0.1145 | 0.0189 | 0.0823 | 0.1571 | 0.0560 | 0.0403 | 0.0769 | 1.2831 | 1.1328 | 5.4969 | 2.3445 |
| Bhutan | Men | 0 | 0.0163 | 0.0030 | 0.0113 | 0.0234 | 0.0080 | 0.0055 | 0.0115 | 0.0287 | 0.1694 | 0.7936 | 0.8908 |
| Bhutan | Men | 1 | 0.4847 | 0.0133 | 0.4582 | 0.5112 | 0.2373 | 0.2244 | 0.2503 | 0.0368 | 0.1919 | 1.2257 | 1.1071 |
| Bhutan | Men | 2 | 0.3976 | 0.0141 | 0.3700 | 0.4259 | 0.1947 | 0.1812 | 0.2085 | 0.0428 | 0.2069 | 1.4359 | 1.1983 |
| Bhutan | Men | 3 | 0.1014 | 0.0084 | 0.0859 | 0.1194 | 0.0497 | 0.0420 | 0.0585 | 0.0403 | 0.2008 | 1.3165 | 1.1474 |
| Bhutan | Women | 0 | 0.0222 | 0.0035 | 0.0163 | 0.0303 | 0.0109 | 0.0080 | 0.0148 | 0.0218 | 0.1477 | 1.5025 | 1.2258 |
| Bhutan | Women | 1 | 0.3935 | 0.0125 | 0.3690 | 0.4186 | 0.1927 | 0.1806 | 0.2049 | 0.0258 | 0.1606 | 1.7866 | 1.3366 |
| Bhutan | Women | 2 | 0.3992 | 0.0108 | 0.3779 | 0.4209 | 0.1954 | 0.1850 | 0.2061 | 0.0193 | 0.1388 | 1.3308 | 1.1536 |
| Bhutan | Women | 3 | 0.1851 | 0.0096 | 0.1668 | 0.2049 | 0.0906 | 0.0817 | 0.1003 | 0.0240 | 0.1551 | 1.6675 | 1.2913 |
| Botswana | Men | 0 | 0.1559 | 0.0181 | 0.1237 | 0.1947 | 0.0763 | 0.0606 | 0.0953 | 0.1953 | 0.4419 | 1.8411 | 1.3569 |
| Botswana | Men | 1 | 0.5925 | 0.0258 | 0.5411 | 0.6419 | 0.2901 | 0.2649 | 0.3143 | 0.2174 | 0.4663 | 2.0291 | 1.4245 |
| Botswana | Men | 2 | 0.1809 | 0.0181 | 0.1480 | 0.2193 | 0.0886 | 0.0725 | 0.1073 | 0.1753 | 0.4187 | 1.5497 | 1.2449 |
| Botswana | Men | 3 | 0.0707 | 0.0127 | 0.0495 | 0.1001 | 0.0346 | 0.0242 | 0.0490 | 0.1940 | 0.4404 | 1.6700 | 1.2923 |
| Botswana | Women | 0 | 0.0801 | 0.0116 | 0.0602 | 0.1060 | 0.0392 | 0.0295 | 0.0519 | 0.1383 | 0.3719 | 3.6305 | 1.9054 |
| Botswana | Women | 1 | 0.4208 | 0.0174 | 0.3872 | 0.4552 | 0.2060 | 0.1896 | 0.2229 | 0.0943 | 0.3071 | 2.0831 | 1.4433 |
| Botswana | Women | 2 | 0.2638 | 0.0164 | 0.2330 | 0.2972 | 0.1292 | 0.1141 | 0.1455 | 0.1053 | 0.3246 | 2.2069 | 1.4856 |
| Botswana | Women | 3 | 0.2352 | 0.0142 | 0.2085 | 0.2643 | 0.1152 | 0.1021 | 0.1294 | 0.0857 | 0.2928 | 1.7133 | 1.3089 |
| Brunei Darussalam | Men | 0 | 0.0094 | 0.0068 | 0.0022 | 0.0385 | 0.0046 | 0.0011 | 0.0188 | 0.0670 | 0.2588 | 5.2942 | 2.3009 |
| Brunei Darussalam | Men | 1 | 0.2793 | 0.0232 | 0.2361 | 0.3271 | 0.1368 | 0.1156 | 0.1601 | 0.0358 | 0.1891 | 1.5618 | 1.2497 |
| Brunei Darussalam | Men | 2 | 0.4057 | 0.0224 | 0.3626 | 0.4503 | 0.1986 | 0.1775 | 0.2205 | 0.0278 | 0.1668 | 1.1904 | 1.0911 |
| Brunei Darussalam | Men | 3 | 0.3056 | 0.0238 | 0.2610 | 0.3541 | 0.1496 | 0.1278 | 0.1734 | 0.0356 | 0.1886 | 1.6185 | 1.2722 |
| Brunei Darussalam | Women | 0 | 0.0354 | 0.0082 | 0.0224 | 0.0555 | 0.0173 | 0.0110 | 0.0272 | 0.0260 | 0.1611 | 2.0337 | 1.4261 |
| Brunei Darussalam | Women | 1 | 0.2391 | 0.0204 | 0.2013 | 0.2813 | 0.1170 | 0.0986 | 0.1377 | 0.0302 | 0.1739 | 1.8446 | 1.3582 |
| Brunei Darussalam | Women | 2 | 0.3672 | 0.0198 | 0.3294 | 0.4067 | 0.1798 | 0.1613 | 0.1991 | 0.0222 | 0.1489 | 1.3630 | 1.1675 |
| Brunei Darussalam | Women | 3 | 0.3584 | 0.0219 | 0.3167 | 0.4023 | 0.1755 | 0.1551 | 0.1970 | 0.0274 | 0.1657 | 1.7403 | 1.3192 |
| Cabo Verde | Men | 0 | 0.0254 | 0.0068 | 0.0150 | 0.0426 | 0.0124 | 0.0074 | 0.0209 | 0.0883 | 0.2972 | 1.0787 | 1.0386 |
| Cabo Verde | Men | 1 | 0.5451 | 0.0286 | 0.4887 | 0.6003 | 0.2669 | 0.2393 | 0.2939 | 0.1573 | 0.3967 | 2.1750 | 1.4748 |
| Cabo Verde | Men | 2 | 0.3469 | 0.0268 | 0.2965 | 0.4011 | 0.1699 | 0.1452 | 0.1964 | 0.1511 | 0.3888 | 2.1511 | 1.4667 |
| Cabo Verde | Men | 3 | 0.0826 | 0.0151 | 0.0574 | 0.1174 | 0.0404 | 0.0281 | 0.0575 | 0.1436 | 0.3789 | 1.7375 | 1.3182 |
| Cabo Verde | Women | 0 | 0.0212 | 0.0061 | 0.0120 | 0.0372 | 0.0104 | 0.0059 | 0.0182 | 0.0934 | 0.3056 | 2.0121 | 1.4185 |
| Cabo Verde | Women | 1 | 0.3716 | 0.0282 | 0.3182 | 0.4284 | 0.1819 | 0.1558 | 0.2098 | 0.1763 | 0.4199 | 4.0328 | 2.0082 |
| Cabo Verde | Women | 2 | 0.3415 | 0.0208 | 0.3020 | 0.3834 | 0.1672 | 0.1478 | 0.1877 | 0.0994 | 0.3153 | 2.0594 | 1.4351 |
| Cabo Verde | Women | 3 | 0.2657 | 0.0236 | 0.2221 | 0.3143 | 0.1301 | 0.1087 | 0.1539 | 0.1469 | 0.3833 | 3.1948 | 1.7874 |
| Cambodia | Men | 0 | 0.0983 | 0.0081 | 0.0836 | 0.1154 | 0.0481 | 0.0409 | 0.0565 | 0.6896 | 0.8305 | 1.2965 | 1.1386 |
| Cambodia | Men | 1 | 0.7872 | 0.0119 | 0.7630 | 0.8095 | 0.3854 | 0.3736 | 0.3963 | 0.7843 | 0.8856 | 1.4453 | 1.2022 |
| Cambodia | Men | 2 | 0.1042 | 0.0090 | 0.0878 | 0.1231 | 0.0510 | 0.0430 | 0.0603 | 0.8067 | 0.8981 | 1.4664 | 1.2110 |
| Cambodia | Men | 3 | 0.0103 | 0.0024 | 0.0065 | 0.0163 | 0.0051 | 0.0032 | 0.0080 | 0.5336 | 0.7305 | 0.7862 | 0.8867 |
| Cambodia | Women | 0 | 0.1505 | 0.0085 | 0.1347 | 0.1679 | 0.0737 | 0.0660 | 0.0822 | 0.5393 | 0.7343 | 1.7537 | 1.3243 |
| Cambodia | Women | 1 | 0.6573 | 0.0103 | 0.6368 | 0.6772 | 0.3218 | 0.3118 | 0.3316 | 0.4550 | 0.6746 | 1.4868 | 1.2194 |
| Cambodia | Women | 2 | 0.1649 | 0.0086 | 0.1486 | 0.1825 | 0.0807 | 0.0728 | 0.0894 | 0.5220 | 0.7225 | 1.6647 | 1.2902 |
| Cambodia | Women | 3 | 0.0273 | 0.0035 | 0.0211 | 0.0351 | 0.0133 | 0.0103 | 0.0172 | 0.4540 | 0.6738 | 1.4071 | 1.1862 |
| Comoros | Men | 0 | 0.0595 | 0.0163 | 0.0344 | 0.1008 | 0.0291 | 0.0168 | 0.0494 | 0.0443 | 0.2105 | 1.8725 | 1.3684 |
| Comoros | Men | 1 | 0.5671 | 0.0291 | 0.5094 | 0.6231 | 0.2777 | 0.2494 | 0.3051 | 0.0320 | 0.1789 | 1.1263 | 1.0613 |
| Comoros | Men | 2 | 0.2980 | 0.0283 | 0.2456 | 0.3564 | 0.1459 | 0.1202 | 0.1745 | 0.0356 | 0.1887 | 1.2340 | 1.1109 |
| Comoros | Men | 3 | 0.0754 | 0.0159 | 0.0496 | 0.1131 | 0.0369 | 0.0243 | 0.0554 | 0.0336 | 0.1833 | 1.1661 | 1.0799 |
| Comoros | Women | 0 | 0.0327 | 0.0067 | 0.0218 | 0.0488 | 0.0160 | 0.0107 | 0.0239 | 0.0145 | 0.1202 | 1.6999 | 1.3038 |
| Comoros | Women | 1 | 0.3551 | 0.0177 | 0.3213 | 0.3904 | 0.1739 | 0.1573 | 0.1912 | 0.0138 | 0.1173 | 1.4773 | 1.2154 |
| Comoros | Women | 2 | 0.2983 | 0.0156 | 0.2686 | 0.3298 | 0.1461 | 0.1315 | 0.1615 | 0.0118 | 0.1084 | 1.2816 | 1.1321 |
| Comoros | Women | 3 | 0.3138 | 0.0164 | 0.2826 | 0.3469 | 0.1537 | 0.1384 | 0.1698 | 0.0126 | 0.1124 | 1.3773 | 1.1736 |
| Cook Islands | Men | 0 | 0.0070 | 0.0025 | 0.0035 | 0.0140 | 0.0034 | 0.0017 | 0.0068 | 0.0003 | 0.0159 | 0.2448 | 0.4948 |
| Cook Islands | Men | 1 | 0.1023 | 0.0143 | 0.0775 | 0.1339 | 0.0501 | 0.0379 | 0.0655 | 0.0006 | 0.0252 | 0.6612 | 0.8131 |
| Cook Islands | Men | 2 | 0.1982 | 0.0123 | 0.1752 | 0.2234 | 0.0971 | 0.0858 | 0.1094 | 0.0003 | 0.0165 | 0.2446 | 0.4946 |
| Cook Islands | Men | 3 | 0.6924 | 0.0158 | 0.6607 | 0.7225 | 0.3390 | 0.3235 | 0.3537 | 0.0003 | 0.0183 | 0.3176 | 0.5635 |
| Cook Islands | Women | 0 | 0.0000 | 0.0000 | 0.0000 | 0.0000 | 0.0000 | 0.0000 | 0.0000 | 0.0000 | 0.0000 | 0.0000 | 0.0000 |
| Cook Islands | Women | 1 | 0.0828 | 0.0072 | 0.0697 | 0.0981 | 0.0405 | 0.0341 | 0.0480 | 0.0002 | 0.0155 | 0.2124 | 0.4608 |
| Cook Islands | Women | 2 | 0.1810 | 0.0147 | 0.1540 | 0.2116 | 0.0886 | 0.0754 | 0.1036 | 0.0005 | 0.0226 | 0.4382 | 0.6620 |
| Cook Islands | Women | 3 | 0.7362 | 0.0163 | 0.7031 | 0.7668 | 0.3604 | 0.3442 | 0.3754 | 0.0005 | 0.0218 | 0.4225 | 0.6500 |
| Ecuador | Men | 0 | 0.0088 | 0.0029 | 0.0046 | 0.0167 | 0.0043 | 0.0023 | 0.0082 | 1.3263 | 1.1516 | 1.1254 | 1.0608 |
| Ecuador | Men | 1 | 0.3160 | 0.0149 | 0.2876 | 0.3459 | 0.1547 | 0.1408 | 0.1693 | 1.4227 | 1.1928 | 1.2974 | 1.1390 |
| Ecuador | Men | 2 | 0.4542 | 0.0178 | 0.4195 | 0.4893 | 0.2224 | 0.2054 | 0.2396 | 1.7857 | 1.3363 | 1.6275 | 1.2757 |
| Ecuador | Men | 3 | 0.2210 | 0.0167 | 0.1899 | 0.2555 | 0.1082 | 0.0930 | 0.1251 | 2.2654 | 1.5051 | 2.0359 | 1.4268 |
| Ecuador | Women | 0 | 0.0073 | 0.0023 | 0.0039 | 0.0136 | 0.0036 | 0.0019 | 0.0067 | 1.1317 | 1.0638 | 1.2086 | 1.0994 |
| Ecuador | Women | 1 | 0.2490 | 0.0152 | 0.2205 | 0.2799 | 0.1219 | 0.1080 | 0.1371 | 1.8498 | 1.3601 | 2.2277 | 1.4926 |
| Ecuador | Women | 2 | 0.3972 | 0.0151 | 0.3680 | 0.4272 | 0.1945 | 0.1802 | 0.2092 | 1.4358 | 1.1982 | 1.7306 | 1.3155 |
| Ecuador | Women | 3 | 0.3464 | 0.0148 | 0.3180 | 0.3760 | 0.1696 | 0.1557 | 0.1841 | 1.4556 | 1.2065 | 1.7671 | 1.3293 |
| Eritrea | Men | 0 | 0.3410 | 0.0199 | 0.3032 | 0.3809 | 0.1670 | 0.1485 | 0.1865 | 0.1199 | 0.3463 | 1.9003 | 1.3785 |
| Eritrea | Men | 1 | 0.5543 | 0.0164 | 0.5220 | 0.5863 | 0.2714 | 0.2555 | 0.2870 | 0.0747 | 0.2733 | 1.1915 | 1.0915 |
| Eritrea | Men | 2 | 0.0958 | 0.0119 | 0.0750 | 0.1217 | 0.0469 | 0.0367 | 0.0596 | 0.1110 | 0.3332 | 1.8029 | 1.3427 |
| Eritrea | Men | 3 | 0.0088 | 0.0033 | 0.0043 | 0.0181 | 0.0043 | 0.0021 | 0.0089 | 0.0827 | 0.2876 | 1.2760 | 1.1296 |
| Eritrea | Women | 0 | 0.3634 | 0.0158 | 0.3331 | 0.3948 | 0.1779 | 0.1631 | 0.1933 | 0.4131 | 0.6427 | 3.4664 | 1.8618 |
| Eritrea | Women | 1 | 0.5019 | 0.0125 | 0.4774 | 0.5265 | 0.2458 | 0.2337 | 0.2578 | 0.2410 | 0.4909 | 2.0395 | 1.4281 |
| Eritrea | Women | 2 | 0.1048 | 0.0091 | 0.0883 | 0.1240 | 0.0513 | 0.0432 | 0.0607 | 0.3393 | 0.5825 | 3.1368 | 1.7711 |
| Eritrea | Women | 3 | 0.0299 | 0.0045 | 0.0221 | 0.0402 | 0.0146 | 0.0108 | 0.0197 | 0.2743 | 0.5238 | 2.4251 | 1.5573 |
| Eswatini | Men | 0 | 0.0405 | 0.0091 | 0.0259 | 0.0627 | 0.0198 | 0.0127 | 0.0307 | 0.0540 | 0.2325 | 0.9432 | 0.9712 |
| Eswatini | Men | 1 | 0.5748 | 0.0323 | 0.5106 | 0.6366 | 0.2814 | 0.2500 | 0.3117 | 0.1076 | 0.3280 | 2.3390 | 1.5294 |
| Eswatini | Men | 2 | 0.2620 | 0.0251 | 0.2159 | 0.3140 | 0.1283 | 0.1057 | 0.1537 | 0.0817 | 0.2859 | 1.7725 | 1.3314 |
| Eswatini | Men | 3 | 0.1227 | 0.0172 | 0.0928 | 0.1606 | 0.0601 | 0.0454 | 0.0786 | 0.0692 | 0.2630 | 1.3423 | 1.1586 |
| Eswatini | Women | 0 | 0.0173 | 0.0047 | 0.0102 | 0.0293 | 0.0085 | 0.0050 | 0.0143 | 0.0394 | 0.1985 | 1.1956 | 1.0935 |
| Eswatini | Women | 1 | 0.2802 | 0.0182 | 0.2460 | 0.3171 | 0.1372 | 0.1204 | 0.1553 | 0.0502 | 0.2241 | 1.7870 | 1.3368 |
| Eswatini | Women | 2 | 0.2988 | 0.0189 | 0.2631 | 0.3370 | 0.1463 | 0.1288 | 0.1650 | 0.0522 | 0.2284 | 1.8720 | 1.3682 |
| Eswatini | Women | 3 | 0.4037 | 0.0188 | 0.3675 | 0.4410 | 0.1977 | 0.1799 | 0.2159 | 0.0450 | 0.2120 | 1.5835 | 1.2584 |
| Ethiopia | Men | 0 | 0.2305 | 0.0144 | 0.2034 | 0.2600 | 0.1128 | 0.0996 | 0.1273 | 4.8713 | 2.2071 | 2.9494 | 1.7174 |
| Ethiopia | Men | 1 | 0.7166 | 0.0149 | 0.6864 | 0.7450 | 0.3509 | 0.3361 | 0.3647 | 4.5496 | 2.1330 | 2.6956 | 1.6418 |
| Ethiopia | Men | 2 | 0.0465 | 0.0061 | 0.0358 | 0.0601 | 0.0228 | 0.0175 | 0.0294 | 3.5184 | 1.8757 | 1.6508 | 1.2848 |
| Ethiopia | Men | 3 | 0.0064 | 0.0021 | 0.0033 | 0.0124 | 0.0032 | 0.0016 | 0.0061 | 2.9849 | 1.7277 | 1.3937 | 1.1806 |
| Ethiopia | Women | 0 | 0.2133 | 0.0128 | 0.1892 | 0.2396 | 0.1044 | 0.0926 | 0.1173 | 3.3530 | 1.8311 | 3.6095 | 1.8999 |
| Ethiopia | Women | 1 | 0.6820 | 0.0126 | 0.6567 | 0.7063 | 0.3339 | 0.3215 | 0.3458 | 2.5152 | 1.5859 | 2.4906 | 1.5782 |
| Ethiopia | Women | 2 | 0.0778 | 0.0071 | 0.0649 | 0.0930 | 0.0381 | 0.0318 | 0.0456 | 2.4261 | 1.5576 | 1.9222 | 1.3864 |
| Ethiopia | Women | 3 | 0.0268 | 0.0036 | 0.0206 | 0.0349 | 0.0131 | 0.0101 | 0.0171 | 1.7111 | 1.3081 | 1.1439 | 1.0695 |
| French Polynesia | Men | 0 | 0.0041 | 0.0023 | 0.0014 | 0.0123 | 0.0020 | 0.0007 | 0.0060 | 0.0203 | 0.1423 | 0.7248 | 0.8513 |
| French Polynesia | Men | 1 | 0.2000 | 0.0177 | 0.1675 | 0.2369 | 0.0979 | 0.0820 | 0.1160 | 0.0306 | 0.1750 | 1.4412 | 1.2005 |
| French Polynesia | Men | 2 | 0.3486 | 0.0221 | 0.3066 | 0.3931 | 0.1707 | 0.1501 | 0.1925 | 0.0337 | 0.1836 | 1.5825 | 1.2580 |
| French Polynesia | Men | 3 | 0.4473 | 0.0243 | 0.4003 | 0.4952 | 0.2190 | 0.1960 | 0.2425 | 0.0374 | 0.1933 | 1.7568 | 1.3254 |
| French Polynesia | Women | 0 | 0.0165 | 0.0044 | 0.0098 | 0.0278 | 0.0081 | 0.0048 | 0.0136 | 0.0176 | 0.1328 | 0.9616 | 0.9806 |
| French Polynesia | Women | 1 | 0.2319 | 0.0173 | 0.1997 | 0.2675 | 0.1135 | 0.0978 | 0.1310 | 0.0249 | 0.1579 | 1.5311 | 1.2374 |
| French Polynesia | Women | 2 | 0.2698 | 0.0182 | 0.2356 | 0.3069 | 0.1321 | 0.1153 | 0.1503 | 0.0249 | 0.1579 | 1.7217 | 1.3121 |
| French Polynesia | Women | 3 | 0.4818 | 0.0205 | 0.4419 | 0.5220 | 0.2359 | 0.2164 | 0.2556 | 0.0248 | 0.1575 | 1.6601 | 1.2885 |
| Georgia | Men | 0 | 0.0192 | 0.0082 | 0.0083 | 0.0439 | 0.0094 | 0.0041 | 0.0215 | 0.7086 | 0.8418 | 3.3555 | 1.8318 |
| Georgia | Men | 1 | 0.2685 | 0.0210 | 0.2294 | 0.3115 | 0.1315 | 0.1123 | 0.1525 | 0.4461 | 0.6679 | 1.5199 | 1.2328 |
| Georgia | Men | 2 | 0.3557 | 0.0224 | 0.3131 | 0.4008 | 0.1742 | 0.1533 | 0.1962 | 0.4368 | 0.6609 | 1.4590 | 1.2079 |
| Georgia | Men | 3 | 0.3566 | 0.0223 | 0.3142 | 0.4013 | 0.1746 | 0.1538 | 0.1965 | 0.4311 | 0.6566 | 1.4211 | 1.1921 |
| Georgia | Women | 0 | 0.0252 | 0.0044 | 0.0178 | 0.0355 | 0.0123 | 0.0087 | 0.0174 | 0.1782 | 0.4222 | 1.7298 | 1.3152 |
| Georgia | Women | 1 | 0.2901 | 0.0143 | 0.2629 | 0.3189 | 0.1420 | 0.1287 | 0.1561 | 0.2210 | 0.4702 | 1.9497 | 1.3963 |
| Georgia | Women | 2 | 0.2998 | 0.0135 | 0.2739 | 0.3270 | 0.1468 | 0.1341 | 0.1601 | 0.1943 | 0.4408 | 1.5818 | 1.2577 |
| Georgia | Women | 3 | 0.3849 | 0.0143 | 0.3573 | 0.4133 | 0.1885 | 0.1749 | 0.2024 | 0.1921 | 0.4383 | 1.4813 | 1.2171 |
| Iraq | Men | 0 | 0.0195 | 0.0163 | 0.0037 | 0.0969 | 0.0096 | 0.0018 | 0.0474 | 7.0649 | 2.6580 | 5.2479 | 2.2908 |
| Iraq | Men | 1 | 0.3080 | 0.0384 | 0.2375 | 0.3887 | 0.1508 | 0.1163 | 0.1903 | 3.5269 | 1.8780 | 2.1314 | 1.4599 |
| Iraq | Men | 2 | 0.3571 | 0.0383 | 0.2852 | 0.4360 | 0.1748 | 0.1396 | 0.2135 | 3.2665 | 1.8073 | 1.7297 | 1.3152 |
| Iraq | Men | 3 | 0.3154 | 0.0400 | 0.2420 | 0.3995 | 0.1544 | 0.1185 | 0.1956 | 3.7879 | 1.9463 | 1.9942 | 1.4122 |
| Iraq | Women | 0 | 0.0118 | 0.0051 | 0.0050 | 0.0277 | 0.0058 | 0.0024 | 0.0135 | 1.2444 | 1.1156 | 0.9945 | 0.9973 |
| Iraq | Women | 1 | 0.1691 | 0.0230 | 0.1283 | 0.2196 | 0.0828 | 0.0628 | 0.1075 | 2.0874 | 1.4448 | 2.0795 | 1.4421 |
| Iraq | Women | 2 | 0.3278 | 0.0261 | 0.2783 | 0.3814 | 0.1605 | 0.1363 | 0.1867 | 1.7132 | 1.3089 | 1.5836 | 1.2584 |
| Iraq | Women | 3 | 0.4913 | 0.0272 | 0.4378 | 0.5451 | 0.2406 | 0.2143 | 0.2669 | 1.6382 | 1.2799 | 1.5131 | 1.2301 |
| Kenya | Men | 0 | 0.1336 | 0.0200 | 0.0990 | 0.1778 | 0.0654 | 0.0485 | 0.0870 | 8.2265 | 2.8682 | 4.3042 | 2.0747 |
| Kenya | Men | 1 | 0.6589 | 0.0265 | 0.6052 | 0.7089 | 0.3226 | 0.2963 | 0.3471 | 7.4735 | 2.7338 | 3.8291 | 1.9568 |
| Kenya | Men | 2 | 0.1448 | 0.0173 | 0.1141 | 0.1821 | 0.0709 | 0.0559 | 0.0892 | 5.7631 | 2.4007 | 2.7379 | 1.6547 |
| Kenya | Men | 3 | 0.0626 | 0.0134 | 0.0410 | 0.0947 | 0.0307 | 0.0201 | 0.0464 | 7.3188 | 2.7053 | 3.9113 | 1.9777 |
| Kenya | Women | 0 | 0.0916 | 0.0121 | 0.0705 | 0.1182 | 0.0449 | 0.0345 | 0.0579 | 4.2037 | 2.0503 | 3.1835 | 1.7842 |
| Kenya | Women | 1 | 0.4776 | 0.0218 | 0.4352 | 0.5203 | 0.2338 | 0.2131 | 0.2548 | 4.5372 | 2.1301 | 3.4601 | 1.8601 |
| Kenya | Women | 2 | 0.2633 | 0.0176 | 0.2302 | 0.2992 | 0.1289 | 0.1127 | 0.1465 | 3.8252 | 1.9558 | 2.8490 | 1.6879 |
| Kenya | Women | 3 | 0.1675 | 0.0151 | 0.1399 | 0.1993 | 0.0820 | 0.0685 | 0.0976 | 3.9200 | 1.9799 | 3.0520 | 1.7470 |
| Kiribati | Men | 0 | 0.0025 | 0.0019 | 0.0006 | 0.0107 | 0.0012 | 0.0003 | 0.0053 | 0.0062 | 0.0790 | 0.1863 | 0.4316 |
| Kiribati | Men | 1 | 0.2123 | 0.0523 | 0.1275 | 0.3322 | 0.1040 | 0.0624 | 0.1626 | 0.0734 | 0.2709 | 6.1959 | 2.4892 |
| Kiribati | Men | 2 | 0.4411 | 0.0574 | 0.3333 | 0.5547 | 0.2160 | 0.1632 | 0.2716 | 0.0600 | 0.2450 | 5.4392 | 2.3322 |
| Kiribati | Men | 3 | 0.3440 | 0.0553 | 0.2449 | 0.4589 | 0.1684 | 0.1199 | 0.2247 | 0.0609 | 0.2469 | 5.2464 | 2.2905 |
| Kiribati | Women | 0 | 0.0037 | 0.0021 | 0.0012 | 0.0114 | 0.0018 | 0.0006 | 0.0056 | 0.0073 | 0.0855 | 0.1878 | 0.4333 |
| Kiribati | Women | 1 | 0.1364 | 0.0295 | 0.0882 | 0.2051 | 0.0668 | 0.0432 | 0.1004 | 0.0436 | 0.2089 | 3.1357 | 1.7708 |
| Kiribati | Women | 2 | 0.2634 | 0.0308 | 0.2075 | 0.3281 | 0.1289 | 0.1016 | 0.1606 | 0.0290 | 0.1704 | 2.4627 | 1.5693 |
| Kiribati | Women | 3 | 0.5965 | 0.0245 | 0.5476 | 0.6436 | 0.2921 | 0.2681 | 0.3151 | 0.0148 | 0.1217 | 1.2170 | 1.1032 |
| Kuwait | Men | 0 | 0.0017 | 0.0017 | 0.0002 | 0.0121 | 0.0008 | 0.0001 | 0.0059 | 0.1244 | 0.3527 | 0.9403 | 0.9697 |
| Kuwait | Men | 1 | 0.1544 | 0.0155 | 0.1263 | 0.1873 | 0.0756 | 0.0618 | 0.0917 | 0.1337 | 0.3656 | 1.0623 | 1.0307 |
| Kuwait | Men | 2 | 0.4359 | 0.0211 | 0.3950 | 0.4777 | 0.2134 | 0.1934 | 0.2339 | 0.1314 | 0.3624 | 1.0253 | 1.0126 |
| Kuwait | Men | 3 | 0.4080 | 0.0209 | 0.3678 | 0.4495 | 0.1998 | 0.1801 | 0.2201 | 0.1304 | 0.3612 | 1.0113 | 1.0056 |
| Kuwait | Women | 0 | 0.0024 | 0.0017 | 0.0006 | 0.0098 | 0.0012 | 0.0003 | 0.0048 | 0.0982 | 0.3134 | 1.2892 | 1.1354 |
| Kuwait | Women | 1 | 0.1770 | 0.0130 | 0.1529 | 0.2040 | 0.0867 | 0.0748 | 0.0999 | 0.0913 | 0.3022 | 1.1217 | 1.0591 |
| Kuwait | Women | 2 | 0.3287 | 0.0157 | 0.2987 | 0.3602 | 0.1609 | 0.1462 | 0.1763 | 0.0874 | 0.2956 | 1.0338 | 1.0167 |
| Kuwait | Women | 3 | 0.4919 | 0.0167 | 0.4592 | 0.5246 | 0.2408 | 0.2248 | 0.2569 | 0.0873 | 0.2955 | 1.0344 | 1.0170 |
| Kyrgyzstan | Men | 0 | 0.0173 | 0.0061 | 0.0087 | 0.0344 | 0.0085 | 0.0042 | 0.0168 | 2.7500 | 1.6583 | 2.4207 | 1.5558 |
| Kyrgyzstan | Men | 1 | 0.4904 | 0.0368 | 0.4190 | 0.5623 | 0.2401 | 0.2051 | 0.2753 | 6.8336 | 2.6141 | 5.1257 | 2.2640 |
| Kyrgyzstan | Men | 2 | 0.3176 | 0.0296 | 0.2626 | 0.3783 | 0.1555 | 0.1285 | 0.1852 | 5.1064 | 2.2597 | 3.4799 | 1.8654 |
| Kyrgyzstan | Men | 3 | 0.1746 | 0.0161 | 0.1453 | 0.2084 | 0.0855 | 0.0711 | 0.1020 | 2.2596 | 1.5032 | 1.3801 | 1.1748 |
| Kyrgyzstan | Women | 0 | 0.0249 | 0.0063 | 0.0151 | 0.0409 | 0.0122 | 0.0074 | 0.0200 | 1.9530 | 1.3975 | 3.8372 | 1.9589 |
| Kyrgyzstan | Women | 1 | 0.3416 | 0.0162 | 0.3105 | 0.3740 | 0.1672 | 0.1520 | 0.1831 | 1.3770 | 1.1735 | 1.8888 | 1.3743 |
| Kyrgyzstan | Women | 2 | 0.3413 | 0.0138 | 0.3148 | 0.3688 | 0.1671 | 0.1541 | 0.1806 | 0.9961 | 0.9980 | 1.3244 | 1.1508 |
| Kyrgyzstan | Women | 3 | 0.2922 | 0.0143 | 0.2650 | 0.3211 | 0.1431 | 0.1297 | 0.1572 | 1.1684 | 1.0809 | 1.4571 | 1.2071 |
| Lao People's Democratic Republic | Men | 0 | 0.0895 | 0.0123 | 0.0681 | 0.1167 | 0.0438 | 0.0333 | 0.0571 | 0.5650 | 0.7517 | 1.5778 | 1.2561 |
| Lao People's Democratic Republic | Men | 1 | 0.6881 | 0.0199 | 0.6477 | 0.7258 | 0.3369 | 0.3171 | 0.3553 | 0.5633 | 0.7505 | 1.5770 | 1.2558 |
| Lao People's Democratic Republic | Men | 2 | 0.1861 | 0.0164 | 0.1562 | 0.2204 | 0.0911 | 0.0765 | 0.1079 | 0.5374 | 0.7331 | 1.5519 | 1.2458 |
| Lao People's Democratic Republic | Men | 3 | 0.0363 | 0.0076 | 0.0240 | 0.0546 | 0.0178 | 0.0117 | 0.0267 | 0.5059 | 0.7112 | 1.3132 | 1.1459 |
| Lao People's Democratic Republic | Women | 0 | 0.1079 | 0.0138 | 0.0837 | 0.1381 | 0.0528 | 0.0410 | 0.0676 | 0.8398 | 0.9164 | 2.5668 | 1.6021 |
| Lao People's Democratic Republic | Women | 1 | 0.5803 | 0.0191 | 0.5424 | 0.6172 | 0.2841 | 0.2656 | 0.3022 | 0.6361 | 0.7975 | 1.8662 | 1.3661 |
| Lao People's Democratic Republic | Women | 2 | 0.2295 | 0.0170 | 0.1979 | 0.2645 | 0.1124 | 0.0969 | 0.1295 | 0.6929 | 0.8324 | 2.0278 | 1.4240 |
| Lao People's Democratic Republic | Women | 3 | 0.0823 | 0.0107 | 0.0637 | 0.1058 | 0.0403 | 0.0312 | 0.0518 | 0.6389 | 0.7993 | 1.9394 | 1.3926 |
| Lebanon | Men | 0 | 0.0139 | 0.0107 | 0.0031 | 0.0609 | 0.0068 | 0.0015 | 0.0298 | 3.6710 | 1.9160 | 4.4708 | 2.1144 |
| Lebanon | Men | 1 | 0.2651 | 0.0364 | 0.2001 | 0.3421 | 0.1298 | 0.0980 | 0.1675 | 3.0052 | 1.7336 | 2.3418 | 1.5303 |
| Lebanon | Men | 2 | 0.3833 | 0.0428 | 0.3035 | 0.4700 | 0.1877 | 0.1486 | 0.2301 | 3.4385 | 1.8543 | 2.5678 | 1.6024 |
| Lebanon | Men | 3 | 0.3377 | 0.0426 | 0.2598 | 0.4255 | 0.1653 | 0.1272 | 0.2083 | 3.5933 | 1.8956 | 2.8936 | 1.7011 |
| Lebanon | Women | 0 | 0.0129 | 0.0042 | 0.0067 | 0.0244 | 0.0063 | 0.0033 | 0.0119 | 0.7118 | 0.8437 | 0.7279 | 0.8531 |
| Lebanon | Women | 1 | 0.3796 | 0.0356 | 0.3127 | 0.4514 | 0.1859 | 0.1531 | 0.2210 | 2.7326 | 1.6531 | 3.4471 | 1.8566 |
| Lebanon | Women | 2 | 0.3212 | 0.0242 | 0.2757 | 0.3703 | 0.1572 | 0.1350 | 0.1813 | 1.3617 | 1.1669 | 1.5823 | 1.2579 |
| Lebanon | Women | 3 | 0.2864 | 0.0357 | 0.2218 | 0.3611 | 0.1402 | 0.1086 | 0.1768 | 3.1678 | 1.7798 | 3.5137 | 1.8745 |
| Lesotho | Men | 0 | 0.0787 | 0.0143 | 0.0548 | 0.1116 | 0.0385 | 0.0268 | 0.0547 | 0.1616 | 0.4020 | 1.5859 | 1.2593 |
| Lesotho | Men | 1 | 0.6727 | 0.0267 | 0.6183 | 0.7228 | 0.3293 | 0.3027 | 0.3539 | 0.1861 | 0.4314 | 1.9769 | 1.4060 |
| Lesotho | Men | 2 | 0.1687 | 0.0193 | 0.1342 | 0.2099 | 0.0826 | 0.0657 | 0.1028 | 0.1516 | 0.3894 | 1.6067 | 1.2676 |
| Lesotho | Men | 3 | 0.0800 | 0.0157 | 0.0541 | 0.1168 | 0.0392 | 0.0265 | 0.0572 | 0.1931 | 0.4395 | 2.0226 | 1.4222 |
| Lesotho | Women | 0 | 0.0331 | 0.0074 | 0.0212 | 0.0512 | 0.0162 | 0.0104 | 0.0251 | 0.1011 | 0.3179 | 1.9037 | 1.3798 |
| Lesotho | Women | 1 | 0.3679 | 0.0202 | 0.3291 | 0.4084 | 0.1801 | 0.1611 | 0.1999 | 0.1029 | 0.3208 | 2.2126 | 1.4875 |
| Lesotho | Women | 2 | 0.2685 | 0.0173 | 0.2360 | 0.3038 | 0.1315 | 0.1155 | 0.1487 | 0.0890 | 0.2984 | 1.8558 | 1.3623 |
| Lesotho | Women | 3 | 0.3305 | 0.0183 | 0.2956 | 0.3674 | 0.1618 | 0.1447 | 0.1799 | 0.0886 | 0.2976 | 1.9176 | 1.3848 |
| Libya | Men | 0 | 0.0408 | 0.0107 | 0.0242 | 0.0678 | 0.0200 | 0.0119 | 0.0332 | 0.5848 | 0.7647 | 3.7713 | 1.9420 |
| Libya | Men | 1 | 0.3801 | 0.0226 | 0.3368 | 0.4253 | 0.1861 | 0.1649 | 0.2082 | 0.4317 | 0.6571 | 2.1347 | 1.4611 |
| Libya | Men | 2 | 0.3590 | 0.0228 | 0.3156 | 0.4048 | 0.1758 | 0.1545 | 0.1982 | 0.4491 | 0.6701 | 1.9906 | 1.4109 |
| Libya | Men | 3 | 0.2201 | 0.0185 | 0.1860 | 0.2585 | 0.1078 | 0.0911 | 0.1266 | 0.3955 | 0.6289 | 1.8072 | 1.3443 |
| Libya | Women | 0 | 0.0384 | 0.0117 | 0.0211 | 0.0692 | 0.0188 | 0.0103 | 0.0339 | 0.6299 | 0.7937 | 5.3520 | 2.3134 |
| Libya | Women | 1 | 0.2598 | 0.0274 | 0.2098 | 0.3169 | 0.1272 | 0.1027 | 0.1552 | 0.6640 | 0.8149 | 3.1376 | 1.7713 |
| Libya | Women | 2 | 0.2893 | 0.0222 | 0.2479 | 0.3346 | 0.1416 | 0.1214 | 0.1638 | 0.4071 | 0.6380 | 1.6889 | 1.2996 |
| Libya | Women | 3 | 0.4124 | 0.0281 | 0.3586 | 0.4684 | 0.2019 | 0.1756 | 0.2293 | 0.5566 | 0.7461 | 2.3464 | 1.5318 |
| Malawi | Men | 0 | 0.0667 | 0.0100 | 0.0495 | 0.0894 | 0.0327 | 0.0242 | 0.0438 | 1.6080 | 1.2681 | 1.3337 | 1.1548 |
| Malawi | Men | 1 | 0.8170 | 0.0195 | 0.7753 | 0.8524 | 0.4000 | 0.3796 | 0.4173 | 2.5402 | 1.5938 | 2.2139 | 1.4879 |
| Malawi | Men | 2 | 0.1023 | 0.0168 | 0.0736 | 0.1403 | 0.0501 | 0.0361 | 0.0687 | 3.0461 | 1.7453 | 2.8151 | 1.6778 |
| Malawi | Men | 3 | 0.0141 | 0.0036 | 0.0085 | 0.0233 | 0.0069 | 0.0041 | 0.0114 | 0.9412 | 0.9702 | 0.4919 | 0.7013 |
| Malawi | Women | 0 | 0.0543 | 0.0099 | 0.0377 | 0.0774 | 0.0266 | 0.0185 | 0.0379 | 2.0023 | 1.4150 | 3.1821 | 1.7838 |
| Malawi | Women | 1 | 0.6393 | 0.0217 | 0.5956 | 0.6808 | 0.3130 | 0.2916 | 0.3333 | 2.1329 | 1.4604 | 3.4682 | 1.8623 |
| Malawi | Women | 2 | 0.1926 | 0.0162 | 0.1627 | 0.2266 | 0.0943 | 0.0796 | 0.1109 | 1.7707 | 1.3307 | 2.8363 | 1.6841 |
| Malawi | Women | 3 | 0.1139 | 0.0116 | 0.0929 | 0.1389 | 0.0557 | 0.0455 | 0.0680 | 1.4069 | 1.1861 | 2.0968 | 1.4480 |
| Mongolia | Men | 0 | 0.0205 | 0.0036 | 0.0146 | 0.0289 | 0.0101 | 0.0071 | 0.0141 | 0.1557 | 0.3946 | 1.5156 | 1.2311 |
| Mongolia | Men | 1 | 0.3943 | 0.0124 | 0.3702 | 0.4189 | 0.1930 | 0.1812 | 0.2051 | 0.1576 | 0.3970 | 1.4811 | 1.2170 |
| Mongolia | Men | 2 | 0.3795 | 0.0122 | 0.3558 | 0.4037 | 0.1858 | 0.1742 | 0.1977 | 0.1544 | 0.3930 | 1.4445 | 1.2019 |
| Mongolia | Men | 3 | 0.2057 | 0.0099 | 0.1869 | 0.2259 | 0.1007 | 0.0915 | 0.1106 | 0.1469 | 0.3833 | 1.3632 | 1.1676 |
| Mongolia | Women | 0 | 0.0191 | 0.0038 | 0.0130 | 0.0282 | 0.0094 | 0.0064 | 0.0138 | 0.1880 | 0.4336 | 2.7282 | 1.6517 |
| Mongolia | Women | 1 | 0.3685 | 0.0111 | 0.3469 | 0.3906 | 0.1804 | 0.1698 | 0.1912 | 0.1317 | 0.3630 | 1.5225 | 1.2339 |
| Mongolia | Women | 2 | 0.3574 | 0.0103 | 0.3376 | 0.3778 | 0.1750 | 0.1653 | 0.1850 | 0.1130 | 0.3362 | 1.2558 | 1.1206 |
| Mongolia | Women | 3 | 0.2550 | 0.0099 | 0.2360 | 0.2749 | 0.1248 | 0.1156 | 0.1346 | 0.1275 | 0.3571 | 1.3805 | 1.1749 |
| Morocco | Men | 0 | 0.0520 | 0.0081 | 0.0383 | 0.0704 | 0.0255 | 0.0187 | 0.0345 | 0.0009 | 0.0298 | 1.7959 | 1.3401 |
| Morocco | Men | 1 | 0.4698 | 0.0164 | 0.4378 | 0.5020 | 0.2300 | 0.2143 | 0.2458 | 0.0007 | 0.0269 | 1.2389 | 1.1130 |
| Morocco | Men | 2 | 0.3474 | 0.0155 | 0.3177 | 0.3783 | 0.1701 | 0.1556 | 0.1852 | 0.0007 | 0.0266 | 1.1859 | 1.0890 |
| Morocco | Men | 3 | 0.1308 | 0.0114 | 0.1100 | 0.1547 | 0.0640 | 0.0539 | 0.0758 | 0.0008 | 0.0276 | 1.3253 | 1.1512 |
| Morocco | Women | 0 | 0.0206 | 0.0034 | 0.0150 | 0.0283 | 0.0101 | 0.0073 | 0.0139 | 0.0004 | 0.0194 | 1.4557 | 1.2065 |
| Morocco | Women | 1 | 0.2688 | 0.0099 | 0.2498 | 0.2887 | 0.1316 | 0.1223 | 0.1413 | 0.0003 | 0.0184 | 1.1757 | 1.0843 |
| Morocco | Women | 2 | 0.3747 | 0.0107 | 0.3540 | 0.3958 | 0.1834 | 0.1733 | 0.1938 | 0.0003 | 0.0182 | 1.1206 | 1.0586 |
| Morocco | Women | 3 | 0.3359 | 0.0104 | 0.3159 | 0.3565 | 0.1645 | 0.1547 | 0.1745 | 0.0003 | 0.0181 | 1.0963 | 1.0470 |
| Myanmar | Men | 0 | 0.1879 | 0.0223 | 0.1480 | 0.2356 | 0.0920 | 0.0725 | 0.1153 | 31.7364 | 5.6335 | 8.0033 | 2.8290 |
| Myanmar | Men | 1 | 0.6430 | 0.0173 | 0.6084 | 0.6761 | 0.3148 | 0.2979 | 0.3310 | 12.6649 | 3.5588 | 3.3496 | 1.8302 |
| Myanmar | Men | 2 | 0.1343 | 0.0124 | 0.1117 | 0.1606 | 0.0657 | 0.0547 | 0.0786 | 12.9649 | 3.6007 | 3.4002 | 1.8440 |
| Myanmar | Men | 3 | 0.0348 | 0.0083 | 0.0217 | 0.0554 | 0.0170 | 0.0106 | 0.0271 | 20.1935 | 4.4937 | 6.3580 | 2.5215 |
| Myanmar | Women | 0 | 0.1300 | 0.0192 | 0.0968 | 0.1725 | 0.0637 | 0.0474 | 0.0845 | 31.3978 | 5.6034 | 14.5700 | 3.8171 |
| Myanmar | Women | 1 | 0.5213 | 0.0163 | 0.4893 | 0.5531 | 0.2552 | 0.2396 | 0.2708 | 10.2643 | 3.2038 | 5.3266 | 2.3079 |
| Myanmar | Women | 2 | 0.2665 | 0.0283 | 0.2149 | 0.3254 | 0.1305 | 0.1052 | 0.1593 | 39.3807 | 6.2754 | 21.5189 | 4.6389 |
| Myanmar | Women | 3 | 0.0822 | 0.0070 | 0.0695 | 0.0970 | 0.0402 | 0.0340 | 0.0475 | 6.2589 | 2.5018 | 3.1505 | 1.7750 |
| Nauru | Men | 0 | 0.0000 | 0.0000 | 0.0000 | 0.0000 | 0.0000 | 0.0000 | 0.0000 | 0.0000 | 0.0000 | 0.0000 | 0.0000 |
| Nauru | Men | 1 | 0.0610 | 0.0172 | 0.0348 | 0.1048 | 0.0299 | 0.0170 | 0.0513 | 0.0023 | 0.0476 | 1.7803 | 1.3343 |
| Nauru | Men | 2 | 0.1741 | 0.0174 | 0.1426 | 0.2108 | 0.0852 | 0.0698 | 0.1032 | 0.0009 | 0.0303 | 0.7219 | 0.8496 |
| Nauru | Men | 3 | 0.7649 | 0.0171 | 0.7296 | 0.7968 | 0.3745 | 0.3572 | 0.3901 | 0.0007 | 0.0268 | 0.5622 | 0.7498 |
| Nauru | Women | 0 | 0.0000 | 0.0000 | 0.0000 | 0.0000 | 0.0000 | 0.0000 | 0.0000 | 0.0000 | 0.0000 | 0.0000 | 0.0000 |
| Nauru | Women | 1 | 0.0754 | 0.0127 | 0.0539 | 0.1045 | 0.0369 | 0.0264 | 0.0512 | 0.0011 | 0.0326 | 0.9404 | 0.9697 |
| Nauru | Women | 2 | 0.1701 | 0.0168 | 0.1396 | 0.2056 | 0.0833 | 0.0684 | 0.1007 | 0.0009 | 0.0302 | 0.8083 | 0.8990 |
| Nauru | Women | 3 | 0.7545 | 0.0160 | 0.7218 | 0.7844 | 0.3694 | 0.3534 | 0.3841 | 0.0006 | 0.0250 | 0.5541 | 0.7444 |
| Nepal | Men | 0 | 0.0829 | 0.0151 | 0.0578 | 0.1176 | 0.0406 | 0.0283 | 0.0576 | 6.2641 | 2.5028 | 5.0626 | 2.2500 |
| Nepal | Men | 1 | 0.6291 | 0.0220 | 0.5851 | 0.6710 | 0.3080 | 0.2865 | 0.3285 | 4.3379 | 2.0828 | 2.9899 | 1.7291 |
| Nepal | Men | 2 | 0.2421 | 0.0193 | 0.2063 | 0.2819 | 0.1185 | 0.1010 | 0.1380 | 4.2564 | 2.0631 | 2.8873 | 1.6992 |
| Nepal | Men | 3 | 0.0459 | 0.0081 | 0.0324 | 0.0648 | 0.0225 | 0.0159 | 0.0317 | 3.1710 | 1.7807 | 1.9583 | 1.3994 |
| Nepal | Women | 0 | 0.0868 | 0.0094 | 0.0700 | 0.1071 | 0.0425 | 0.0343 | 0.0524 | 2.6883 | 1.6396 | 3.1705 | 1.7806 |
| Nepal | Women | 1 | 0.6133 | 0.0186 | 0.5764 | 0.6490 | 0.3003 | 0.2822 | 0.3178 | 3.4962 | 1.8698 | 3.8171 | 1.9538 |
| Nepal | Women | 2 | 0.2323 | 0.0146 | 0.2049 | 0.2622 | 0.1137 | 0.1003 | 0.1284 | 2.8861 | 1.6989 | 3.1457 | 1.7736 |
| Nepal | Women | 3 | 0.0675 | 0.0077 | 0.0539 | 0.0843 | 0.0331 | 0.0264 | 0.0413 | 2.2734 | 1.5078 | 2.2268 | 1.4922 |
| Niue | Men | 0 | 0.0000 | 0.0000 | 0.0000 | 0.0000 | 0.0000 | 0.0000 | 0.0000 | 0.0000 | 0.0000 | 0.0000 | 0.0000 |
| Niue | Men | 1 | 0.0733 | 0.0171 | 0.0460 | 0.1148 | 0.0359 | 0.0225 | 0.0562 | 0.0005 | 0.0225 | 0.9907 | 0.9953 |
| Niue | Men | 2 | 0.2537 | 0.0288 | 0.2015 | 0.3141 | 0.1242 | 0.0987 | 0.1538 | 0.0005 | 0.0227 | 1.0131 | 1.0065 |
| Niue | Men | 3 | 0.6730 | 0.0310 | 0.6097 | 0.7306 | 0.3295 | 0.2985 | 0.3577 | 0.0005 | 0.0226 | 1.0064 | 1.0032 |
| Niue | Women | 0 | 0.0037 | 0.0037 | 0.0005 | 0.0256 | 0.0018 | 0.0003 | 0.0125 | 0.0005 | 0.0214 | 1.0452 | 1.0223 |
| Niue | Women | 1 | 0.1072 | 0.0185 | 0.0759 | 0.1493 | 0.0525 | 0.0372 | 0.0731 | 0.0004 | 0.0211 | 0.9935 | 0.9968 |
| Niue | Women | 2 | 0.2123 | 0.0245 | 0.1682 | 0.2644 | 0.1040 | 0.0823 | 0.1294 | 0.0004 | 0.0212 | 1.0045 | 1.0022 |
| Niue | Women | 3 | 0.6768 | 0.0280 | 0.6196 | 0.7292 | 0.3314 | 0.3033 | 0.3570 | 0.0004 | 0.0211 | 1.0007 | 1.0003 |
| Qatar | Men | 0 | 0.0086 | 0.0051 | 0.0027 | 0.0270 | 0.0042 | 0.0013 | 0.0132 | 0.0202 | 0.1420 | 1.7098 | 1.3076 |
| Qatar | Men | 1 | 0.2265 | 0.0240 | 0.1828 | 0.2770 | 0.1109 | 0.0895 | 0.1356 | 0.0223 | 0.1492 | 1.5885 | 1.2603 |
| Qatar | Men | 2 | 0.3373 | 0.0227 | 0.2944 | 0.3831 | 0.1652 | 0.1442 | 0.1876 | 0.0155 | 0.1246 | 0.9719 | 0.9858 |
| Qatar | Men | 3 | 0.4275 | 0.0283 | 0.3732 | 0.4837 | 0.2093 | 0.1827 | 0.2368 | 0.0221 | 0.1487 | 1.4779 | 1.2157 |
| Qatar | Women | 0 | 0.0236 | 0.0082 | 0.0119 | 0.0463 | 0.0116 | 0.0058 | 0.0226 | 0.0185 | 0.1359 | 3.1852 | 1.7847 |
| Qatar | Women | 1 | 0.1953 | 0.0176 | 0.1632 | 0.2320 | 0.0956 | 0.0799 | 0.1136 | 0.0125 | 0.1116 | 1.4590 | 1.2079 |
| Qatar | Women | 2 | 0.2359 | 0.0192 | 0.2004 | 0.2755 | 0.1155 | 0.0981 | 0.1349 | 0.0129 | 0.1138 | 1.3580 | 1.1653 |
| Qatar | Women | 3 | 0.5452 | 0.0213 | 0.5033 | 0.5865 | 0.2669 | 0.2464 | 0.2872 | 0.0116 | 0.1077 | 1.2593 | 1.1222 |
| Republic of Moldova | Men | 0 | 0.0105 | 0.0040 | 0.0050 | 0.0222 | 0.0052 | 0.0024 | 0.0108 | 0.1989 | 0.4460 | 1.8161 | 1.3476 |
| Republic of Moldova | Men | 1 | 0.3664 | 0.0212 | 0.3260 | 0.4088 | 0.1794 | 0.1596 | 0.2002 | 0.2486 | 0.4986 | 1.8633 | 1.3650 |
| Republic of Moldova | Men | 2 | 0.4045 | 0.0191 | 0.3677 | 0.4424 | 0.1980 | 0.1800 | 0.2166 | 0.1948 | 0.4413 | 1.4584 | 1.2077 |
| Republic of Moldova | Men | 3 | 0.2186 | 0.0172 | 0.1868 | 0.2541 | 0.1070 | 0.0914 | 0.1244 | 0.2223 | 0.4715 | 1.4530 | 1.2054 |
| Republic of Moldova | Women | 0 | 0.0233 | 0.0060 | 0.0140 | 0.0386 | 0.0114 | 0.0069 | 0.0189 | 0.2069 | 0.4548 | 4.2512 | 2.0618 |
| Republic of Moldova | Women | 1 | 0.3516 | 0.0158 | 0.3213 | 0.3831 | 0.1721 | 0.1573 | 0.1876 | 0.1419 | 0.3767 | 1.9505 | 1.3966 |
| Republic of Moldova | Women | 2 | 0.3016 | 0.0134 | 0.2761 | 0.3284 | 0.1477 | 0.1352 | 0.1608 | 0.1099 | 0.3315 | 1.3984 | 1.1826 |
| Republic of Moldova | Women | 3 | 0.3234 | 0.0146 | 0.2954 | 0.3528 | 0.1584 | 0.1446 | 0.1727 | 0.1271 | 0.3566 | 1.5231 | 1.2342 |
| Rwanda | Men | 0 | 0.0795 | 0.0072 | 0.0666 | 0.0947 | 0.0389 | 0.0326 | 0.0464 | 0.3249 | 0.5700 | 1.3071 | 1.1433 |
| Rwanda | Men | 1 | 0.8183 | 0.0095 | 0.7989 | 0.8362 | 0.4006 | 0.3911 | 0.4094 | 0.2829 | 0.5319 | 1.1567 | 1.0755 |
| Rwanda | Men | 2 | 0.0920 | 0.0074 | 0.0785 | 0.1076 | 0.0451 | 0.0385 | 0.0527 | 0.3036 | 0.5510 | 1.2573 | 1.1213 |
| Rwanda | Men | 3 | 0.0101 | 0.0026 | 0.0061 | 0.0167 | 0.0050 | 0.0030 | 0.0082 | 0.3114 | 0.5580 | 1.2482 | 1.1172 |
| Rwanda | Women | 0 | 0.0650 | 0.0051 | 0.0556 | 0.0759 | 0.0318 | 0.0272 | 0.0371 | 0.2220 | 0.4712 | 1.2971 | 1.1389 |
| Rwanda | Women | 1 | 0.7017 | 0.0106 | 0.6806 | 0.7220 | 0.3436 | 0.3332 | 0.3535 | 0.2715 | 0.5210 | 1.6798 | 1.2961 |
| Rwanda | Women | 2 | 0.1792 | 0.0078 | 0.1643 | 0.1950 | 0.0877 | 0.0805 | 0.0955 | 0.2124 | 0.4609 | 1.3353 | 1.1555 |
| Rwanda | Women | 3 | 0.0541 | 0.0062 | 0.0431 | 0.0677 | 0.0265 | 0.0211 | 0.0332 | 0.3865 | 0.6217 | 2.4303 | 1.5589 |
| Samoa | Men | 0 | 0.0019 | 0.0012 | 0.0005 | 0.0068 | 0.0009 | 0.0002 | 0.0033 | 0.0065 | 0.0808 | 0.1516 | 0.3894 |
| Samoa | Men | 1 | 0.1472 | 0.0138 | 0.1221 | 0.1764 | 0.0721 | 0.0598 | 0.0864 | 0.0121 | 0.1099 | 0.6992 | 0.8362 |
| Samoa | Men | 2 | 0.3171 | 0.0153 | 0.2880 | 0.3478 | 0.1553 | 0.1410 | 0.1703 | 0.0086 | 0.0925 | 0.4757 | 0.6897 |
| Samoa | Men | 3 | 0.5338 | 0.0067 | 0.5206 | 0.5470 | 0.2613 | 0.2549 | 0.2678 | 0.0015 | 0.0381 | 0.0811 | 0.2847 |
| Samoa | Women | 0 | 0.0056 | 0.0032 | 0.0018 | 0.0173 | 0.0028 | 0.0009 | 0.0085 | 0.0144 | 0.1199 | 2.4985 | 1.5807 |
| Samoa | Women | 1 | 0.0697 | 0.0092 | 0.0537 | 0.0901 | 0.0341 | 0.0263 | 0.0441 | 0.0100 | 0.0999 | 0.8990 | 0.9481 |
| Samoa | Women | 2 | 0.1891 | 0.0195 | 0.1537 | 0.2304 | 0.0926 | 0.0752 | 0.1128 | 0.0190 | 0.1379 | 1.7436 | 1.3205 |
| Samoa | Women | 3 | 0.7356 | 0.0216 | 0.6912 | 0.7757 | 0.3601 | 0.3384 | 0.3798 | 0.0183 | 0.1351 | 1.6745 | 1.2940 |
| Sao Tome and Principe | Men | 0 | 0.0642 | 0.0114 | 0.0451 | 0.0907 | 0.0315 | 0.0221 | 0.0444 | 0.0315 | 0.1774 | 1.5860 | 1.2594 |
| Sao Tome and Principe | Men | 1 | 0.6142 | 0.0222 | 0.5699 | 0.6566 | 0.3007 | 0.2790 | 0.3215 | 0.0299 | 0.1729 | 1.1079 | 1.0526 |
| Sao Tome and Principe | Men | 2 | 0.2412 | 0.0185 | 0.2068 | 0.2793 | 0.1181 | 0.1012 | 0.1368 | 0.0270 | 0.1645 | 0.9555 | 0.9775 |
| Sao Tome and Principe | Men | 3 | 0.0804 | 0.0141 | 0.0567 | 0.1128 | 0.0394 | 0.0278 | 0.0552 | 0.0390 | 0.1976 | 1.2515 | 1.1187 |
| Sao Tome and Principe | Women | 0 | 0.0544 | 0.0102 | 0.0375 | 0.0782 | 0.0266 | 0.0184 | 0.0383 | 0.0698 | 0.2642 | 1.8166 | 1.3478 |
| Sao Tome and Principe | Women | 1 | 0.3779 | 0.0209 | 0.3380 | 0.4196 | 0.1850 | 0.1655 | 0.2054 | 0.0638 | 0.2526 | 1.5412 | 1.2415 |
| Sao Tome and Principe | Women | 2 | 0.3290 | 0.0160 | 0.2985 | 0.3611 | 0.1611 | 0.1461 | 0.1768 | 0.0399 | 0.1997 | 0.9915 | 0.9957 |
| Sao Tome and Principe | Women | 3 | 0.2387 | 0.0175 | 0.2061 | 0.2745 | 0.1168 | 0.1009 | 0.1344 | 0.0578 | 0.2405 | 1.4119 | 1.1883 |
| Seychelles | Men | 0 | 0.0460 | 0.0095 | 0.0305 | 0.0688 | 0.0225 | 0.0149 | 0.0337 | 0.0333 | 0.1824 | 1.2124 | 1.1011 |
| Seychelles | Men | 1 | 0.4321 | 0.0221 | 0.3895 | 0.4758 | 0.2116 | 0.1907 | 0.2330 | 0.0318 | 0.1784 | 1.1039 | 1.0507 |
| Seychelles | Men | 2 | 0.3722 | 0.0214 | 0.3313 | 0.4151 | 0.1822 | 0.1622 | 0.2032 | 0.0314 | 0.1772 | 1.0784 | 1.0385 |
| Seychelles | Men | 3 | 0.1497 | 0.0154 | 0.1219 | 0.1824 | 0.0733 | 0.0597 | 0.0893 | 0.0298 | 0.1726 | 0.9766 | 0.9882 |
| Seychelles | Women | 0 | 0.0312 | 0.0074 | 0.0195 | 0.0495 | 0.0153 | 0.0096 | 0.0242 | 0.0294 | 0.1716 | 1.4416 | 1.2007 |
| Seychelles | Women | 1 | 0.2858 | 0.0185 | 0.2509 | 0.3235 | 0.1399 | 0.1228 | 0.1584 | 0.0272 | 0.1650 | 1.2106 | 1.1003 |
| Seychelles | Women | 2 | 0.3319 | 0.0188 | 0.2961 | 0.3697 | 0.1625 | 0.1450 | 0.1810 | 0.0258 | 0.1605 | 1.0719 | 1.0354 |
| Seychelles | Women | 3 | 0.3511 | 0.0188 | 0.3152 | 0.3887 | 0.1719 | 0.1543 | 0.1903 | 0.0250 | 0.1583 | 1.0170 | 1.0085 |
| Solomon Islands | Men | 0 | 0.0015 | 0.0015 | 0.0002 | 0.0107 | 0.0007 | 0.0001 | 0.0052 | 0.0489 | 0.2211 | 0.4003 | 0.6327 |
| Solomon Islands | Men | 1 | 0.3735 | 0.0315 | 0.3142 | 0.4370 | 0.1829 | 0.1538 | 0.2139 | 0.1365 | 0.3694 | 2.4839 | 1.5761 |
| Solomon Islands | Men | 2 | 0.4144 | 0.0305 | 0.3561 | 0.4752 | 0.2029 | 0.1743 | 0.2327 | 0.1239 | 0.3520 | 2.3962 | 1.5480 |
| Solomon Islands | Men | 3 | 0.2106 | 0.0206 | 0.1730 | 0.2538 | 0.1031 | 0.0847 | 0.1243 | 0.0825 | 0.2873 | 1.3203 | 1.1490 |
| Solomon Islands | Women | 0 | 0.0265 | 0.0069 | 0.0159 | 0.0440 | 0.0130 | 0.0078 | 0.0216 | 0.0658 | 0.2565 | 1.3027 | 1.1413 |
| Solomon Islands | Women | 1 | 0.3188 | 0.0210 | 0.2792 | 0.3613 | 0.1561 | 0.1367 | 0.1769 | 0.0721 | 0.2686 | 1.4736 | 1.2139 |
| Solomon Islands | Women | 2 | 0.3426 | 0.0241 | 0.2971 | 0.3912 | 0.1677 | 0.1454 | 0.1915 | 0.0916 | 0.3026 | 1.9067 | 1.3808 |
| Solomon Islands | Women | 3 | 0.3120 | 0.0241 | 0.2668 | 0.3612 | 0.1528 | 0.1306 | 0.1768 | 0.0965 | 0.3106 | 1.9390 | 1.3925 |
| Sri Lanka | Men | 0 | 0.1460 | 0.0122 | 0.1237 | 0.1716 | 0.0715 | 0.0606 | 0.0840 | 2.0104 | 1.4179 | 1.5321 | 1.2378 |
| Sri Lanka | Men | 1 | 0.5836 | 0.0172 | 0.5495 | 0.6169 | 0.2857 | 0.2690 | 0.3020 | 2.0541 | 1.4332 | 1.5895 | 1.2608 |
| Sri Lanka | Men | 2 | 0.2330 | 0.0151 | 0.2046 | 0.2640 | 0.1141 | 0.1002 | 0.1292 | 2.1624 | 1.4705 | 1.7191 | 1.3111 |
| Sri Lanka | Men | 3 | 0.0374 | 0.0055 | 0.0280 | 0.0498 | 0.0183 | 0.0137 | 0.0244 | 1.4258 | 1.1941 | 0.9237 | 0.9611 |
| Sri Lanka | Women | 0 | 0.1276 | 0.0101 | 0.1092 | 0.1487 | 0.0625 | 0.0535 | 0.0728 | 1.5175 | 1.2319 | 2.1167 | 1.4549 |
| Sri Lanka | Women | 1 | 0.4861 | 0.0133 | 0.4602 | 0.5122 | 0.2380 | 0.2253 | 0.2508 | 1.1780 | 1.0854 | 1.4757 | 1.2148 |
| Sri Lanka | Women | 2 | 0.2912 | 0.0117 | 0.2688 | 0.3146 | 0.1426 | 0.1316 | 0.1541 | 1.1080 | 1.0526 | 1.3634 | 1.1676 |
| Sri Lanka | Women | 3 | 0.0951 | 0.0078 | 0.0808 | 0.1116 | 0.0465 | 0.0395 | 0.0546 | 1.1927 | 1.0921 | 1.4746 | 1.2143 |
| Sudan | Men | 0 | 0.1460 | 0.0111 | 0.1256 | 0.1691 | 0.0715 | 0.0615 | 0.0828 | 2.6227 | 1.6195 | 1.7346 | 1.3170 |
| Sudan | Men | 1 | 0.5887 | 0.0154 | 0.5582 | 0.6185 | 0.2882 | 0.2733 | 0.3028 | 2.6204 | 1.6188 | 1.7366 | 1.3178 |
| Sudan | Men | 2 | 0.1899 | 0.0118 | 0.1677 | 0.2142 | 0.0930 | 0.0821 | 0.1049 | 2.4341 | 1.5602 | 1.5379 | 1.2401 |
| Sudan | Men | 3 | 0.0754 | 0.0085 | 0.0604 | 0.0938 | 0.0369 | 0.0296 | 0.0459 | 2.7549 | 1.6598 | 1.7533 | 1.3241 |
| Sudan | Women | 0 | 0.1372 | 0.0106 | 0.1177 | 0.1594 | 0.0672 | 0.0576 | 0.0780 | 2.1532 | 1.4674 | 3.2630 | 1.8064 |
| Sudan | Women | 1 | 0.4410 | 0.0142 | 0.4134 | 0.4690 | 0.2159 | 0.2024 | 0.2296 | 1.8529 | 1.3612 | 2.5997 | 1.6124 |
| Sudan | Women | 2 | 0.2412 | 0.0110 | 0.2202 | 0.2635 | 0.1181 | 0.1078 | 0.1290 | 1.5075 | 1.2278 | 2.0430 | 1.4293 |
| Sudan | Women | 3 | 0.1806 | 0.0127 | 0.1571 | 0.2068 | 0.0884 | 0.0769 | 0.1012 | 2.4527 | 1.5661 | 3.4950 | 1.8695 |
| Tajikistan | Men | 0 | 0.0244 | 0.0090 | 0.0118 | 0.0499 | 0.0119 | 0.0058 | 0.0244 | 2.5282 | 1.5900 | 3.2034 | 1.7898 |
| Tajikistan | Men | 1 | 0.4111 | 0.0252 | 0.3628 | 0.4612 | 0.2013 | 0.1776 | 0.2258 | 1.9442 | 1.3944 | 2.2448 | 1.4983 |
| Tajikistan | Men | 2 | 0.4312 | 0.0276 | 0.3780 | 0.4860 | 0.2111 | 0.1851 | 0.2379 | 2.3108 | 1.5201 | 2.7175 | 1.6485 |
| Tajikistan | Men | 3 | 0.1333 | 0.0188 | 0.1006 | 0.1747 | 0.0653 | 0.0492 | 0.0855 | 2.2712 | 1.5071 | 2.2164 | 1.4887 |
| Tajikistan | Women | 0 | 0.0323 | 0.0082 | 0.0195 | 0.0530 | 0.0158 | 0.0096 | 0.0259 | 1.2173 | 1.1033 | 2.7230 | 1.6502 |
| Tajikistan | Women | 1 | 0.4573 | 0.0245 | 0.4099 | 0.5055 | 0.2239 | 0.2007 | 0.2475 | 1.3530 | 1.1632 | 3.0581 | 1.7488 |
| Tajikistan | Women | 2 | 0.3129 | 0.0207 | 0.2739 | 0.3549 | 0.1532 | 0.1341 | 0.1737 | 1.1183 | 1.0575 | 2.3898 | 1.5459 |
| Tajikistan | Women | 3 | 0.1974 | 0.0162 | 0.1676 | 0.2311 | 0.0967 | 0.0821 | 0.1132 | 0.9286 | 0.9636 | 1.5610 | 1.2494 |
| Timor Leste | Men | 0 | 0.1406 | 0.0218 | 0.1031 | 0.1889 | 0.0688 | 0.0505 | 0.0925 | 0.4319 | 0.6572 | 2.4131 | 1.5534 |
| Timor Leste | Men | 1 | 0.8057 | 0.0484 | 0.6935 | 0.8837 | 0.3945 | 0.3395 | 0.4327 | 1.6490 | 1.2841 | 8.9545 | 2.9924 |
| Timor Leste | Men | 2 | 0.0440 | 0.0237 | 0.0151 | 0.1219 | 0.0216 | 0.0074 | 0.0597 | 1.4698 | 1.2123 | 6.1394 | 2.4778 |
| Timor Leste | Men | 3 | 0.0097 | 0.0055 | 0.0032 | 0.0291 | 0.0047 | 0.0016 | 0.0143 | 0.3456 | 0.5878 | 1.1806 | 1.0866 |
| Timor Leste | Women | 0 | 0.2285 | 0.0306 | 0.1742 | 0.2938 | 0.1119 | 0.0853 | 0.1439 | 0.4569 | 0.6759 | 6.3053 | 2.5110 |
| Timor Leste | Women | 1 | 0.5514 | 0.0627 | 0.4278 | 0.6688 | 0.2699 | 0.2095 | 0.3275 | 1.3706 | 1.1707 | 17.8561 | 4.2257 |
| Timor Leste | Women | 2 | 0.2007 | 0.0413 | 0.1317 | 0.2938 | 0.0983 | 0.0645 | 0.1438 | 0.9165 | 0.9574 | 15.6504 | 3.9561 |
| Timor Leste | Women | 3 | 0.0194 | 0.0090 | 0.0077 | 0.0478 | 0.0095 | 0.0038 | 0.0234 | 0.3715 | 0.6095 | 2.8036 | 1.6744 |
| Togo | Men | 0 | 0.0855 | 0.0163 | 0.0584 | 0.1234 | 0.0419 | 0.0286 | 0.0604 | 0.2050 | 0.4528 | 1.6020 | 1.2657 |
| Togo | Men | 1 | 0.6725 | 0.0292 | 0.6130 | 0.7270 | 0.3293 | 0.3001 | 0.3559 | 0.2321 | 0.4818 | 2.0987 | 1.4487 |
| Togo | Men | 2 | 0.1972 | 0.0218 | 0.1579 | 0.2436 | 0.0966 | 0.0773 | 0.1192 | 0.1808 | 0.4252 | 1.7626 | 1.3277 |
| Togo | Men | 3 | 0.0447 | 0.0141 | 0.0239 | 0.0822 | 0.0219 | 0.0117 | 0.0402 | 0.2805 | 0.5296 | 3.6032 | 1.8982 |
| Togo | Women | 0 | 0.0543 | 0.0103 | 0.0374 | 0.0783 | 0.0266 | 0.0183 | 0.0383 | 0.1606 | 0.4008 | 1.0121 | 1.0061 |
| Togo | Women | 1 | 0.5112 | 0.0265 | 0.4593 | 0.5627 | 0.2503 | 0.2249 | 0.2755 | 0.2198 | 0.4688 | 1.7467 | 1.3216 |
| Togo | Women | 2 | 0.2557 | 0.0238 | 0.2118 | 0.3051 | 0.1252 | 0.1037 | 0.1494 | 0.2340 | 0.4837 | 1.9673 | 1.4026 |
| Togo | Women | 3 | 0.1788 | 0.0237 | 0.1370 | 0.2301 | 0.0876 | 0.0671 | 0.1126 | 0.3001 | 0.5478 | 2.9167 | 1.7078 |
| Tokelau | Men | 0 | 0.0076 | 0.0067 | 0.0014 | 0.0416 | 0.0037 | 0.0007 | 0.0203 | 0.0004 | 0.0197 | 1.7506 | 1.3231 |
| Tokelau | Men | 1 | 0.0657 | 0.0308 | 0.0256 | 0.1584 | 0.0322 | 0.0125 | 0.0775 | 0.0010 | 0.0318 | 3.5646 | 1.8880 |
| Tokelau | Men | 2 | 0.2331 | 0.0298 | 0.1799 | 0.2965 | 0.1141 | 0.0881 | 0.1451 | 0.0003 | 0.0180 | 0.9946 | 0.9973 |
| Tokelau | Men | 3 | 0.6935 | 0.0639 | 0.5565 | 0.8032 | 0.3396 | 0.2725 | 0.3933 | 0.0013 | 0.0355 | 3.9281 | 1.9819 |
| Tokelau | Women | 0 | 0.0000 | 0.0000 | 0.0000 | 0.0000 | 0.0000 | 0.0000 | 0.0000 | 0.0000 | 0.0000 | 0.0000 | 0.0000 |
| Tokelau | Women | 1 | 0.0541 | 0.0114 | 0.0357 | 0.0813 | 0.0265 | 0.0175 | 0.0398 | 0.0002 | 0.0136 | 0.5370 | 0.7328 |
| Tokelau | Women | 2 | 0.1874 | 0.0250 | 0.1431 | 0.2414 | 0.0917 | 0.0701 | 0.1182 | 0.0003 | 0.0173 | 0.9007 | 0.9491 |
| Tokelau | Women | 3 | 0.7585 | 0.0338 | 0.6864 | 0.8184 | 0.3714 | 0.3360 | 0.4007 | 0.0005 | 0.0213 | 1.3511 | 1.1624 |
| Turkmenistan | Men | 0 | 0.0158 | 0.0039 | 0.0098 | 0.0255 | 0.0077 | 0.0048 | 0.0125 | 0.4759 | 0.6899 | 1.1989 | 1.0949 |
| Turkmenistan | Men | 1 | 0.4150 | 0.0192 | 0.3780 | 0.4530 | 0.2032 | 0.1851 | 0.2218 | 0.7474 | 0.8645 | 2.1055 | 1.4510 |
| Turkmenistan | Men | 2 | 0.4134 | 0.0184 | 0.3778 | 0.4499 | 0.2024 | 0.1849 | 0.2203 | 0.6926 | 0.8322 | 1.9088 | 1.3816 |
| Turkmenistan | Men | 3 | 0.1558 | 0.0128 | 0.1322 | 0.1826 | 0.0763 | 0.0647 | 0.0894 | 0.6194 | 0.7870 | 1.6893 | 1.2997 |
| Turkmenistan | Women | 0 | 0.0354 | 0.0056 | 0.0260 | 0.0482 | 0.0174 | 0.0127 | 0.0236 | 0.4162 | 0.6451 | 2.0625 | 1.4362 |
| Turkmenistan | Women | 1 | 0.4564 | 0.0158 | 0.4257 | 0.4874 | 0.2234 | 0.2084 | 0.2386 | 0.4579 | 0.6767 | 1.8219 | 1.3498 |
| Turkmenistan | Women | 2 | 0.3027 | 0.0124 | 0.2790 | 0.3275 | 0.1482 | 0.1366 | 0.1604 | 0.3326 | 0.5767 | 1.2710 | 1.1274 |
| Turkmenistan | Women | 3 | 0.2055 | 0.0126 | 0.1818 | 0.2313 | 0.1006 | 0.0890 | 0.1132 | 0.4460 | 0.6678 | 1.6210 | 1.2732 |
| Tuvalu | Men | 0 | 0.0031 | 0.0029 | 0.0005 | 0.0188 | 0.0015 | 0.0003 | 0.0092 | 0.0018 | 0.0422 | 1.0874 | 1.0428 |
| Tuvalu | Men | 1 | 0.1159 | 0.0143 | 0.0908 | 0.1469 | 0.0568 | 0.0444 | 0.0719 | 0.0013 | 0.0365 | 0.7814 | 0.8840 |
| Tuvalu | Men | 2 | 0.2717 | 0.0235 | 0.2282 | 0.3200 | 0.1330 | 0.1117 | 0.1567 | 0.0019 | 0.0432 | 1.0130 | 1.0065 |
| Tuvalu | Men | 3 | 0.6092 | 0.0306 | 0.5479 | 0.6673 | 0.2983 | 0.2683 | 0.3267 | 0.0026 | 0.0514 | 1.4396 | 1.1998 |
| Tuvalu | Women | 0 | 0.0024 | 0.0010 | 0.0011 | 0.0052 | 0.0012 | 0.0005 | 0.0026 | 0.0002 | 0.0148 | 0.0866 | 0.2943 |
| Tuvalu | Women | 1 | 0.0355 | 0.0074 | 0.0235 | 0.0532 | 0.0174 | 0.0115 | 0.0261 | 0.0009 | 0.0302 | 0.5396 | 0.7346 |
| Tuvalu | Women | 2 | 0.1719 | 0.0184 | 0.1388 | 0.2109 | 0.0842 | 0.0680 | 0.1033 | 0.0014 | 0.0368 | 1.0009 | 1.0004 |
| Tuvalu | Women | 3 | 0.7902 | 0.0207 | 0.7467 | 0.8280 | 0.3869 | 0.3656 | 0.4054 | 0.0015 | 0.0385 | 1.0579 | 1.0286 |
| Uganda | Men | 0 | 0.1174 | 0.0132 | 0.0939 | 0.1459 | 0.0575 | 0.0460 | 0.0714 | 2.5485 | 1.5964 | 1.7319 | 1.3160 |
| Uganda | Men | 1 | 0.7452 | 0.0175 | 0.7093 | 0.7780 | 0.3648 | 0.3473 | 0.3809 | 2.4505 | 1.5654 | 1.6100 | 1.2689 |
| Uganda | Men | 2 | 0.1097 | 0.0120 | 0.0882 | 0.1356 | 0.0537 | 0.0432 | 0.0664 | 2.2485 | 1.4995 | 1.3804 | 1.1749 |
| Uganda | Men | 3 | 0.0277 | 0.0068 | 0.0170 | 0.0448 | 0.0136 | 0.0083 | 0.0219 | 2.6331 | 1.6227 | 1.8113 | 1.3458 |
| Uganda | Women | 0 | 0.0706 | 0.0087 | 0.0554 | 0.0897 | 0.0346 | 0.0271 | 0.0439 | 1.6606 | 1.2887 | 1.5053 | 1.2269 |
| Uganda | Women | 1 | 0.6205 | 0.0163 | 0.5880 | 0.6519 | 0.3038 | 0.2879 | 0.3192 | 1.6375 | 1.2797 | 1.6047 | 1.2668 |
| Uganda | Women | 2 | 0.2110 | 0.0135 | 0.1857 | 0.2386 | 0.1033 | 0.0909 | 0.1168 | 1.5814 | 1.2575 | 1.5697 | 1.2529 |
| Uganda | Women | 3 | 0.0979 | 0.0119 | 0.0769 | 0.1239 | 0.0479 | 0.0376 | 0.0607 | 2.3352 | 1.5281 | 2.2017 | 1.4838 |
| United Republic of Tanzania | Men | 0 | 0.1946 | 0.0542 | 0.1093 | 0.3226 | 0.0953 | 0.0535 | 0.1579 | 8.9818 | 2.9970 | 18.9619 | 4.3545 |
| United Republic of Tanzania | Men | 1 | 0.6451 | 0.0321 | 0.5800 | 0.7051 | 0.3158 | 0.2840 | 0.3452 | 2.1497 | 1.4662 | 3.6962 | 1.9226 |
| United Republic of Tanzania | Men | 2 | 0.1366 | 0.0348 | 0.0815 | 0.2200 | 0.0669 | 0.0399 | 0.1077 | 4.9189 | 2.2179 | 7.9381 | 2.8175 |
| United Republic of Tanzania | Men | 3 | 0.0237 | 0.0077 | 0.0125 | 0.0446 | 0.0116 | 0.0061 | 0.0219 | 1.2313 | 1.1096 | 1.3479 | 1.1610 |
| United Republic of Tanzania | Women | 0 | 0.0590 | 0.0155 | 0.0350 | 0.0979 | 0.0289 | 0.0171 | 0.0479 | 2.6469 | 1.6269 | 2.7191 | 1.6490 |
| United Republic of Tanzania | Women | 1 | 0.4842 | 0.0546 | 0.3795 | 0.5904 | 0.2371 | 0.1858 | 0.2891 | 7.2773 | 2.6977 | 11.5150 | 3.3934 |
| United Republic of Tanzania | Women | 2 | 0.2730 | 0.0412 | 0.2000 | 0.3606 | 0.1337 | 0.0979 | 0.1765 | 5.2045 | 2.2813 | 9.4873 | 3.0801 |
| United Republic of Tanzania | Women | 3 | 0.1837 | 0.0283 | 0.1345 | 0.2458 | 0.0900 | 0.0659 | 0.1204 | 3.2605 | 1.8057 | 6.9862 | 2.6432 |
| Vietnam | Men | 0 | 0.0888 | 0.0116 | 0.0685 | 0.1144 | 0.0435 | 0.0335 | 0.0560 | 16.0631 | 4.0079 | 1.8109 | 1.3457 |
| Vietnam | Men | 1 | 0.7400 | 0.0186 | 0.7019 | 0.7747 | 0.3623 | 0.3437 | 0.3793 | 17.2170 | 4.1493 | 1.9756 | 1.4056 |
| Vietnam | Men | 2 | 0.1523 | 0.0143 | 0.1262 | 0.1825 | 0.0746 | 0.0618 | 0.0894 | 15.2983 | 3.9113 | 1.7817 | 1.3348 |
| Vietnam | Men | 3 | 0.0190 | 0.0054 | 0.0109 | 0.0331 | 0.0093 | 0.0053 | 0.0162 | 15.0571 | 3.8803 | 1.7601 | 1.3267 |
| Vietnam | Women | 0 | 0.1011 | 0.0101 | 0.0830 | 0.1227 | 0.0495 | 0.0407 | 0.0601 | 11.0273 | 3.3207 | 1.7882 | 1.3373 |
| Vietnam | Women | 1 | 0.7178 | 0.0147 | 0.6881 | 0.7458 | 0.3515 | 0.3369 | 0.3651 | 10.6002 | 3.2558 | 1.5759 | 1.2553 |
| Vietnam | Women | 2 | 0.1649 | 0.0119 | 0.1429 | 0.1897 | 0.0808 | 0.0700 | 0.0929 | 10.2021 | 3.1941 | 1.4716 | 1.2131 |
| Vietnam | Women | 3 | 0.0161 | 0.0033 | 0.0107 | 0.0241 | 0.0079 | 0.0052 | 0.0118 | 6.9681 | 2.6397 | 0.8544 | 0.9243 |
| Zambia | Men | 0 | 0.0613 | 0.0098 | 0.0447 | 0.0836 | 0.0300 | 0.0219 | 0.0409 | 1.5369 | 1.2397 | 1.5677 | 1.2521 |
| Zambia | Men | 1 | 0.7546 | 0.0180 | 0.7176 | 0.7881 | 0.3694 | 0.3513 | 0.3859 | 1.6145 | 1.2706 | 1.7258 | 1.3137 |
| Zambia | Men | 2 | 0.1509 | 0.0140 | 0.1254 | 0.1805 | 0.0739 | 0.0614 | 0.0884 | 1.4143 | 1.1892 | 1.5695 | 1.2528 |
| Zambia | Men | 3 | 0.0332 | 0.0079 | 0.0208 | 0.0526 | 0.0163 | 0.0102 | 0.0258 | 1.7793 | 1.3339 | 1.8968 | 1.3773 |
| Zambia | Women | 0 | 0.0724 | 0.0079 | 0.0583 | 0.0896 | 0.0354 | 0.0285 | 0.0439 | 0.8546 | 0.9245 | 1.3360 | 1.1559 |
| Zambia | Women | 1 | 0.5844 | 0.0162 | 0.5524 | 0.6158 | 0.2861 | 0.2704 | 0.3015 | 0.9807 | 0.9903 | 1.5747 | 1.2549 |
| Zambia | Women | 2 | 0.2107 | 0.0138 | 0.1850 | 0.2390 | 0.1032 | 0.0906 | 0.1170 | 1.0339 | 1.0168 | 1.6676 | 1.2914 |
| Zambia | Women | 3 | 0.1325 | 0.0121 | 0.1104 | 0.1581 | 0.0649 | 0.0541 | 0.0774 | 1.1670 | 1.0803 | 2.0146 | 1.4194 |

# **Table E in S1 Text. Survey-adjusted and age-standardized prevalence of clinical obesity and BMI-only obesity together with the relative change (Figure 2 in manuscript) as well as the absolute change, and the best-available evidence of the prevalence of BMI-only obesity worldwide from the NCD-RisC for comparison purposes.**

| **World region** | **Country** | **Sex** | **Clinical obesity** | **BMI obesity** | **Relative change** | **Absolute change (percentage points)** | **NCD-RisC (%)*** |
| --- | --- | --- | --- | --- | --- | --- | --- |
| Eastern Mediterranean | Afghanistan | Men | 5.05% | 6.93% | -27.10% | 1.88% | 12.10 |
| Eastern Mediterranean | Afghanistan | Women | 9.04% | 12.84% | -29.63% | 3.81% | 19.20 |
| Africa | Algeria | Men | 4.92% | 8.00% | -38.53% | 3.08% | 13.30 |
| Africa | Algeria | Women | 10.72% | 17.01% | -36.99% | 6.29% | 29.90 |
| Western Pacific | American Samoa | Men | 28.78% | 36.32% | -20.75% | 7.54% | 66.10 |
| Western Pacific | American Samoa | Women | 27.87% | 39.82% | -30.01% | 11.95% | 75.90 |
| Europe | Armenia | Men | 5.95% | 7.77% | -23.43% | 1.82% | 15.90 |
| Europe | Armenia | Women | 10.81% | 14.23% | -24.07% | 3.42% | 28.00 |
| Europe | Azerbaijan | Men | 5.50% | 7.85% | -29.95% | 2.35% | 16.50 |
| Europe | Azerbaijan | Women | 10.86% | 14.36% | -24.39% | 3.50% | 32.50 |
| Southeast Asia | Bangladesh | Men | 1.01% | 1.21% | -17.08% | 0.21% | 2.30 |
| Southeast Asia | Bangladesh | Women | 3.09% | 4.44% | -30.28% | 1.34% | 5.90 |
| Europe | Belarus | Men | 9.02% | 10.75% | -16.08% | 1.73% | 18.60 |
| Europe | Belarus | Women | 13.19% | 15.96% | -17.37% | 2.77% | 24.60 |
| Africa | Benin | Men | 1.78% | 2.54% | -30.18% | 0.77% | 5.50 |
| Africa | Benin | Women | 4.10% | 5.60% | -26.76% | 1.50% | 12.70 |
| Southeast Asia | Bhutan | Men | 3.09% | 4.97% | -37.71% | 1.87% | 8.20 |
| Southeast Asia | Bhutan | Women | 5.09% | 9.06% | -43.88% | 3.98% | 13.90 |
| Africa | Botswana | Men | 2.05% | 3.46% | -40.80% | 1.41% | 6.60 |
| Africa | Botswana | Women | 7.96% | 11.52% | -30.90% | 3.56% | 24.20 |
| Western Pacific | Brunei Darussalam | Men | 13.16% | 14.96% | -12.03% | 1.80% | 24.40 |
| Western Pacific | Brunei Darussalam | Women | 13.29% | 17.55% | -24.24% | 4.25% | 28.00 |
| Africa | Cabo Verde | Men | 2.87% | 4.04% | -28.90% | 1.17% | 8.20 |
| Africa | Cabo Verde | Women | 7.87% | 13.01% | -39.53% | 5.14% | 22.20 |
| Western Pacific | Cambodia | Men | 0.37% | 0.51% | -26.60% | 0.13% | 1.50 |
| Western Pacific | Cambodia | Women | 0.73% | 1.33% | -45.27% | 0.60% | 2.50 |
| Africa | Comoros | Men | 2.14% | 3.69% | -42.02% | 1.55% | 4.70 |
| Africa | Comoros | Women | 9.92% | 15.37% | -35.43% | 5.44% | 16.40 |
| Western Pacific | Cook Islands | Men | 28.88% | 33.90% | -14.80% | 5.02% | 60.10 |
| Western Pacific | Cook Islands | Women | 26.74% | 36.04% | -25.81% | 9.30% | 67.90 |
| Americas | Ecuador | Men | 7.41% | 10.82% | -31.50% | 3.41% | 19.60 |
| Americas | Ecuador | Women | 11.55% | 16.96% | -31.89% | 5.41% | 30.50 |
| Africa | Eritrea | Men | 0.25% | 0.43% | -42.80% | 0.18% | 1.40 |
| Africa | Eritrea | Women | 0.82% | 1.46% | -43.66% | 0.64% | 3.50 |
| Africa | Eswatini | Men | 3.91% | 6.01% | -34.92% | 2.10% | 10.40 |
| Africa | Eswatini | Women | 12.78% | 19.77% | -35.32% | 6.98% | 38.50 |
| Africa | Ethiopia | Men | 0.21% | 0.32% | -32.12% | 0.10% | 0.70 |
| Africa | Ethiopia | Women | 0.76% | 1.31% | -42.06% | 0.55% | 3.00 |
| Western Pacific | French Polynesia | Men | 15.74% | 21.90% | -28.12% | 6.16% | 41.40 |
| Western Pacific | French Polynesia | Women | 16.39% | 23.59% | -30.53% | 7.20% | 45.00 |
| Europe | Georgia | Men | 13.26% | 17.46% | -24.02% | 4.19% | 28.40 |
| Europe | Georgia | Women | 15.04% | 18.85% | -20.21% | 3.81% | 33.40 |
| Eastern Mediterranean | Iraq | Men | 10.29% | 15.44% | -33.38% | 5.15% | 29.70 |
| Eastern Mediterranean | Iraq | Women | 18.32% | 24.06% | -23.82% | 5.73% | 44.50 |
| Africa | Kenya | Men | 2.09% | 3.07% | -31.76% | 0.97% | 4.60 |
| Africa | Kenya | Women | 4.25% | 8.20% | -48.22% | 3.95% | 14.50 |
| Western Pacific | Kiribati | Men | 12.64% | 16.84% | -24.94% | 4.20% | 35.10 |
| Western Pacific | Kiribati | Women | 19.65% | 29.21% | -32.71% | 9.55% | 53.80 |
| Eastern Mediterranean | Kuwait | Men | 14.53% | 19.98% | -27.27% | 5.45% | 37.30 |
| Eastern Mediterranean | Kuwait | Women | 17.06% | 24.08% | -29.16% | 7.02% | 49.50 |
| Eastern Mediterranean | Kyrgyzstan | Men | 6.86% | 8.55% | -19.71% | 1.68% | 18.40 |
| Eastern Mediterranean | Kyrgyzstan | Women | 11.30% | 14.31% | -21.01% | 3.01% | 24.20 |
| Western Pacific | Lao People's Democratic Republic | Men | 0.92% | 1.78% | -48.12% | 0.86% | 3.70 |
| Western Pacific | Lao People's Democratic Republic | Women | 2.90% | 4.03% | -28.13% | 1.13% | 6.60 |
| Eastern Mediterranean | Lebanon | Men | 14.66% | 16.53% | -11.33% | 1.87% | 27.90 |
| Eastern Mediterranean | Lebanon | Women | 10.38% | 14.02% | -25.95% | 3.64% | 29.90 |
| Africa | Lesotho | Men | 1.97% | 3.92% | -49.73% | 1.95% | 5.20 |
| Africa | Lesotho | Women | 8.47% | 16.18% | -47.68% | 7.72% | 26.70 |
| Eastern Mediterranean | Libya | Men | 7.36% | 10.78% | -31.76% | 3.42% | 22.00 |
| Eastern Mediterranean | Libya | Women | 11.84% | 20.19% | -41.35% | 8.35% | 41.20 |
| Africa | Malawi | Men | 0.22% | 0.69% | -67.68% | 0.47% | 2.40 |
| Africa | Malawi | Women | 2.63% | 5.57% | -52.79% | 2.94% | 9.40 |
| Western Pacific | Mongolia | Men | 7.98% | 10.07% | -20.73% | 2.09% | 20.00 |
| Western Pacific | Mongolia | Women | 9.18% | 12.48% | -26.50% | 3.31% | 25.60 |
| Eastern Mediterranean | Morocco | Men | 3.50% | 6.40% | -45.39% | 2.91% | 12.40 |
| Eastern Mediterranean | Morocco | Women | 9.11% | 16.45% | -44.60% | 7.33% | 27.50 |
| Southeast Asia | Myanmar | Men | 1.35% | 1.70% | -20.77% | 0.35% | 3.90 |
| Southeast Asia | Myanmar | Women | 3.22% | 4.02% | -19.94% | 0.80% | 7.70 |
| Western Pacific | Nauru | Men | 24.15% | 37.45% | -35.51% | 13.30% | 67.40 |
| Western Pacific | Nauru | Women | 21.78% | 36.94% | -41.05% | 15.16% | 71.00 |
| Southeast Asia | Nepal | Men | 1.81% | 2.25% | -19.51% | 0.44% | 4.20 |
| Southeast Asia | Nepal | Women | 2.10% | 3.31% | -36.45% | 1.20% | 7.50 |
| Western Pacific | Niue | Men | 23.11% | 32.95% | -29.88% | 9.85% | 58.60 |
| Western Pacific | Niue | Women | 22.73% | 33.14% | -31.40% | 10.40% | 63.30 |
| Eastern Mediterranean | Qatar | Men | 10.63% | 20.93% | -49.23% | 10.30% | 33.90 |
| Eastern Mediterranean | Qatar | Women | 16.81% | 26.69% | -37.03% | 9.88% | 45.20 |
| Europe | Republic of Moldova | Men | 8.68% | 10.70% | -18.88% | 2.02% | 19.40 |
| Europe | Republic of Moldova | Women | 13.12% | 15.84% | -17.15% | 2.72% | 27.50 |
| Africa | Rwanda | Men | 0.37% | 0.50% | -25.58% | 0.13% | 0.90 |
| Africa | Rwanda | Women | 1.26% | 2.65% | -52.39% | 1.39% | 4.00 |
| Western Pacific | Samoa | Men | 17.90% | 26.13% | -31.51% | 8.23% | 47.00 |
| Western Pacific | Samoa | Women | 19.34% | 36.01% | -46.29% | 16.67% | 69.20 |
| Africa | Sao Tome and Principe | Men | 3.62% | 3.94% | -8.05% | 0.32% | 8.90 |
| Africa | Sao Tome and Principe | Women | 10.45% | 11.68% | -10.52% | 1.23% | 22.00 |
| Africa | Seychelles | Men | 6.19% | 7.33% | -15.51% | 1.14% | 12.50 |
| Africa | Seychelles | Women | 13.51% | 17.19% | -21.39% | 3.68% | 31.80 |
| Western Pacific | Solomon Islands | Men | 5.79% | 10.31% | -43.81% | 4.52% | 16.10 |
| Western Pacific | Solomon Islands | Women | 9.09% | 15.28% | -40.52% | 6.19% | 25.50 |
| Southeast Asia | Sri Lanka | Men | 1.22% | 1.83% | -33.51% | 0.61% | 4.00 |
| Southeast Asia | Sri Lanka | Women | 3.12% | 4.65% | -32.87% | 1.53% | 9.20 |
| Eastern Mediterranean | Sudan | Men | 2.53% | 3.69% | -31.50% | 1.16% | 8.00 |
| Eastern Mediterranean | Sudan | Women | 6.29% | 8.84% | -28.87% | 2.55% | 19.40 |
| Europe | Tajikistan | Men | 4.94% | 6.53% | -24.33% | 1.59% | 0.90 |
| Europe | Tajikistan | Women | 6.67% | 9.67% | -31.05% | 3.00% | 1.80 |
| Southeast Asia | Timor Leste | Men | 0.26% | 0.47% | -45.97% | 0.22% | 16.70 |
| Southeast Asia | Timor Leste | Women | 0.53% | 0.95% | -43.75% | 0.41% | 24.40 |
| Africa | Togo | Men | 1.53% | 2.19% | -30.37% | 0.67% | 3.50 |
| Africa | Togo | Women | 5.19% | 8.76% | -40.72% | 3.56% | 10.00 |
| Western Pacific | Tokelau | Men | 28.79% | 33.96% | -15.21% | 5.16% | 63.40 |
| Western Pacific | Tokelau | Women | 26.65% | 37.14% | -28.23% | 10.48% | 71.60 |
| Europe | Turkmenistan | Men | 5.12% | 7.63% | -32.86% | 2.51% | 17.60 |
| Europe | Turkmenistan | Women | 7.04% | 10.06% | -30.02% | 3.02% | 23.30 |
| Western Pacific | Tuvalu | Men | 20.35% | 29.83% | -31.78% | 9.48% | 54.20 |
| Western Pacific | Tuvalu | Women | 27.81% | 38.69% | -28.12% | 10.88% | 69.40 |
| Africa | Uganda | Men | 0.97% | 1.36% | -28.39% | 0.39% | 1,9 |
| Africa | Uganda | Women | 2.87% | 4.79% | -40.17% | 1.93% | 7.60 |
| Africa | United Republic of Tanzania | Men | 0.85% | 1.16% | -26.94% | 0.31% | 3.90 |
| Africa | United Republic of Tanzania | Women | 5.76% | 9.00% | -35.96% | 3.23% | 11.60 |
| Western Pacific | Vietnam | Men | 0.53% | 0.93% | -43.06% | 0.40% | 1.00 |
| Western Pacific | Vietnam | Women | 0.50% | 0.79% | -36.65% | 0.29% | 1.20 |
| Africa | Zambia | Men | 1.21% | 1.63% | -25.78% | 0.42% | 3.80 |
| Africa | Zambia | Women | 3.67% | 6.49% | -43.46% | 2.82% | 13.60 |

Relative change was computed as (Clinica Obesity - BMI Obesity)/BMI Obesity and expressed as a percentage. Absolute change was computed as BMI Obesity - Clinical Obesity and should be interpreted as a change in percentage points. *Matched by exact year (<https://ncdrisc.org/obesity-prevalence-ranking.html>); the NCD-RisC estimates age age-standardized for adults aged 20 years and above.

# **Flowchart A in S1 Text. Analytical sample.**

Download dataset

(n=287,123)

Excluding datasets without glucose measurements (n=258,330)

Excluding datasets without lipid measurements (n=244,018)

Excluding datasets without blood pressure measurements (n=244,018)

Excluding datasets without anthropometrics (n=239,221)

Excluding datasets without sampling design variables (n=230,846)

Excluding outliers and missing (n=142,250)
